# Structure-Based Design, Synthesis and Bioactivity of a New Anti-TNFα Cyclopeptide

**DOI:** 10.3390/molecules25040922

**Published:** 2020-02-19

**Authors:** Mohannad Idress, Bruce F. Milne, Gary S. Thompson, Laurent Trembleau, Marcel Jaspars, Wael E. Houssen

**Affiliations:** 1 Institute of Medical Sciences, University of Aberdeen, Ashgrove Road West, Aberdeen AB25 2ZD, UK; mohannad.idress@outlook.com; 2 Marine Biodiscovery Centre, Department of Chemistry, University of Aberdeen, Meston Walk, Aberdeen AB24 3UE, UK; l.trembleau@abdn.ac.uk (L.T.); m.jaspars@abdn.ac.uk (M.J.); 3 CFisUC, Department of Physics, University of Coimbra, Rua Larga, 3004-516 Coimbra, Portugal; bfmilne@gmail.com; 4 Wellcome Trust Biomolecular NMR Facility, School of Biosciences, University of Kent, Canterbury, Kent, England CT2 7NZ, UK; g.s.thompson@kent.ac.uk

**Keywords:** cyclic peptides, NMR structure, drug design, protein–protein interaction

## Abstract

As opposed to small molecules, macrocyclic peptides possess a large surface area and are recognised as promising candidates to selectively treat diseases by disrupting specific protein–protein interactions (PPIs). Due to the difficulty in predicting cyclopeptide conformations in solution, the de novo design of bioactive cyclopeptides remains significantly challenging. In this study, we used the combination of conformational analyses and molecular docking studies to design a new cyclopeptide inhibitor of the interaction between the human tumour necrosis factor alpha (TNFα) and its receptor TNFR-1. This interaction is a key in mediating the inflammatory response to tissue injury and infection in humans, and it is also an important causative factor of rheumatoid arthritis, psoriasis and inflammatory bowel disease. The solution state NMR structure of the cyclopeptide was determined, which helped to deduce its mode of interaction with TNFα. TNFα sensor cells were used to evaluate the biological activity of the peptide.

## 1. Introduction

Tumour necrosis factor alpha (TNFα) is a pleiotropic inflammatory cytokine produced and secreted mainly by macrophages, which can also be produced by many other cells, for instance, CD4+ lymphocytes, neutrophils, NK cells and mast cells [1]. TNFα plays an important role in the pathogenesis of many chronic inflammatory diseases and its inhibition has been shown to be an effective approach for therapy of rheumatoid arthritis, psoriatic arthritis, ankylosing spondylitis, psoriasis and inflammatory bowel disease [2]. The systemic inflammatory effects of TNFα are caused by its soluble form, which exists as a homo-trimeric complex of 17-kDa subunits. It binds to two different receptors: TNF Receptor-1 (TNFR-1), also known as [p55 (CD120a)], and TNF Receptor-2 (TNFR-2) [or p75 (CD120b)], to which TNFα binds with relatively higher affinity compared to TNFR-1 [3].

Binding of either TNFR-1 or 2 to their ligand induces the receptor’s trimerisation. Both receptors bind TNFα, with TNFR-1 being constitutively expressed in all cells, while TNFR-2 is inducibly regulated mainly in haematopoietic and endothelial cells [4,5]. The TNFα-mediated clustering of TNFR-1 initiates the signalling of pathways that lead to both apoptosis (through the caspase cascade) and cell activation (through NF-ĸB) [6]. The NF-ĸB activation pathway following TNFα binding also induces pro-inflammatory cytokines like IL-4 and IL-8, responsible for the extensive tissue damage seen in many chronic inflammatory diseases [7].

Small linear peptides (8–10 amino acids) are not appropriate as mimetics of the defined secondary structures found in proteins, due to their intrinsic conformational flexibility. By contrast, the structural rigidity of cyclic peptides makes them good mimetics of the protein conformational epitopes that lead to interactions with proteins and ligands [8]. A common strategy to rigidify peptide structures is by cyclisation, which could be via disulphide bridge formation or amide bond formation between *N*- and *C*- termini. *N*- to *C*- cyclisation has the advantage that it usually leads to fewer rotatable bonds when compared to disulphide bridge formation. Therefore, *N*- to *C*- cyclisation usually results in more rigid and less flexible conformers [9].

A range of biologics targeting TNFα have been licensed for the treatment of various inflammatory diseases, such as rheumatoid arthritis and Crohn’s disease [10]. Although these agents have high potency and specificity, they are limited by their high production costs and potential immunogenicity. For instance, large molecule biologics like infliximab have been associated with development of infusion reaction (antigenicity) despite their high selectivity for TNFα [11]. Also, their large molecular weight and low oral stability means their administration is only feasible through injection [12]. Several small molecule synthetic inhibitors of TNFα are at different stages of pre-clinical and clinical development. Thalidomide was the first to be developed and it inhibits TNFα production by promoting TNFα mRNA degradation in stimulated monocytes [13]. This was followed by the discovery of other classes of inhibitors targeting p38 MAP kinase, which controls TNFα production, TNFα converting enzyme (TACE), and the mRNA coding for TNFα. The majority of these agents exhibited toxicities related to their ability to cross the blood-brain barrier or by inhibition of liver cytochrome P450 enzyme [14]. Generally, the molecular size and lipophilicity of peptides determines their ability to cross the physical and enzymatic barriers of the brain [15]. Peptide cyclisation as a means of overcoming these two different types of barriers has only been successful with small opioid cyclopeptides (tetramers) possessing a β-turn [16].

Small molecule with anti-TNFα activity with compact 3D conformations containing trifluoromethylphenyl indole and chromone moieties have previously been designed. These compounds bind with high affinity (IC_50_ = 13 μM) to a hydrophobic binding pocket formed by six tyrosine residues (Y59, Y119 and Y151 from chain A and B) at the interface of TNFα dimer [17]. Alternative binding pockets that bind small molecules have also been identified from the predicted TNFα-TNFR1 complex and these involve the residues H66, L67 and L71 of TNFR1 and residues R82, F90 and F127 in TNFα [18]. Recently, a small molecule benzimidazole derivative (UCB-6876) has been discovered, a co-crystal structure of UCB-6876 with TNFα resulted in a change in the spatial arrangement of the TNFα trimer and a loss of its 3-fold symmetry. This compound was also found to occupy the centre of the TNFα trimer filling a hydrophobic binding pocket involving chain A (Y59), chain B (L57, Y119) and chain C (Y59, Y151) [19].

## 2. Results and Discussion

In an attempt to discover improved anti-TNFα therapeutics, we designed a cyclic peptide that could directly inhibit the TNFα-TNFR1 interaction and have potentially fewer side effects than the small molecule inhibitors and biologics mentioned above. This was achieved by producing a cyclic peptide with higher target specificity compared to small molecules and low potential of immunogenicity when compared with biologics. Protein loops are often involved in the biomolecular recognition of proteins [20]. Loop peptidomimetics have previously been introduced as a new class of bioactive agents. The cyclic peptide cyclo-GCRLYGFKIHGCG derived from the regulatory subunit (CK2β) of protein kinase 2 which inhibits the interaction with the associated catalytic subunit and is used for cancer therapy [21]. Another example is the macrocyclic peptidomimetic MCP-1, which is an inhibitor of the menin-MLL protein–protein interaction involved in leukemogenesis [22].

**Figure 1:**
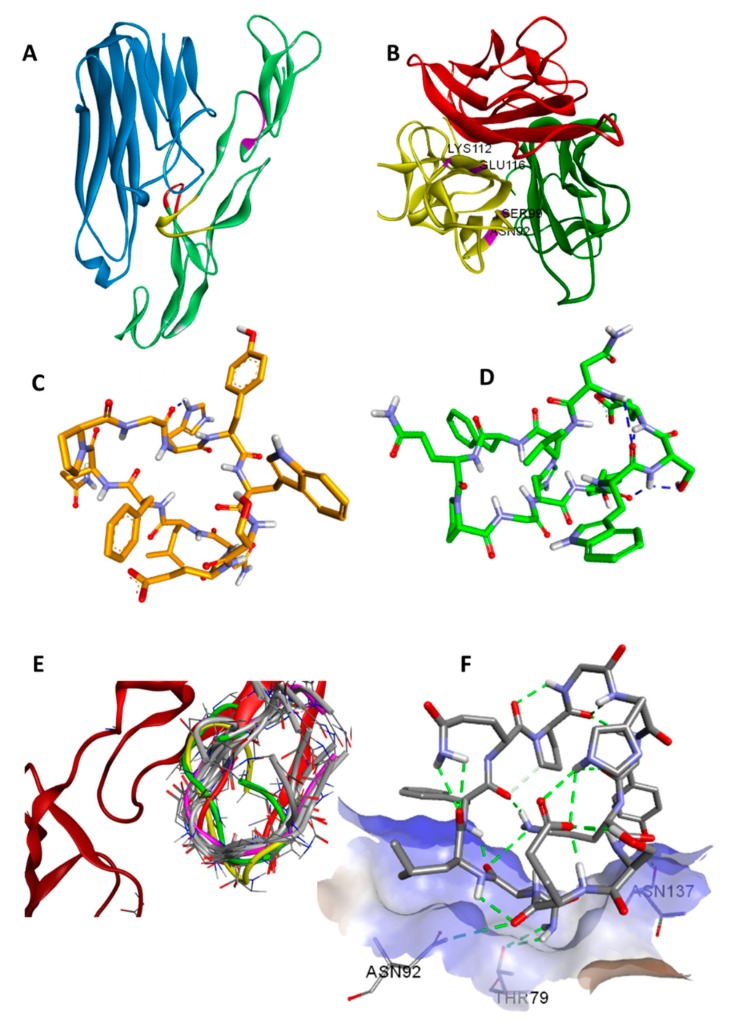
Modelling of the inhibition of TNFα-TNFR1 interaction. (**A**) This figure shows the different binding sites of TNFR1 based on the available crystal structure of TNFβ/TNFR-1 complex [PDB: 1TNR]. The TNFβ structure (blue) bound to TNFR-1 (green) with highlighted 3 TNFR1 binding sites, WP5 (magenta), WP8 (yellow) and WP9 (red). (**B**) 3D view of TNFα homo-trimer (PDB: 1TNF) showing residues N92, S99, K112 and E116 (magenta) at the interface that are important for the formation of the TNFα bioactive complex. The lowest energy NMR structures of **1** are clustered into two main groups, a major group of seven structures, in which (**C**) is the lowest energy conformation and a minor group of three structures in which (**D**) is the lowest conformation in the cluster. (**E**) illustrates structural similarity between different structures of **1** and WP9 domain. Aligned and superimposed docking poses and NMR structures of **1** against the WP9 loop of TNFR-1 are shown in red to illustrate their structural similarity. Docking pose 6 is shown in purple, and major and minor NMR structures are coloured in yellow and green, respectively. (**F**) WP9 loop peptidomimetic **1** docked to TNFα (PDB: 1TNF). The interaction involves four hydrogen bonds with N137, N92 and T79 residues of TNFα; details can be found in Table S1.

Structure-based design of exocyclic peptidomimetic inhibitors of TNFα prepared by Takasaki and colleagues provided a good starting point to develop PPI inhibitors [23]. The two TNFR-1 binding sites (WP8 and WP9) were known from the structural information available for the TNFβ/TNFR-1 complex (Figure 1A) [24]. The importance of the WP5 binding site was inferred from the light chain sequences of several TNFα neutralising antibodies which have been shown to contribute to TNFR-1 binding. This data was further supported by the structural homology of the modelled light chain structure (CDR1) of anti-TNFα with the WP5 site [25].

**Figure 2:**
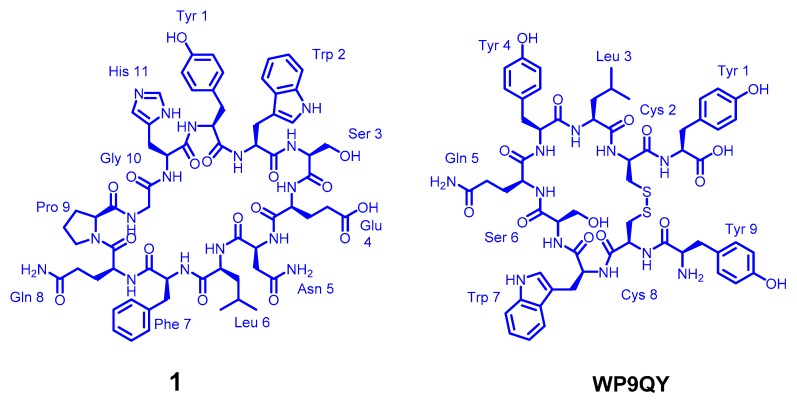
The chemical structure of the cyclic peptides **1** and WP6QY.

The design of a new anti-TNFα cyclic peptide **1** (Figure 2) was achieved by extending the 5 residues (WSENL) of the WP9 loop used to design WP9QY (Figure 2) to the 9 residues [105 HYWSENLFQ 113] which were cyclised head to tail via a proline-glycine linker to help stabilise the loop structure in an 11mer cyclic peptide; shown in (Table 1). This cyclic peptide showed enhanced binding to TNFα evaluated by molecular docking (Table S1). The anti-TNFα activity of this new macrocyclic peptidomimetic of the WP9 binding site **1** in comparison with the known peptide WP9QY (purchased from Almac, Craigavon, UK) was determined by an in vitro cell-based assay [26]. The corresponding linear peptide of **1** was not evaluated because of the potential for off target binding due to the high conformational flexibility of linear peptides.

A Monte Carlo search of the conformational space of **1** was performed to provide candidate structures for subsequent protein–ligand docking so as to avoid the docking procedure being biased by a single low-energy input structure. The structures obtained in this way were then docked to TNFα resulting in docking scores of around −5 kcal/mol as shown in the Supplementary Material, Table S1. Docking results showed that **1** interacts mainly with four residues of TNFα, namely N92, S99, K112 and E116, in the WP9 site. These residues were found to also mediate the interaction at the interface between TNFα monomers during the spontaneous polymerisation as observed in the crystal structure PDB: 1TNF (Figure 1B) [27]. Consequently, this might suggest an additional mechanism of TNFα inhibition by **1** which occurs by disrupting the formation of the biologically active homo-trimer of TNFα.

The homo-multimerisation mechanism was first shown to be biologically relevant to the anti-TNFα activity of suramin, a polysulphonated naphthylurea which has been used previously to treat trypanosomiasis and onchocerciasis. Deoligomerisation of TNFα by suramin was shown by measuring the cell-bound radioactivity of cultured K562 cells incubated with ^125^I-labelled TNFα in the presence or absence of suramin [28]. Modelling of a suramin structural analogue bound to TNFα has led to prediction of the residues involved in the interaction. These include K112 and E116 which have been shown to interact with **1** as well as other residues including T72, K98 and R103 [29].

The synthesis of **1** was performed using a Fmoc-based SPPS protocol with a cyclisation yield of 33.1% at 98.4% purity. The chemical structure of **1** was confirmed by MS/MS and 2D NMR experiments (Supplementary Materials). NMR experiments were carried out in methanol-*d*_3_ due to the low solubility of **1** in water. The three-dimensional NMR structure of **1** was determined by NOE-restrained molecular dynamics (27 NOE restraints) in which the lowest energy structures were found to be clustered into two groups (A and B) with 7 converged structures in the main group (A) and 3 in a minor group (B). The RMSD of the aligned backbones of the best structure in each group was 3.05 Å. The data showing detailed chemical analysis can be found in the Supplementary Materials. The lowest energy NMR structure in each cluster are shown in (Figure 1C,D).

A molecular similarity study of WP9 loop of TNFR-1 with NMR structures and docking poses of **1** was performed by alignment and superimposition of backbone atoms (Table 2, Figure 1E). The structural resemblance observed between the WP9 loop and the best docking pose (conformation 6) can be seen from the low RMSD value (backbone) of 2.09 Å observed. This might suggest a mechanism of inhibition for **1** in which **1** binds to nearby residues in the WP9 binding site of TNFα and abolish the TNFR-1 interaction (Figure 1F). Furthermore, the lowest energy NMR structure of the main cluster (structure A) adopts a similar conformation to the WP9 loop of TNFR-1 (RMSD = 2.68) and again suggesting an analogous binding mode to WP9QY. However, the polarity of the solvent used for NMR could also contribute to the observed difference in conformation [30].

**Figure 3:**
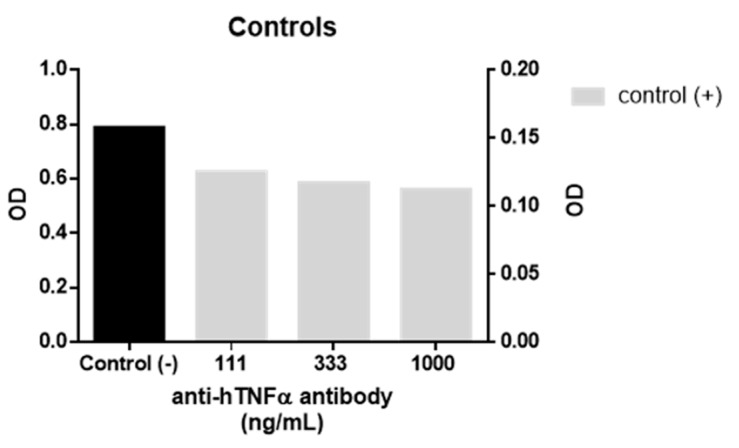
Controls used in TNFα assay as a measure of HEK-Blue cells response to TNFα. Negative control response to TNFα was used to normalise inhibitors’ response to inhibition (%). Anti-hTNFα antibody was used as positive control with significant difference in response following T test (*P* < 0.0001). Error bars are not visible due to the small size of the calculated standard errors.

A biological assay using a TNFα sensor cell (HEK-Blue TNFα cells, InvivoGen, Toulouse, France) was used to evaluate the anti-TNFα activity of **1**. In the assay, the cells are transfected with a secretory alkaline phosphatase reporter gene which is downstream of the NF-ĸB promoter and expressed in response to TNFα receptor activation. The alkaline phosphatase activity was then quantified by measuring the absorbance (λ = 620 nm) of coloured substrate. For each assay, approximately 5 × 10^4^ cells were seeded in each well of a 96 well flat-bottomed cell culture plate and incubated for 24 h with 1 ng/mL of recombinant human TNFα and different concentrations of both **1** and WP9QY in duplicates. Activation of HEK-Blue TNFα cells by 1 ng/mL of human TNFα (InvivoGen) in the presence and absence of anti-hTNFα antibody (InvivoGen) were used as positive and negative controls in the anti-TNFα assay (Figure 3). This assay was used as a direct measure of TNFα inhibition effect on cells, while the affinity of TNFα binding was evaluated by molecular docking results.

**Figure 4:**
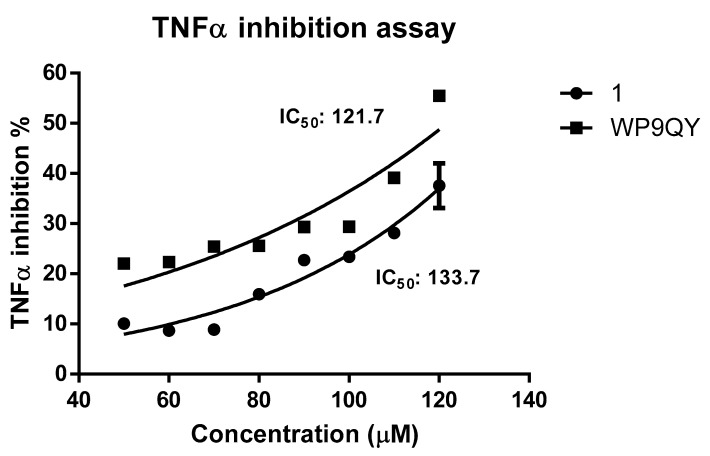
Dose-response curve of anti-TNFα activities of **1** and WP9QY. This assay was performed with one duplicate, and the error bar represent standard error. Non-linear regression was used to fit the curve to the measured data points. While measurements for **1** are well-fit, the WP9QY data shows slight deviations from those expected, this may be due to a different mode of action.

A dose-response curve for TNFα inhibition by both **1** and WP9QY was generated by normalising the absorbance readings at 620 nm against dose to percentage. This resulted in IC_50_ of 133.7 μM (by extrapolation) and of 121.7 µM for **1** and WP9QY, respectively (Figure 4). In this assay, the inhibition of TNFα activity by 35% is achieved by 117.4 µM and 97.2 µM for **1** and WP9QY respectively. This result contrasts with those of Takasaki and co-workers, where the same level of TNFα inhibition was reached by approximately 2.5 µM of WP9QY when tested by using U937 cells in a flow cytometric assay [23]. Their flow cytometric assay measures anti-TNFα activity as was determined by inhibition of anti-TNF receptor antibody binding. Bound anti-TNF receptor antibody was then measured by its binding to fluorescein-labelled secondary antibody which does not represent direct interaction between TNFα and its receptor, whereas the assay used here quantifies TNFα response directly.

The assay used in this study is considered more specific for TNFα and measures TNFα activity directly and therefore provides more accurate results. Furthermore, the use of reporter gene inducible by TNFα gives more evidence as to whether the peptide binding would have agonistic or antagonistic effects downstream of the TNFα signalling pathway. Peptides were dissolved in DMSO and solutions were diluted with the cell culture media so the final concentration of DMSO is 1%. Blank experiments with 1% DMSO were carried out. The difference in IC_50_ between **1** and WP9QY reported here could also be attributed to a different mode of action of **1** such as deoligomerisation of TNFα homo-trimer by **1** in which the binding to TNFα take place in 1:3 ratio with WP9QY binding stoichiometrically to block TNFR-1 interaction of the oligomer. This alternative mode of action (deoligmerisation) was proposed based on our docking results (Table S1), which shows the interaction of **1** with TNFα residues N92, S99, K112 and E116. These are key residues mediating interfacial interaction between TNFα monomers to form the biologically active trimer as illustrated in Figure 1B.

**Figure 5:**
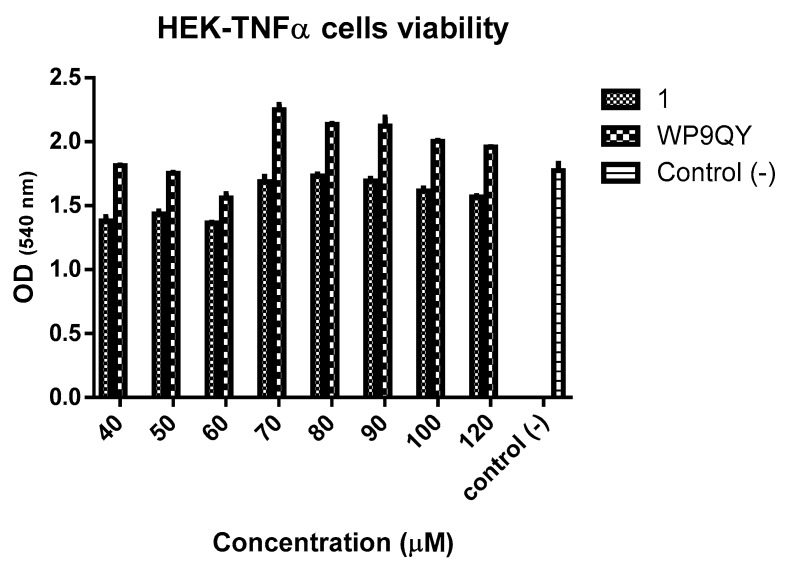
MTT assay of HEK-Blue TNFα cells in the presence of 1 and WP9QY. Optical density (OD) readings at 540 nm correspond to formazan levels formed by viable cells after incubation with **1** and WP9QY for 24 h. This assay was performed with one duplicate, and the error bars show standard error.

An MTT assay (Figure 5) showed no significant cytotoxicity to HEK-Blue TNFα cells at the concentration used to test anti-TNFα activity of both **1** and WP9QY. Therefore, this can be used as preliminary data for further biological evaluations.

## 3. Materials and Methods

Fmoc amino acid derivatives, coupling reagents (HBTU, HATU and HOBt), preloaded chlorotrityl resin (H-Gly 2-ClTrt), *sym*-collidine, trifluoroacetic acid (TFA), dichloromethane (DCM) and acetonitrile (HPLC grade) were purchased from Merck (Feltham, UK). *N*,*N*-dimethylformamide (DMF), piperidine, 1,1,1,3,3,3-Hexafluoro-2-propanol (HFIP), *N*,*N*-diisopropylethylamine (DIEA) and triisopropylsilane (TIS) were supplied by Alfa Aesar (Lancashire, UK).

A conformational search for thermally accessible structures of **1** was done using the Tinker molecular modelling package [31]. A set of initial cyclic peptide structures for use in the following protein-ligand docking studies was generated by Tinker’s *monte* Monte-Carlo program using the AMBER99 force field. 5000 steps of Cartesian moves and maximum atom displacement of 3 Å (default) was used. The temperature was set to 500 K and a maximum RMS gradient for truncated Newton minimisation of 0.01 (default) used. Final potential energies for each optimised conformation was then calculated in kcal/mol using Tinker’s *analyze* executable. The Boltzmann distribution of conformational ensemble obtained in this way was determined by calculating the Boltzmann factor for each structure by the following equation:

(1) $$BF=\exp\left(-\frac{E}{RT}\right)$$

where *BF* is the Boltzmann factor, *E* is the total potential energy in kJ/mol, *R* is the gas constant (8.314 J/K/mol), and *T* is the temperature in kelvin. The ratios of Boltzmann factors for all selected conformations relative to the highest energy conformation were then calculated. Structural comparison by molecular overlay (all atoms) for the resulting conformations was carried out by determining root mean square deviations (RMSDs) in Angstroms for each structure with reference to the lowest energy conformation. This was done in Discovery Studio Visualizer and all conformations of **1** were docked to TNFα using AutoDock Vina.

Side chain-protected linear peptide sequence of **1** was synthesised manually using solid phase peptide synthesis strategy (SPPS). H-Gly 2-ClTrt resin (loading of 1.1 mmol/g and 0.3 g) was first swelled in DMF for 20 min, and the construction of the peptide sequence was done through cycles of Fmoc deprotection of attached amino acids and HBTU coupling of the next amino acid in the sequence. Fmoc deprotection was carried out using 20% piperidine while coupling was done by mixing molar ratio of amino acid:HBTU:HOBt:DIEA:preloaded resin of 3:3:3:6:1 rotated for 1 h at room temperature. Reaction progress was monitored by LC-MS. Completed linear peptide sequence was cleaved from the solid support with 20% HFIP in DCM, dried under vacuum and used for cyclisation reactions. Macrocyclisation of synthesised linear peptide was carried in DMF under dilution condition (0.1 mM) using 1.5 equivalents of HATU, HOBt and *sym*-collidine slowly added to vigorously stirred peptide solution at room temperature for 24 h. Crude reaction product was then dried and deprotected using a mixture of 95% TFA, 2.5% TIS and 2.5% water [32]. Product obtained was then purified by reverse-phase HPLC with 5% to 100% gradient of acetonitrile/water on C-18 column (ACE, HiChrom, 250 mm × 10 mm i.d) followed by lyophilisation. Purified cyclic peptide was then analysed by HPLC, LC-MS and 2D NMR experiments.

Three-dimensional NMR structure of **1** was calculated in methanol-*d*3 solution using NOE-restrained molecular dynamics. The experimental NOE restraint values were directly used in calculation rather than distances. Here we used NOE restraints calculated from NOESY cross-peak volume with reference to known distances in the molecule. Cross-peak volumes were calculated using MestReNova software and NOE restraints by the following equation:

(2) $$r_{ij}=r_{ref}{\left(\frac{\alpha_{ref}}{\alpha_{ij}}\right)}^{\frac{1}{6}}$$

where, *r_ij_* and *α_ij_* are distance and peak volume demanded respectively, and *r_ref_* and *α_ref_* represent distance and peak volume of reference, respectively. The structure calculations were carried out using XPLOR-NIH by first generating random structures of the cyclic peptide. Then, simulated annealing was performed by heating the molecules to 3500 K and slowly cooled down to 100 K through 60,000 cooling steps. During simulated annealing, the NOE restraints were used to drive the conformational ensemble of cyclic peptides. The trans configuration of residue P9 was also taken into consideration during the calculations. This was determined by a low ^13^C chemical shift difference of 4.27 ppm between Cβ and Cγ [30].

TNFα sensor cells (HEK-Blue) were grown in Dulbecco’s Modified Eagle’s medium (DMEM) containing 4.5 g/L glucose and supplemented with 10% [*v*/*v*] heat-inactivated foetal bovine serum, 2 mM L-glutamine, 100 μg/mL normocin, 50 U/mL penicillin and 50 μg/mL streptomycin. Selective antibiotics added to the culture medium include 100 μg/mL zeocin and 1 μg/mL puromycin and avoided in initial cultures (first 2 passages). Maintenance cultures were kept in T-75 cell culture flasks and incubated in humidified incubator with 5% [*v*/*v*] CO_2_ at 37 °C. HEK-Blue cultures reaching 70%–80% confluency were passaged by first washing and detaching the cells gently with Dulbecco’s phosphate buffered saline. Cells were then pelleted by centrifugation at 1200 rpm for 5 min. Supernatant was discarded and cell pellets resuspended in fresh media and used in different assays.

The Vybrant^®®^ MTT assay (Thermo Fisher Scientific) was performed following the manufacturer protocol. In this assay, cell permeable tetrazolium dye (yellow) was oxidised by mitochondrial oxidases in viable cells to yield the water-insoluble formazan (purple) after incubation with HEK-TNFα cells. Formazan was then solubilised and quantified by measuring the UV absorbance at 540 nm.

## 4. Conclusions

The rational design strategy used in this study led to the discovery of a new moderate inhibitor of TNFα-TNFR-1 interaction. This cyclic peptide will be used at starting point for the development of more effective. The modelling steps, as well as the study of NMR solution structure, helped in suggesting a mode of action for **1** by correlating the structural similarity between **1** and the WP9 loop in TNFR-1. The *N*- to *C*- macrocyclic structure of **1** may be expected to confer better chemical stability and bioactivity compared to the disulphide bridged peptidomimetic (WP9QY).

## Figures and Tables

**Table 1:** Sequence comparison of original WP9 loop and anti-TNFα peptidomimetics.

Peptidomimetics	Sequence
Original WP9 Loop	HYWSENLFQ
	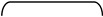
1	HYWSENLFQP
	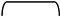
WP9QY	YCWSQYLCY

**Table 2:** RMSDs for aligned and superimposed docking poses and NMR structures of **1** with reference to WP9 loop.

Structure	RMSD (Å) (Main Chain)
WP9 domain of TNFR-1	Reference
Docking pose 6	2.09
Docking pose 5	2.27
Docking pose 2	2.44
NMR structure A	2.68
Docking pose 4	2.71
Docking pose 3	2.88
NMR structure B	3.50
Docking pose 1	4.10

## Supplementary Materials

The following are available online, containing data for molecular modelling, LC-MS and NMR analyses and further details about of TNFα sensor cells.
