# Supplementary material for: Structure-Based Design, Synthesis and Bioactivity of a New Anti-TNFα Cyclopeptide
Source: Molecules. 2020 Feb 19;25(4):922. doi: 10.3390/molecules25040922 (PMC7071015; doi:10.3390/molecules25040922)
Supplement: Supplementary file 1 [file molecules-25-00922-s001.pdf]

Structure-based design, synthesis and bioactivity of a new anti-TNFα cyclopeptide

Supplementary Material

Contents

Structure-based design of a new anti-TNFα cyclopeptide 1

Molecular modelling 2

Peptide synthesis and analysis 3

Assignments of NMR data to the structure of [1] 7

NMR 3D structure of [1] 54

TNFα sensor cells 65

Molecular modelling

Table S1: Molecular modelling results of TNFR-1 WP9 loop peptidomimetic [1].

Conformations of [1]	Total Potential Energy (Kcal/mol)	Boltzmann ratio	RMSD (Å)	Interaction	Type of interaction	Docking score (Kcal/mol)
1	-144.06	1	6.97	Trp2- $^{\varepsilon}$NH... $^{\varepsilon1}$O=C- Glu116<br>His11- $^{\varepsilon}$NH...O=C-Lys112<br>Gly10-C=O....H_2N-Lys112	H-bond<br>H-bond<br>H-bond	-5.6
2	-186.29	3	4.79	Ser3-OH....O=C-Lys65<br>Tyr1-HO....HN-Gln67<br>Tyr1-OH....O=C-Gly68	H-bond<br>H-bond<br>H-bond	-5.5
3	-187.89	9	4.13	Asn5-C=O....H$^{\varepsilon}$O-Glu135<br>Asn5-C=O....H-$^{\zeta}$N-Lys90<br>Asn5-$^{\delta}$NH.....$^{\delta}$O=C-Asn92	H-bond<br>H-bond<br>H-bond	-5.0
4	-189.61	12	3.27	Gln8- $^{\varepsilon}$NH^{1}.....O=C-Ser99<br>Gln8- $^{\varepsilon}$NH^{2}.....O=C-Ser99<br>Gly10-C=O.....H-$^{\gamma}$S-Cys69	H-bond<br>H-bond<br>H-bond	-4.7
5	-211.54	11×10^{7}	1.89	Gln8- $^{\varepsilon}$NH.....O=C- Lys112<br>Gln8-C=$^{\varepsilon}$O......H$^{\gamma}$S-Cys69	H-bond<br>H-bond	-5.1
6	-218.19	12×10^{9}	Ref (0.0)	Tyr2-OH....O=C-Asn137<br>Asn5- $^{\varepsilon}$NH^{1}....$^{\gamma}$O-Thr79<br>Asn5- $^{\varepsilon}$NH^{2}....$^{\gamma}$O-Thr79<br>Glu4-C=O..........H- $^{\varepsilon}$N-Asn92	H-bond<br>H-bond<br>H-bond<br>H-bond	-5.0

Peptide synthesis and analysis

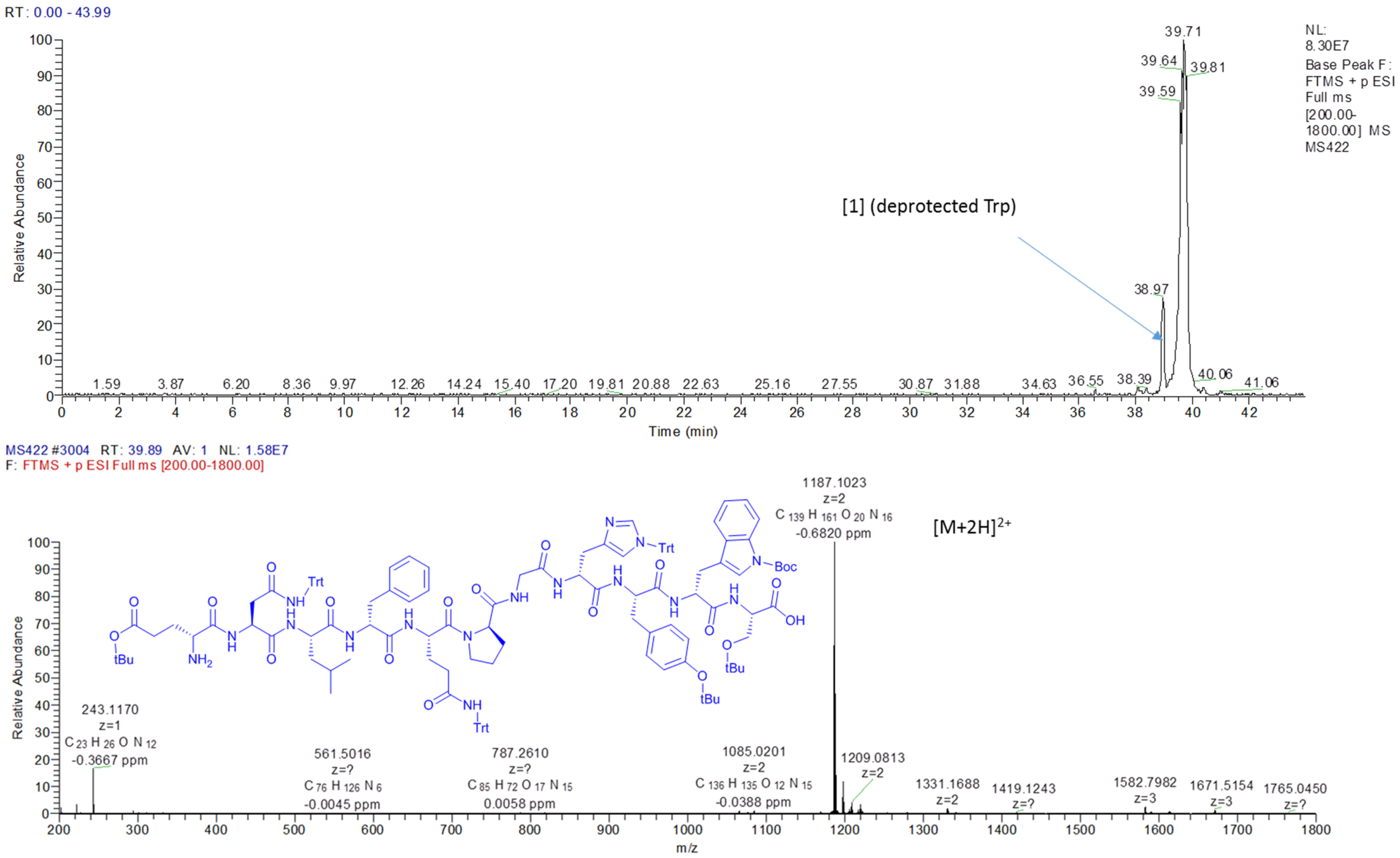

Figure S2. 1: LC-MS chromatogram of fully protected linear precursor peptide of [1].

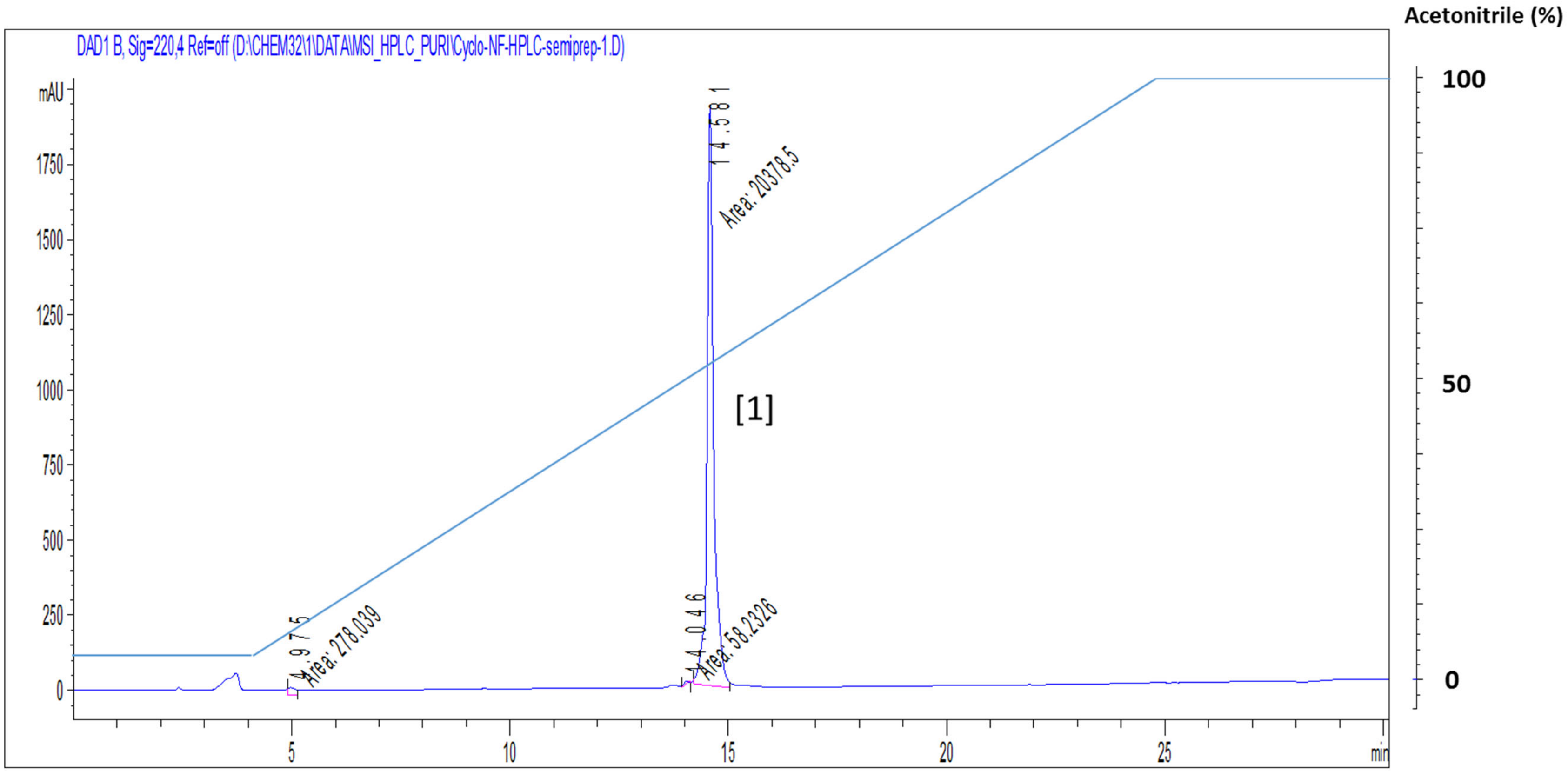

#	Time	Area	Height	Width	Area%	Symmetry
1	4.975	278	23.7	0.1951	1.342	0.513
2	14.046	58.2	8.7	0.1121	0.281	5.567
3	14.581	20378.5	1934.9	0.1755	98.377	0.828

Figure S2. 2: HPLC trace of purified WP9 peptidomimetic [1].
Purity was calculated by peak area % shown in the table using Agilent OpenLab CDS software. UV wave length of 220 nm is shown.

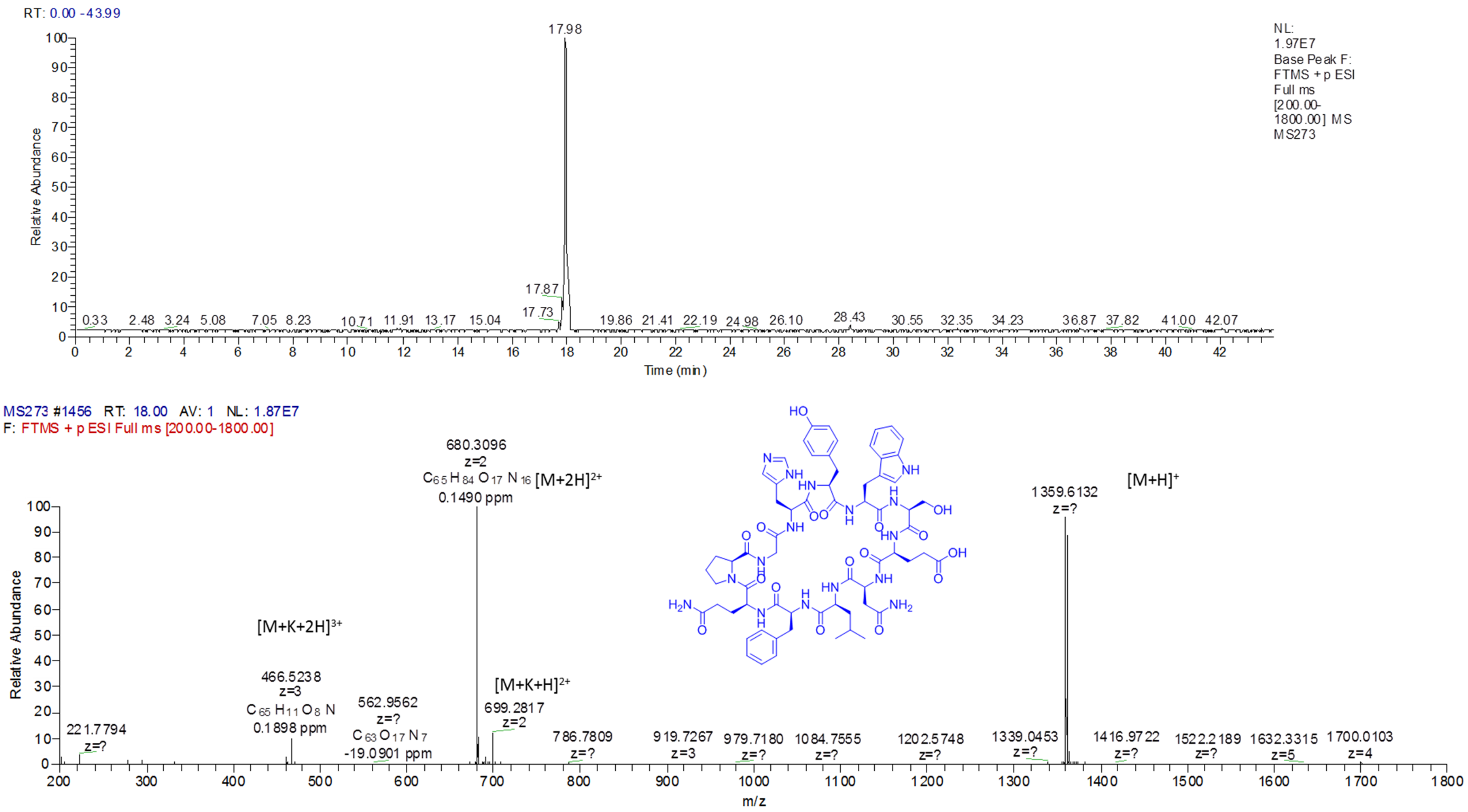

Figure S2. 3: LC-MS chromatogram of purified WP9 peptidomimetic [1].

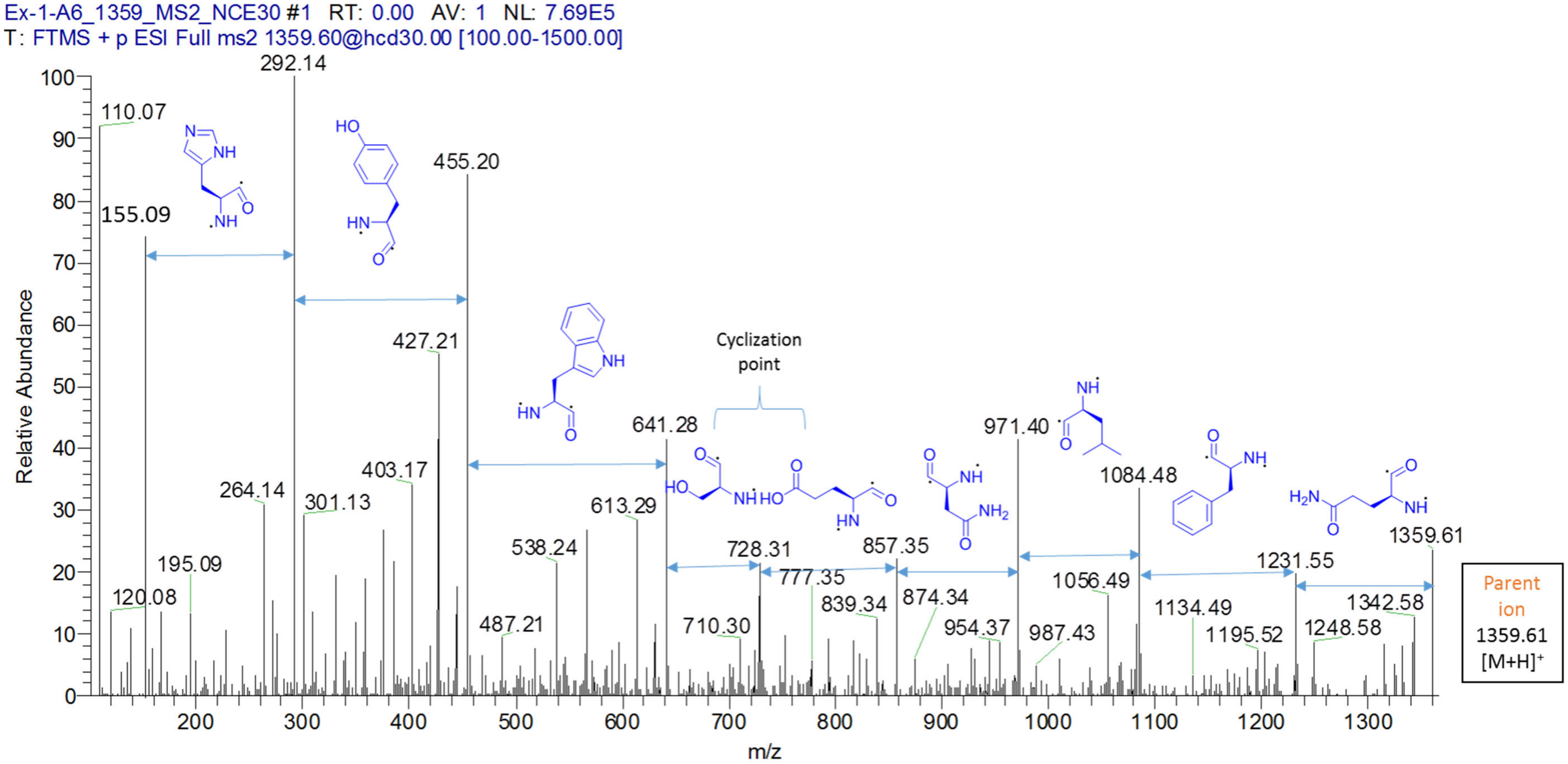

Figure S2. 4: MS/MS fragmentation spectrum of WP9 peptidomimetic [1].

Assignments of NMR data to the structure of [1]

Both One 1D and 2D NMR experiments including TOCSY, COSY, HSQC, and HMBC of **[1]** in methanol-d_3 was carried out. These were done to identify the amino acid residues of **[1]** and their order in the structure. A summary of important 2D NMR assignments of **[1]** are illustrated in Figure S3.1-2. Details of 2D NMR analysis of are shown in Table S3.1 and Figures S3.3-41.

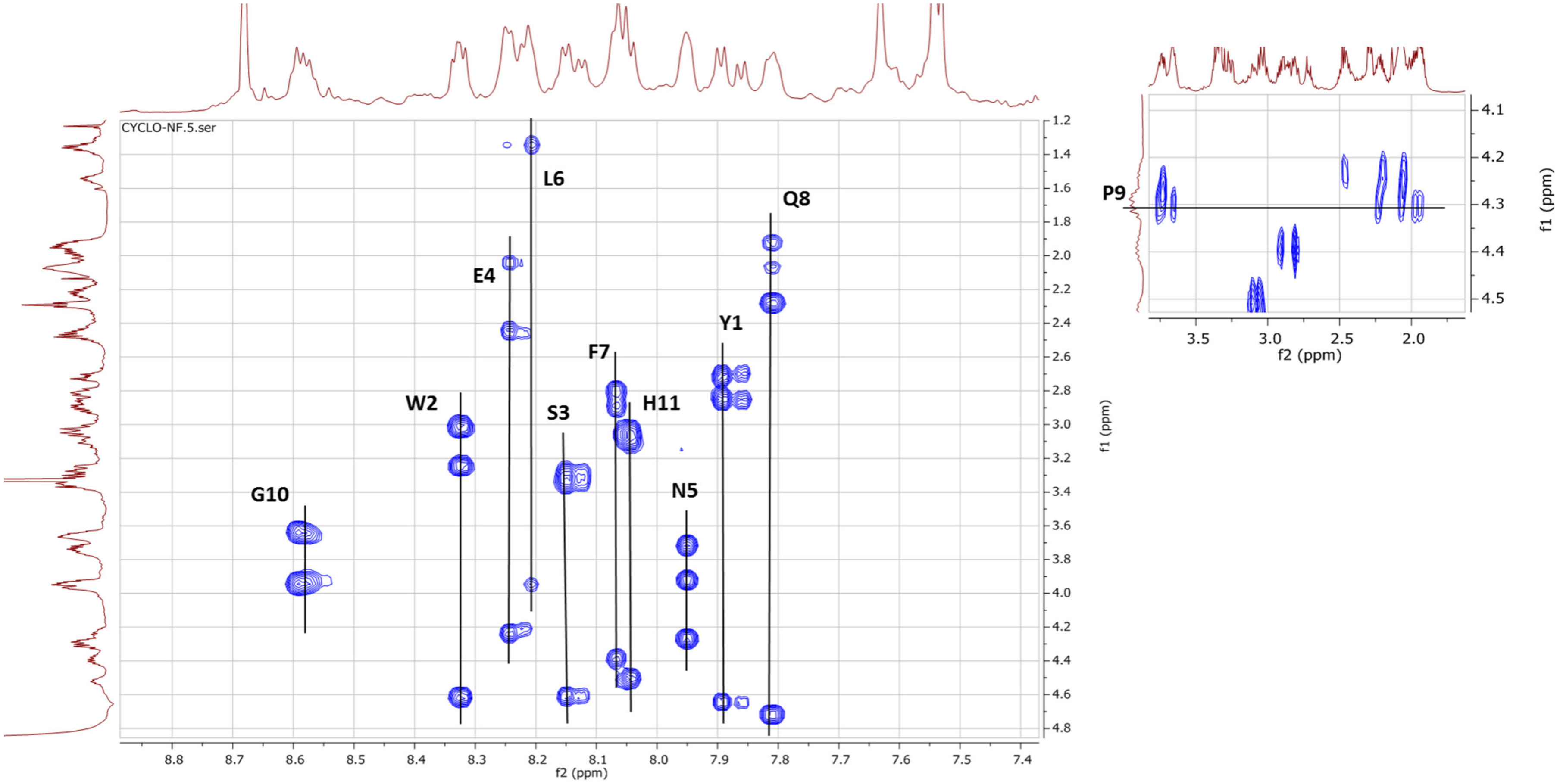

Figure S3. 1: TOCSY spectrum of WP9 peptidomimetic [1] showing its different residues.

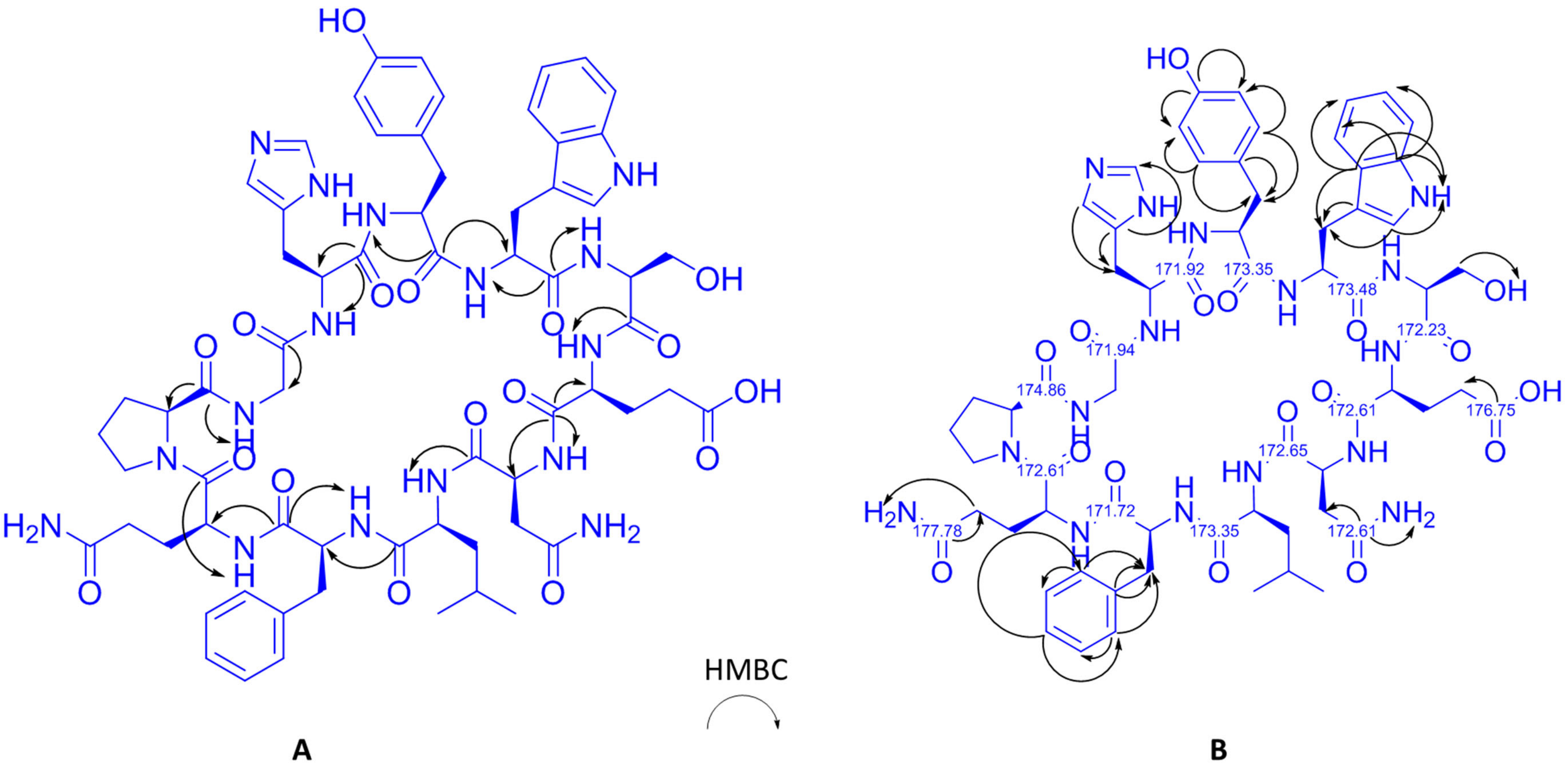

Figure S3. 2: Key HMBC correlations of WP9 peptidomimetic [1].
(A) Main chain HMBC correlations of **[1]**. **(B)** Side chain HMBC correlations of **[1]** with ^{13}C chemical shift values reported for all carbonyl groups.

Table S3.1: WP9 peptidomimetic [1] NMR assignments.
The sample was prepared in methanol-*d3* and run at 10 ^{0}C on 600 MHz NMR spectrometer. ('or'' represents protons attached to corresponding carbon atoms).

residue	Position	TOCSY	^{1}Hδ	^{13}C δ	HMBC
G10	N1	NH	8.59		2, 1'<br>2, 1''
	1	$^{\alpha}CH_2$	3.63	43.99	
			3.97		
	2	C=O		171.94	
W2	N2	NH	8.32		5,N2,N5
	3	$^{\alpha}$CH	4.64	51.81	C3, 4'', C2,C3a<br>C2, N2$^{'indole}$, C3a, C7a<br>C7a, C4', C6'<br>C3a, C5', C7
	4	$^{\beta}CH_2$	3.03	27.58	
			3.27		
	5	C=O		173.48	
E4	N3	NH	8.24		9, N8, 26'<br>10, 8'
	6	$^{\alpha}$CH	4.25	55.03	
	7	$^{\beta}CH_2$	2.04	26.70	
			2.12<br>(HSQC)		
	8	$^{\gamma}CH_2$	2.44	31.03	
			2.44		
	9	C=O		172.61	
	10	$^{\delta}$COOH		176.75	
L6	N4	NH	8.22		16, 20'
	11	$^{\alpha}$CH	3.95	54.82	

	12	$^{\beta}CH_2$	1.33	41.20	
			1.33		
	13	$^{\gamma}CH$	1.54 (COSY)		
	14	$^{\delta1}CH_3$	0.80	23.00	
	15	$^{\delta2}CH_3$	0.71	22.00	
	16	C=O		173.35	
S3	N5	NH	8.15		19, N3<br>18, 18^{-OH}
	17	$^{\alpha}CH$	4.62	56.43	
	18	$^{\beta}CH_2$	3.35	27.60	
			3.35		
		-OH	4.60 (HMBC)		
	19	C=O		172.23	
F7	N6	NH	8.06		22, N6, 33’<br><br>C1, 21’,21’’<br>C2/C6, 21’, C1<br>C2/C6, C4’, C3/C5’
	20	$^{\alpha}CH$	4.39	57.46	
	21	$^{\beta}CH_2$	2.80	37.43	
			2.88		
	22	C=O	171.72		
H11	N7	NH	8.04		25, N7, 23’<br><br>C1, 24’, C3’<br>C5, 24’
	23	$^{\alpha}CH$	4.51	53.95	
	24	$^{\beta}CH_2$	3.09	27.74	
			3.04 (HSQC)		
	25	C=O		171.92	
N5	N8	NH	7.95		28, N4<br>29, N8, 29^{-NH2}

	26	$^{\alpha}$CH	4.27	62.52	
	27	$^{\beta}CH_2$	3.74	48.66 *	
			3.95		
	28	C=O		172.65	
		NH_2	8.22		
	29	$^{\gamma}$C=O		171.61	
Y1	N9	NH	7.89		32, N9, 3’<br><br>C1, 31’, C2/C6<br>C2/C6, C3’/C5’, C4
	30	$^{\alpha}$CH	4.67	51.74	
	31	$^{\beta}CH_2$	2.70	37.78	
			2.85		
	32	C=O		173.35	
Q8	N10	NH	7.81		36, N10,<br>37, 35’<br>35, 35^{-NH2}
	33	$^{\alpha}$CH	4.72	51.63	
	34	$^{\beta}CH_2$	1.91	30.26	
			2.06		
	35	$^{\gamma}CH_2$	2.27	32.16	
			2.27		
	36	C=O		172.61	
		NH_2	6.84		
	37	$^{\delta}$C=O		177.78	
P9	38	$^{\alpha}$CH	4.29	62.83	42, N1, 39’
	39	$^{\beta}CH_2$	2.22	30.27	
	40	$^{\gamma}CH_2$	1.97	26.00	

			1.97		
	41	$^{\delta}CH_2$	3.64	48.74	
			3.64		
	42	C=O		174.86	

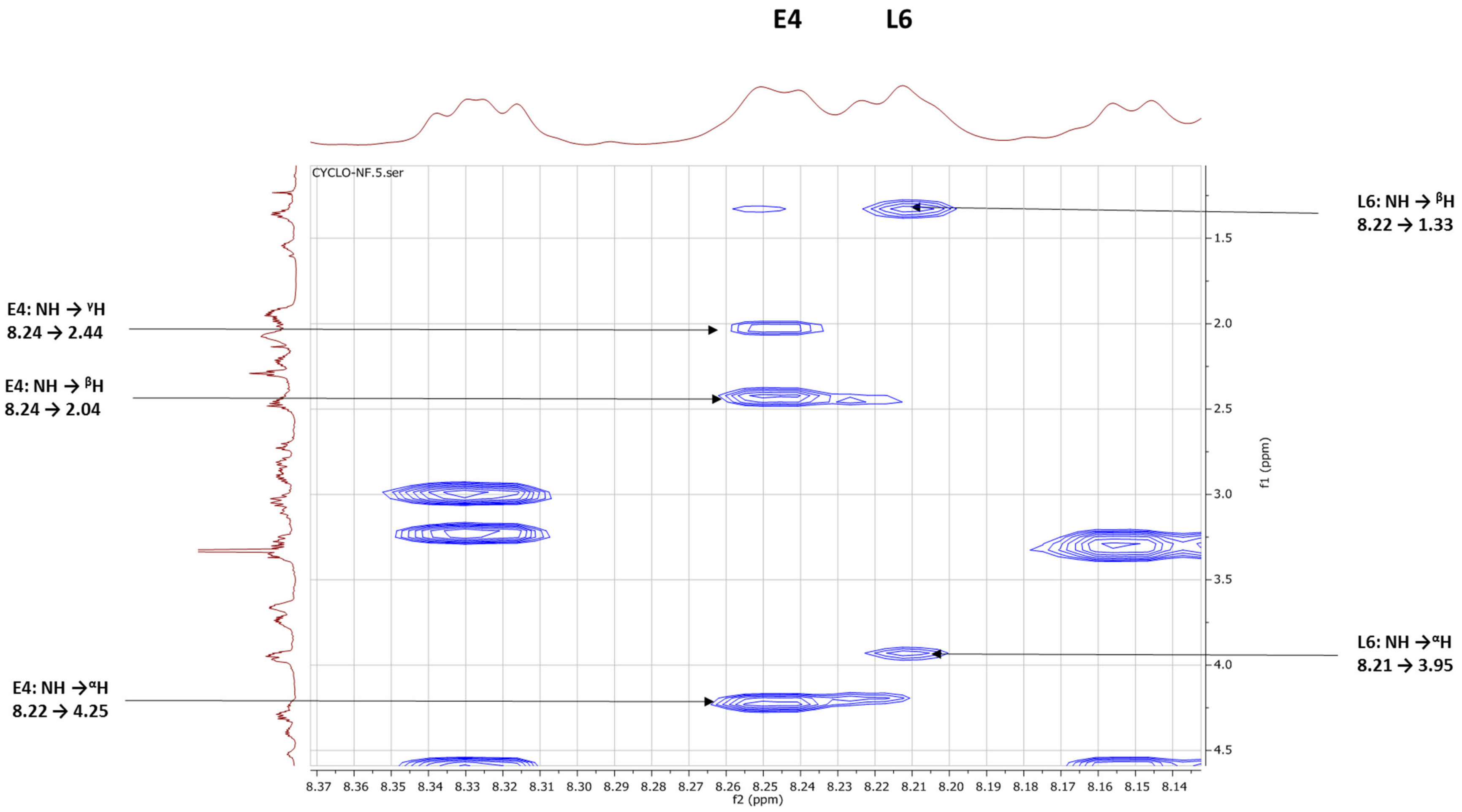

Figure S3. 3: TOCSY spectrum of [1] showing residues E4 and L6.
Mixing time of 80 ms was used.

[1]
Y W S E N L F Q P G H

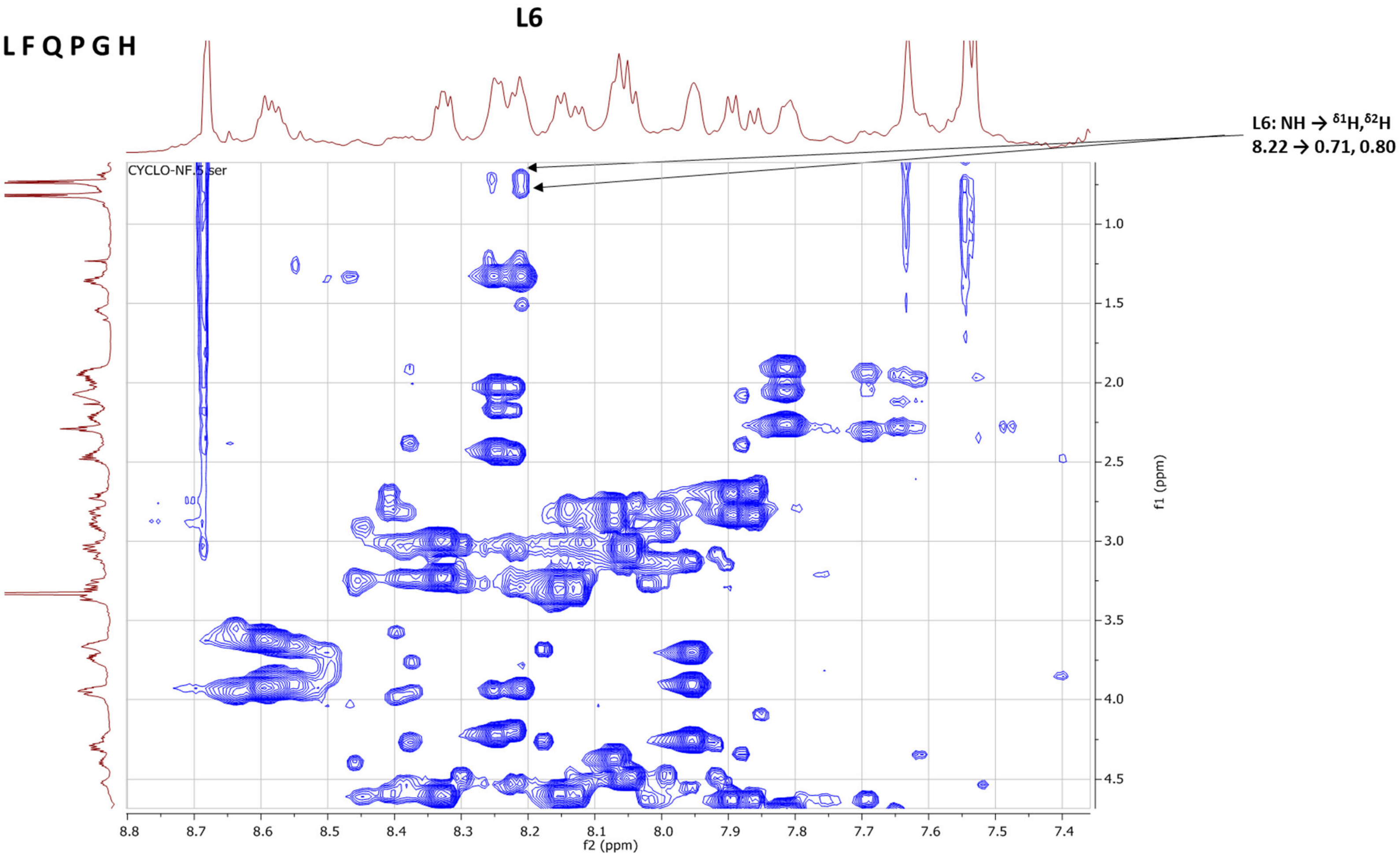

Figure S3. 4: TOCSY spectrum of [1] showing residues L6.
Mixing time of 80 ms was used.

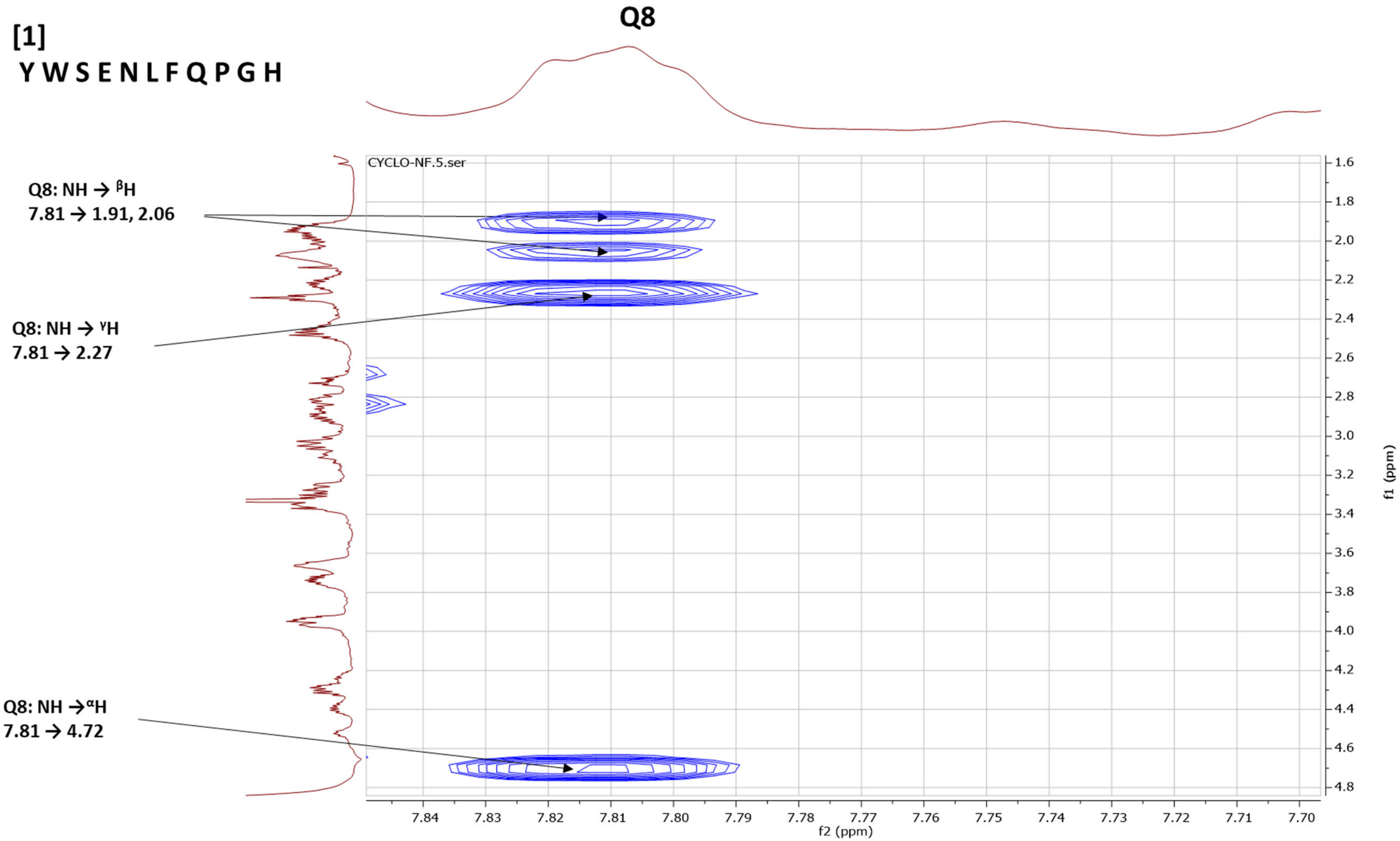

Figure S3. 5: TOCSY spectrum of [1] showing residues Q8.
Mixing time of 80 ms was used.

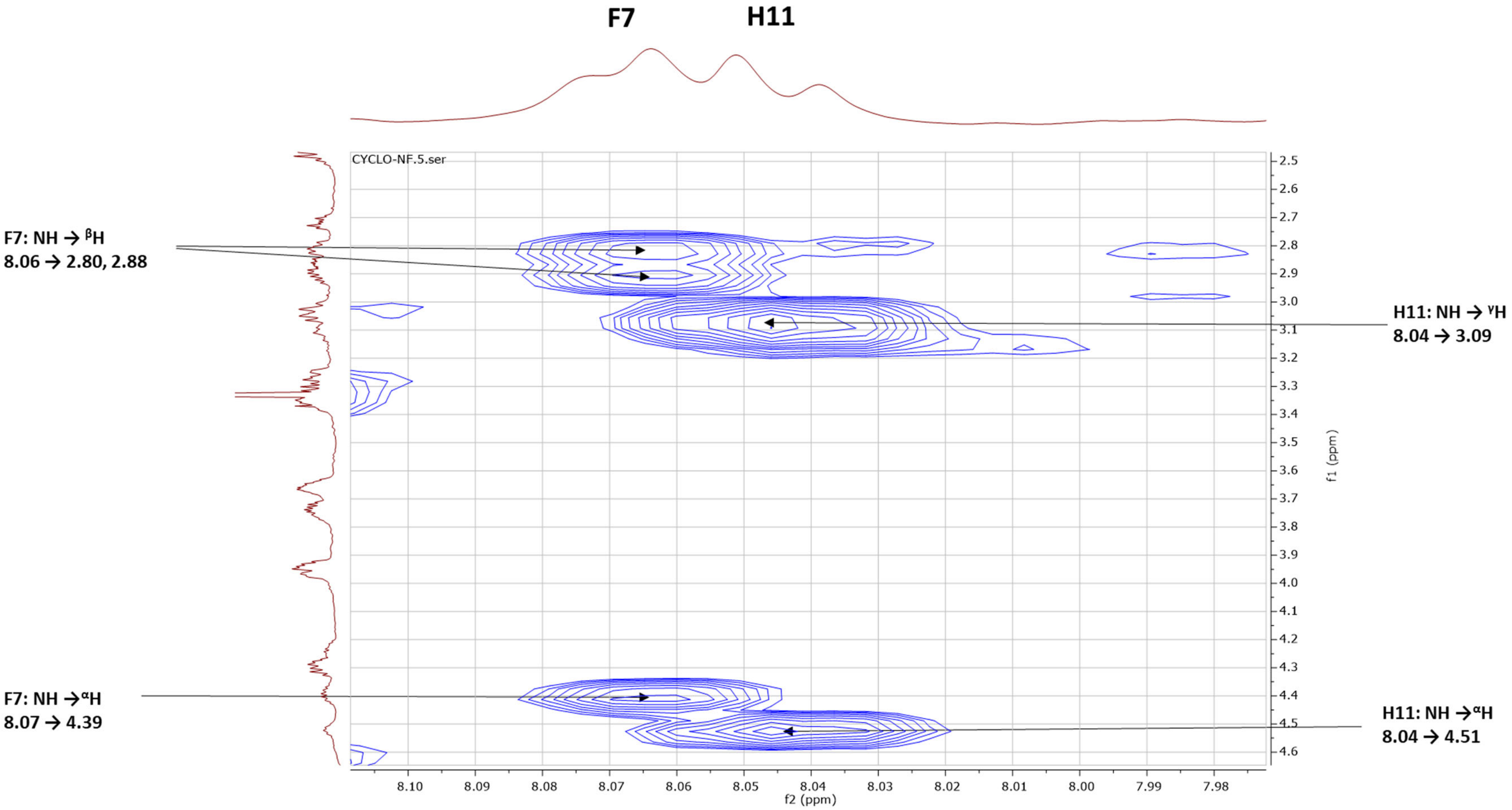

Figure S3. 6: TOCSY spectrum of [1] showing residues F7 and H11.
Mixing time of 80 ms was used.

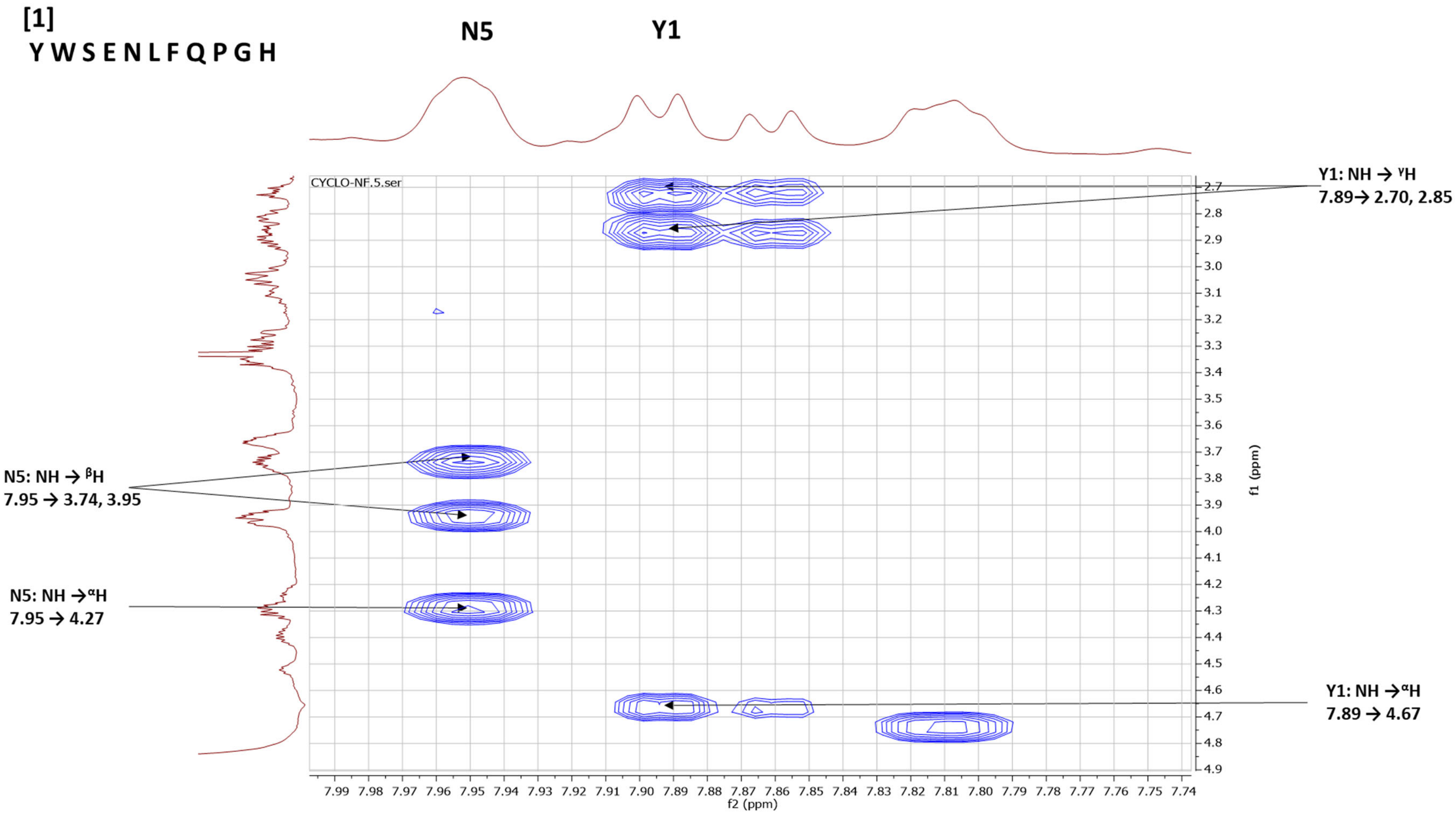

Figure S3. 7: TOCSY spectrum of [1] showing residues Y1 and N5.
Mixing time of 80 ms was used.

[1]
Y W S E N L F Q P G H

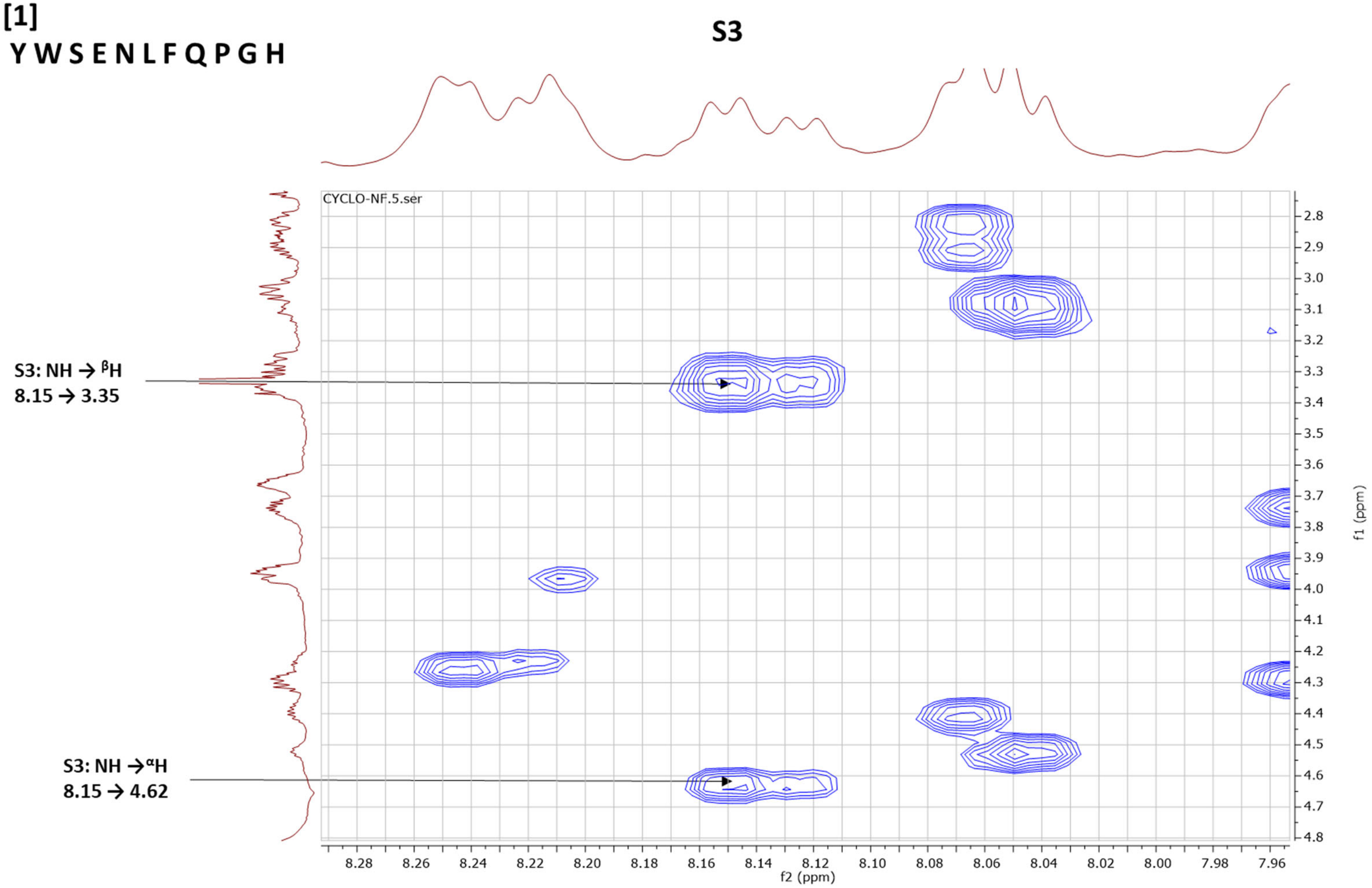

Figure S3. 8: TOCSY spectrum of [1] showing residues S3.
Mixing time of 80 ms was used.

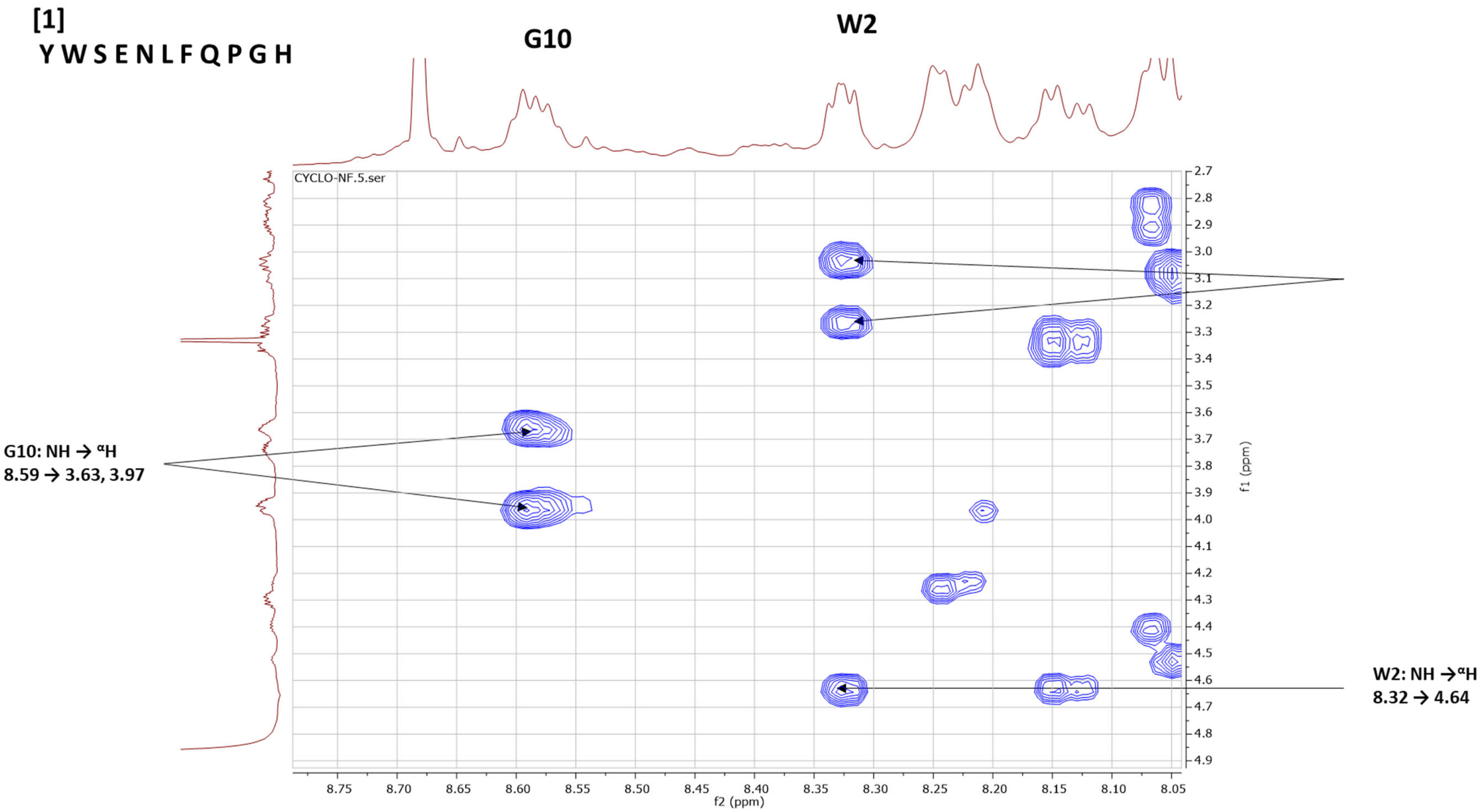

Figure S3. 9: TOCSY spectrum of [1] showing residues W2 and G10.
Mixing time of 80 ms was used.

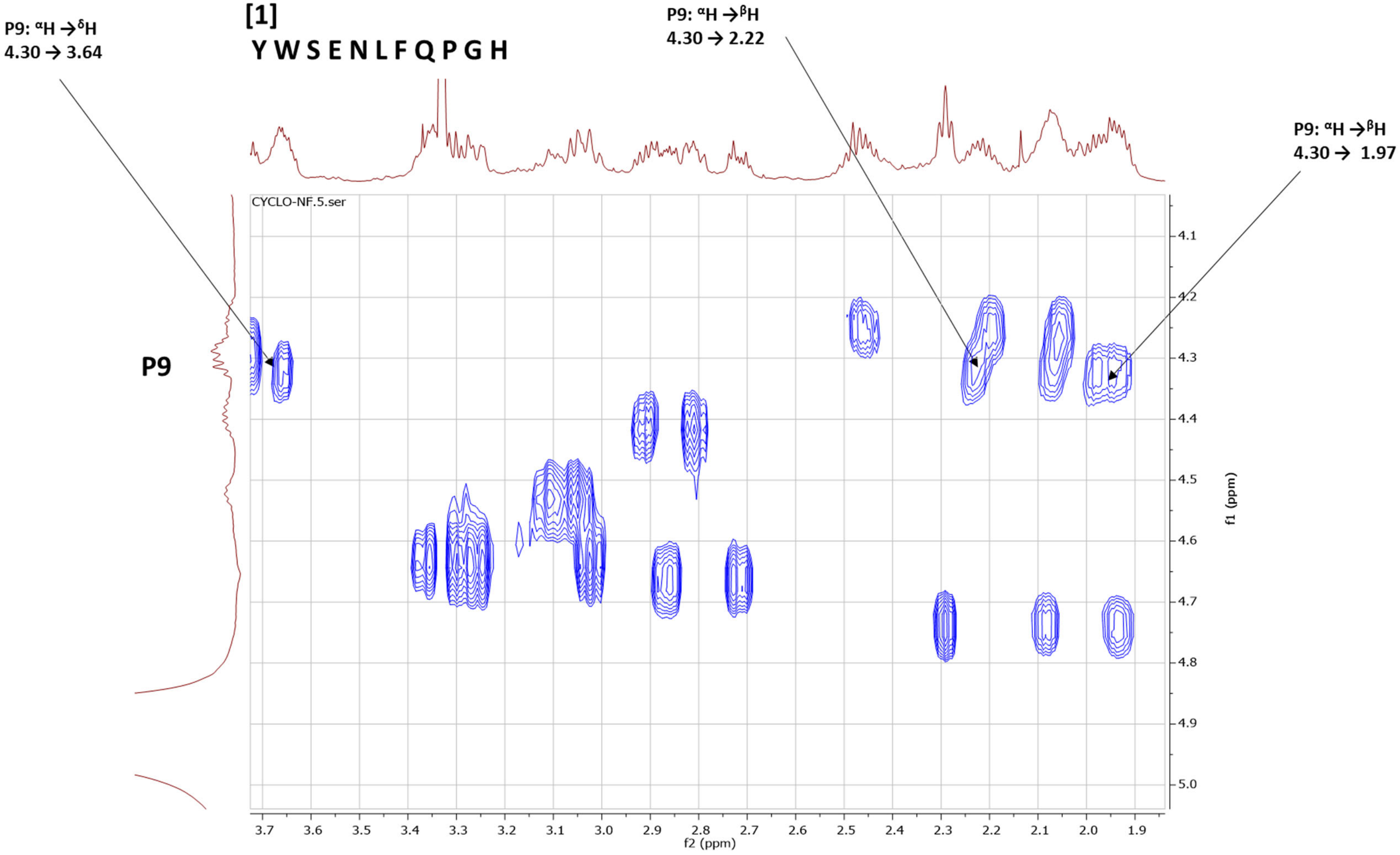

Figure S3. 10: TOCSY spectrum of [1] showing residues P9.
Mixing time of 80 ms was used.

[1]
Y W S E N L F Q P G H

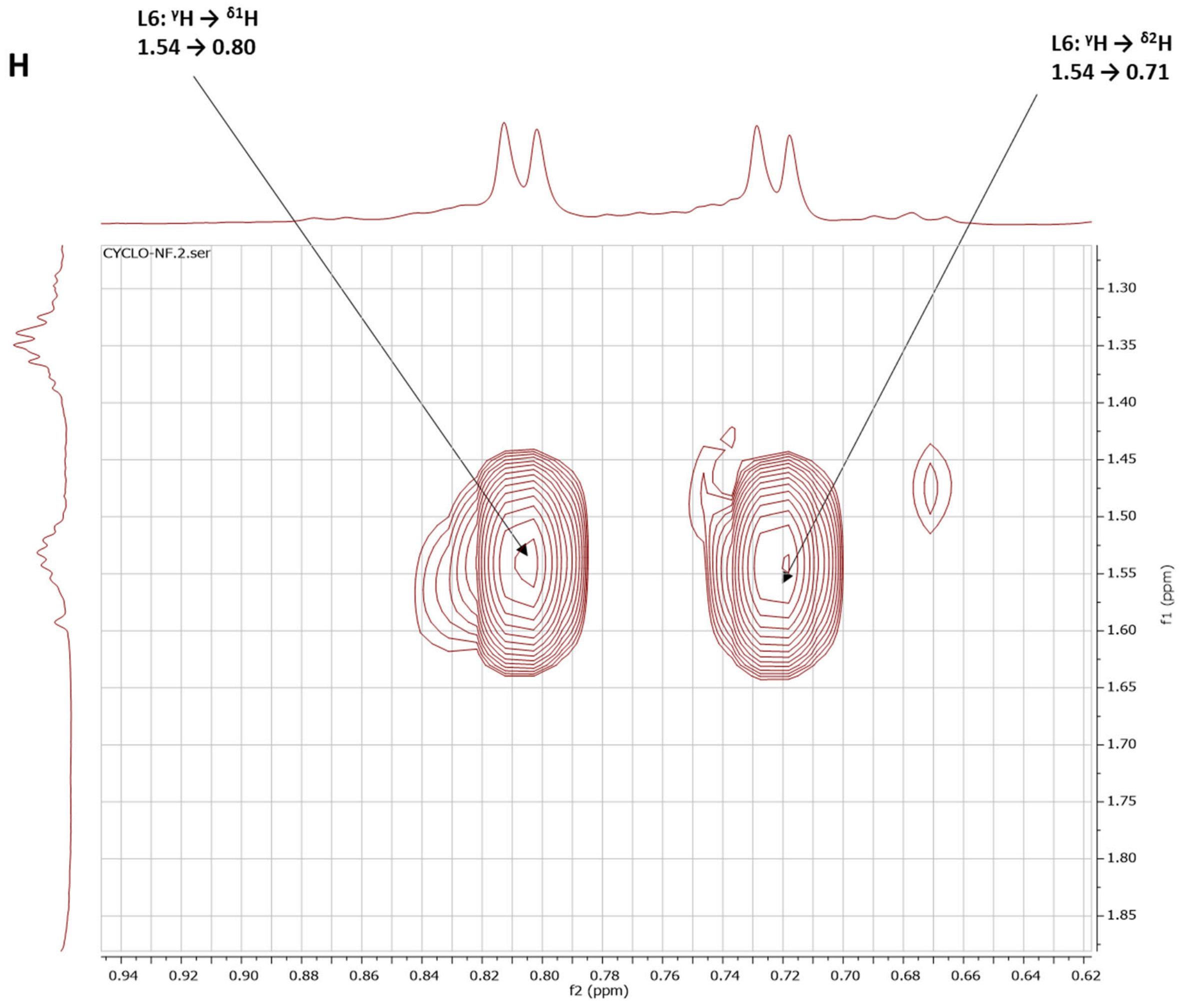

Figure S3. 11: COSY spectrum of [1] showing residues L6 side chain.

[1]

Y W S E N L F Q P G H

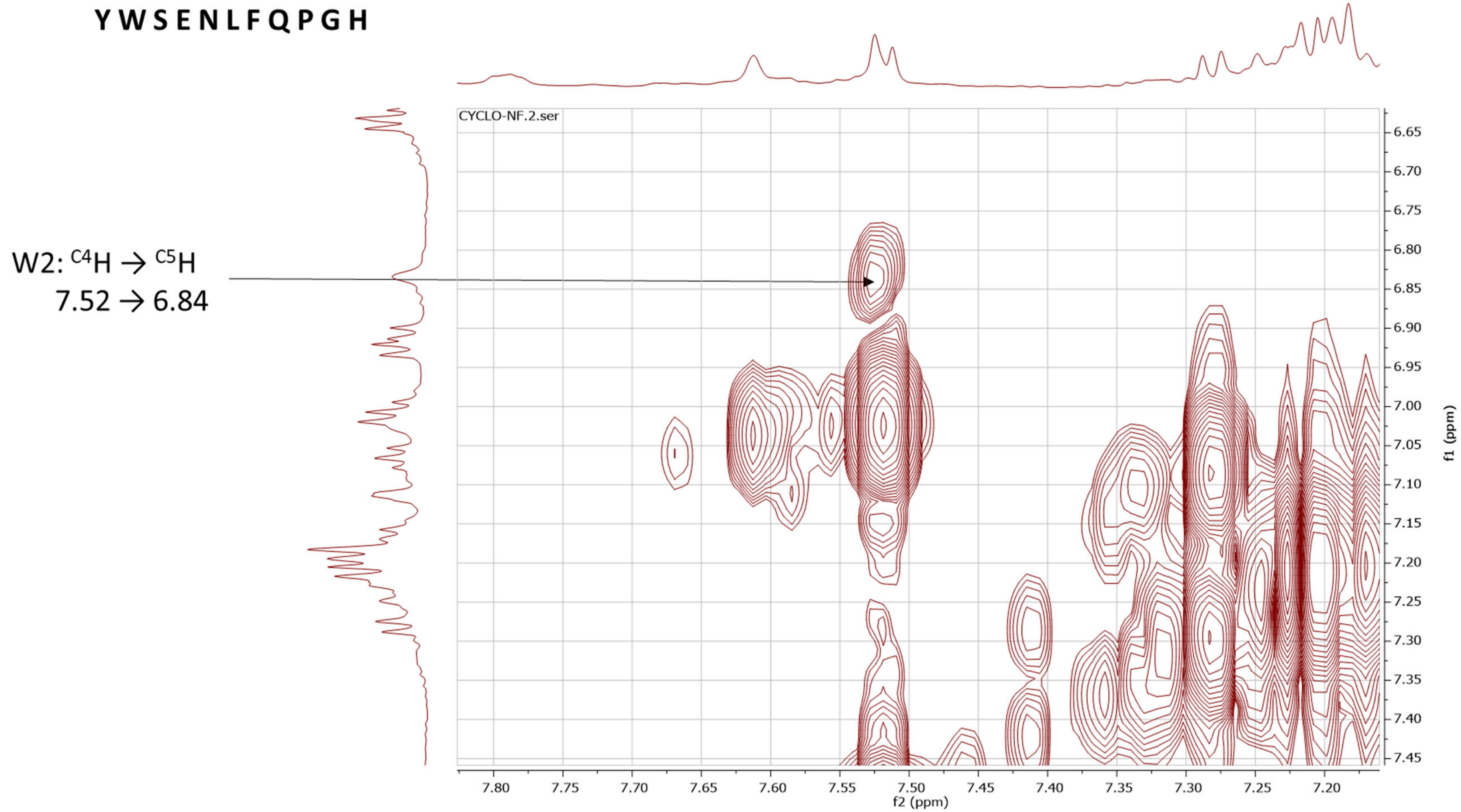

Figure S3. 12: COSY spectrum of [1] showing residues W2 side chain.

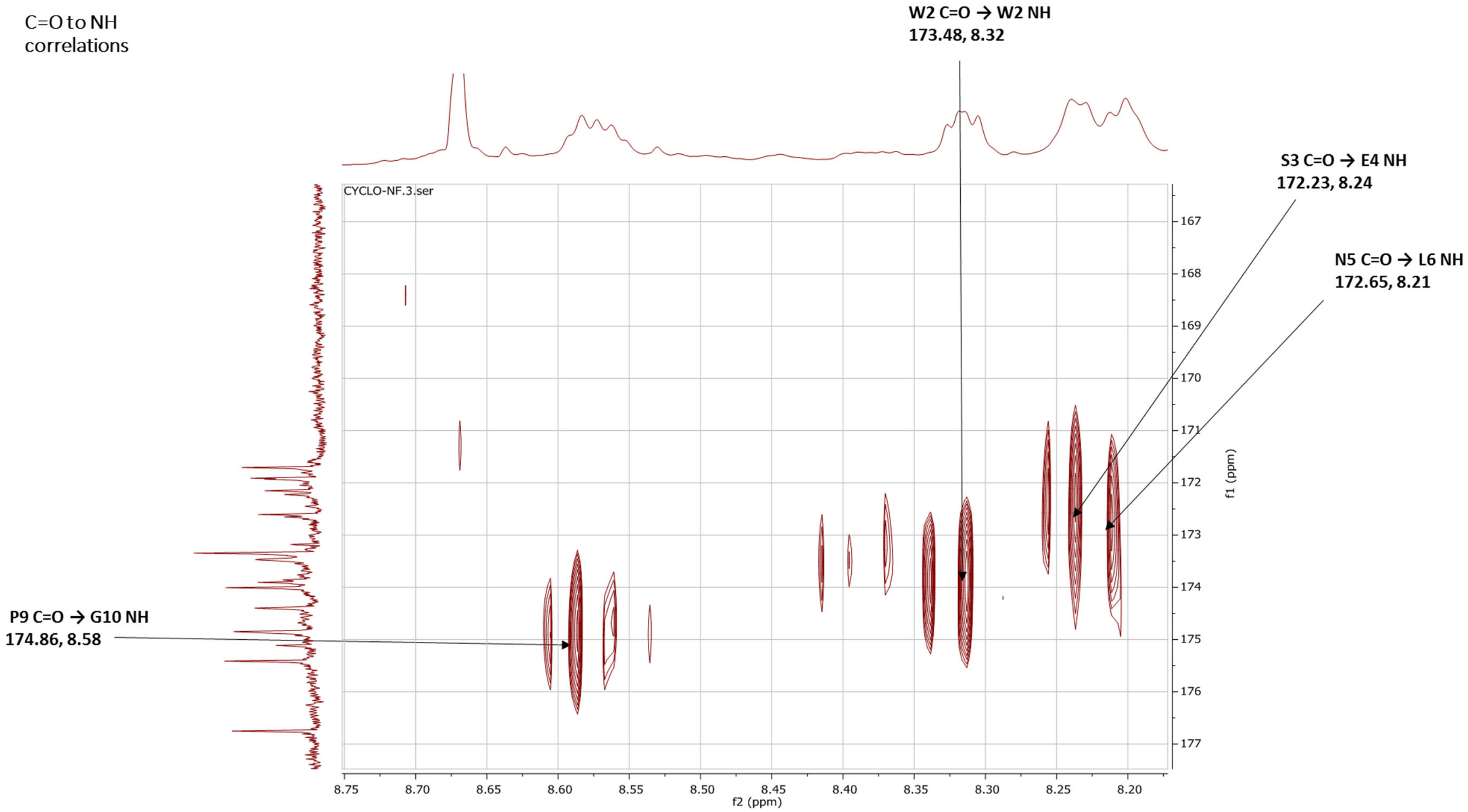

Figure S3. 13: HMBC correlations of [1] showing residue W2, G10, E4, and L6 (main chain NH to carbonyl groups).

C=O to NH
correlations

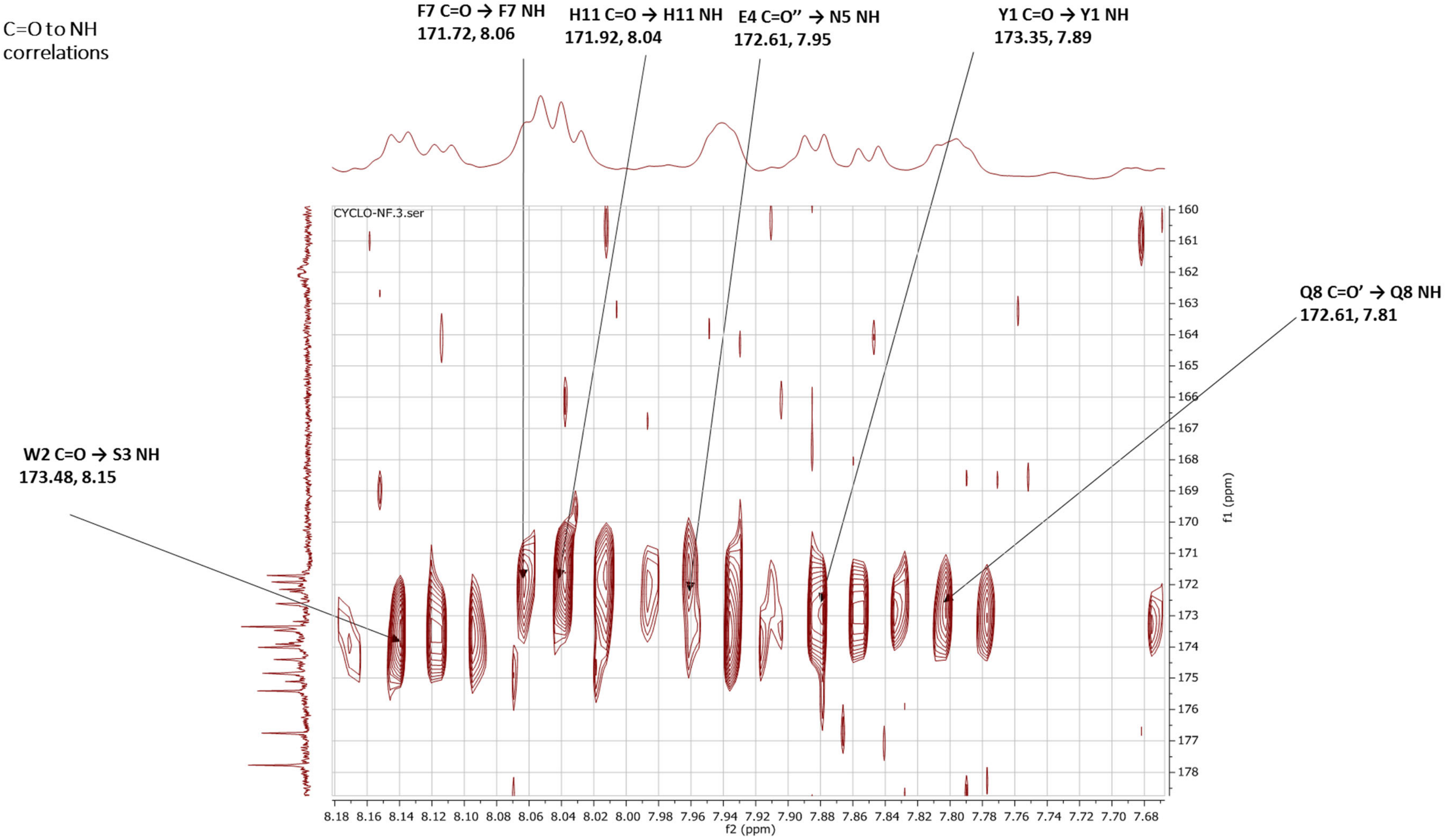

Figure S3. 14: HMBC correlations of [1] showing residue F7, S3, H11, N5, Y1, and Q8 (main chain NH to carbonyl groups).

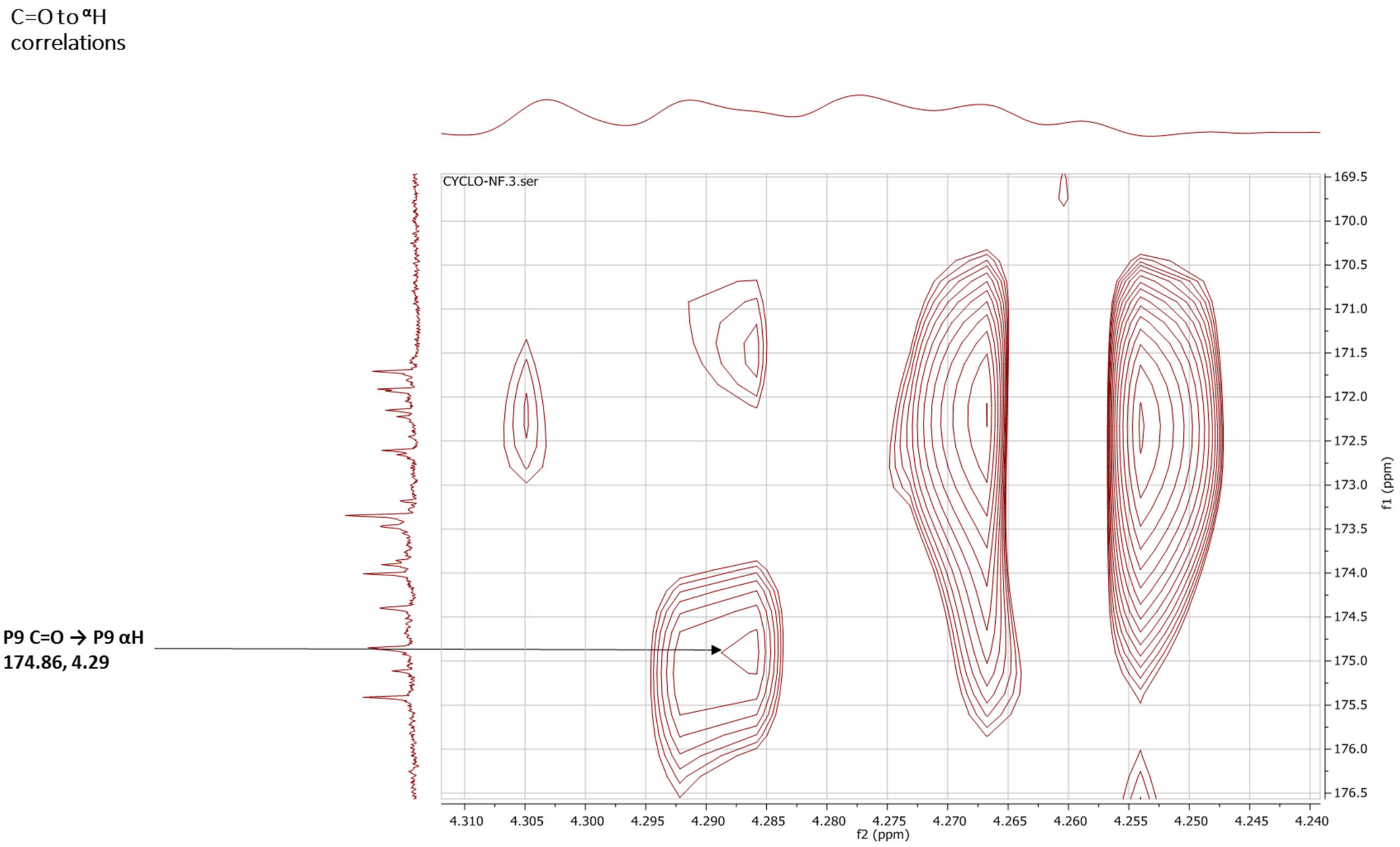

Figure S3. 15: HMBC correlation of [1] showing residue P9 (main chain NH to carbonyl group).

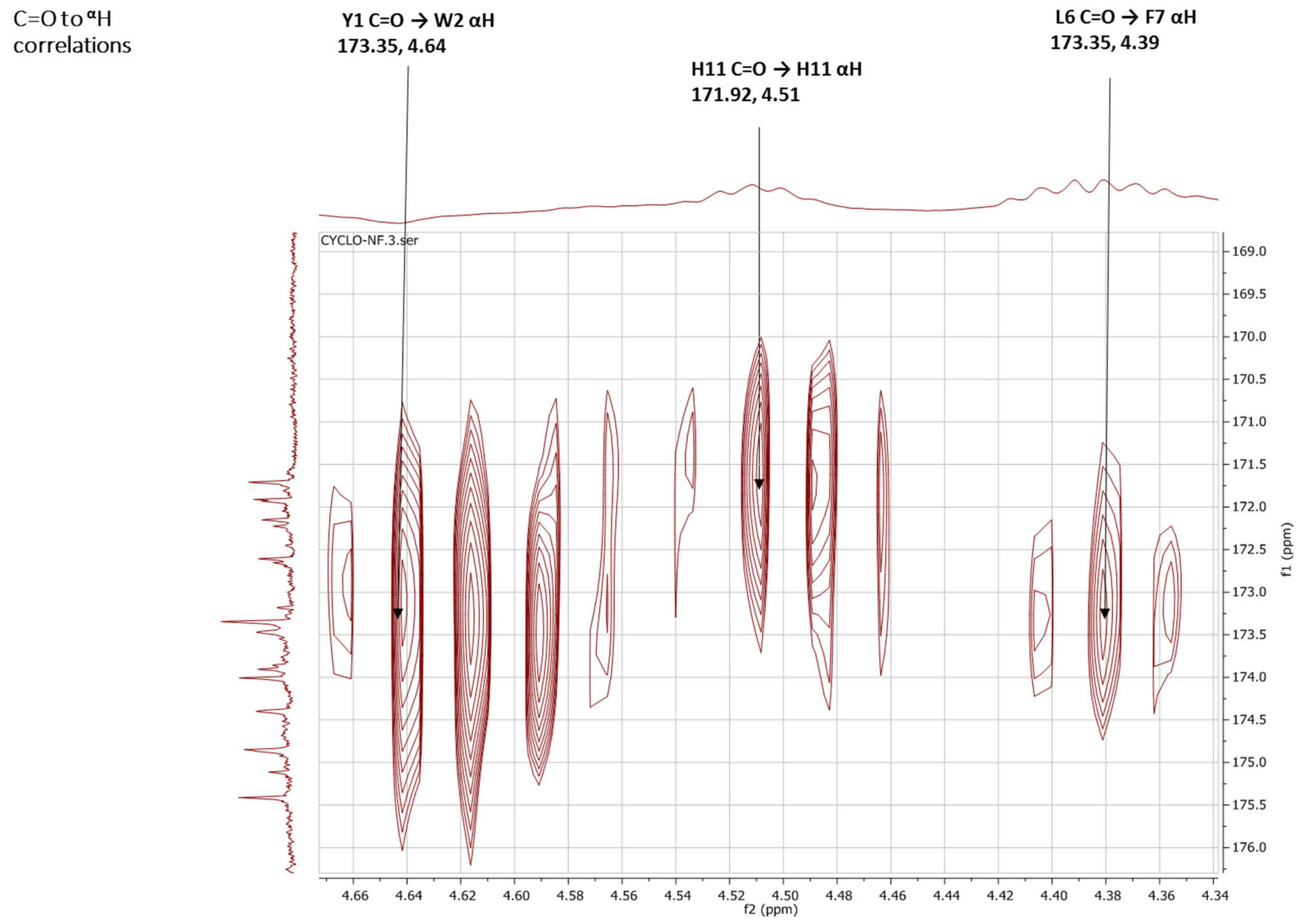

Figure S3. 16: HMBC correlations of [1] showing residue W2, H11 and F7 (main chain αH to carbonyl groups).

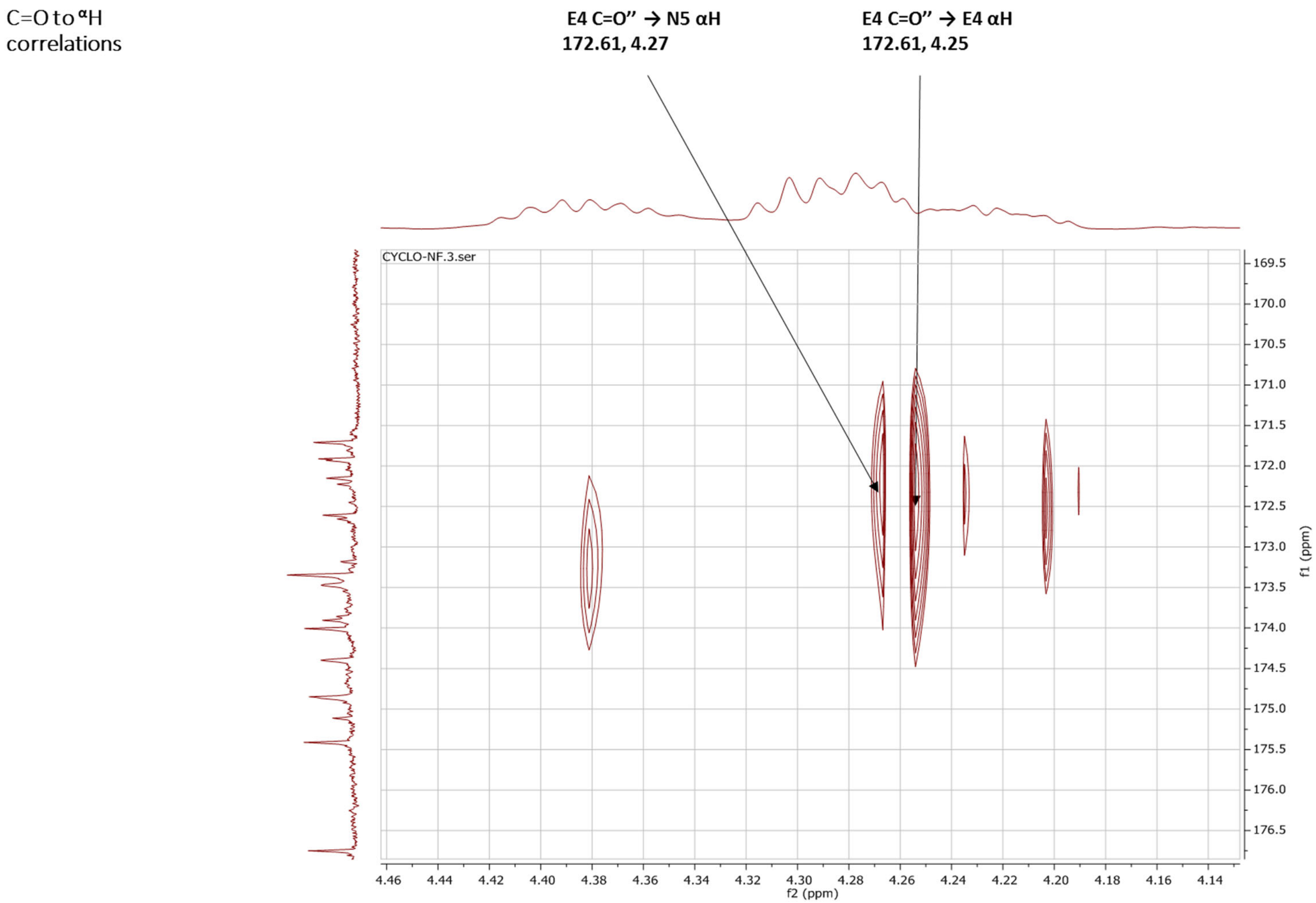

Figure S3. 17: HMBC correlations of [1] showing residue N5 and E4 (main chain αH to carbonyl groups).

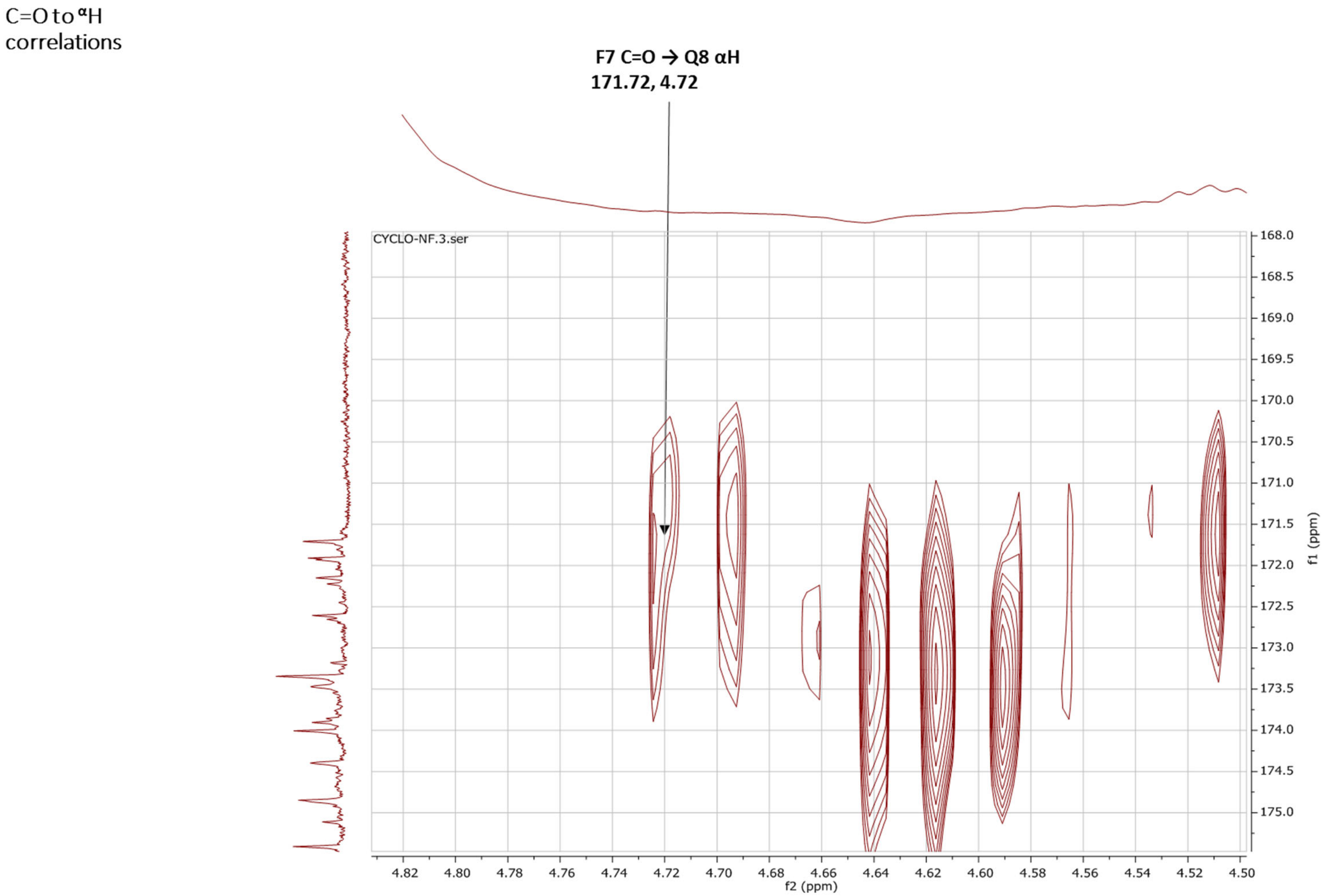

Figure S3. 18: HMBC correlation of [1] showing residue Q8 (main chain αH to carbonyl group).

C=O to αH
correlations

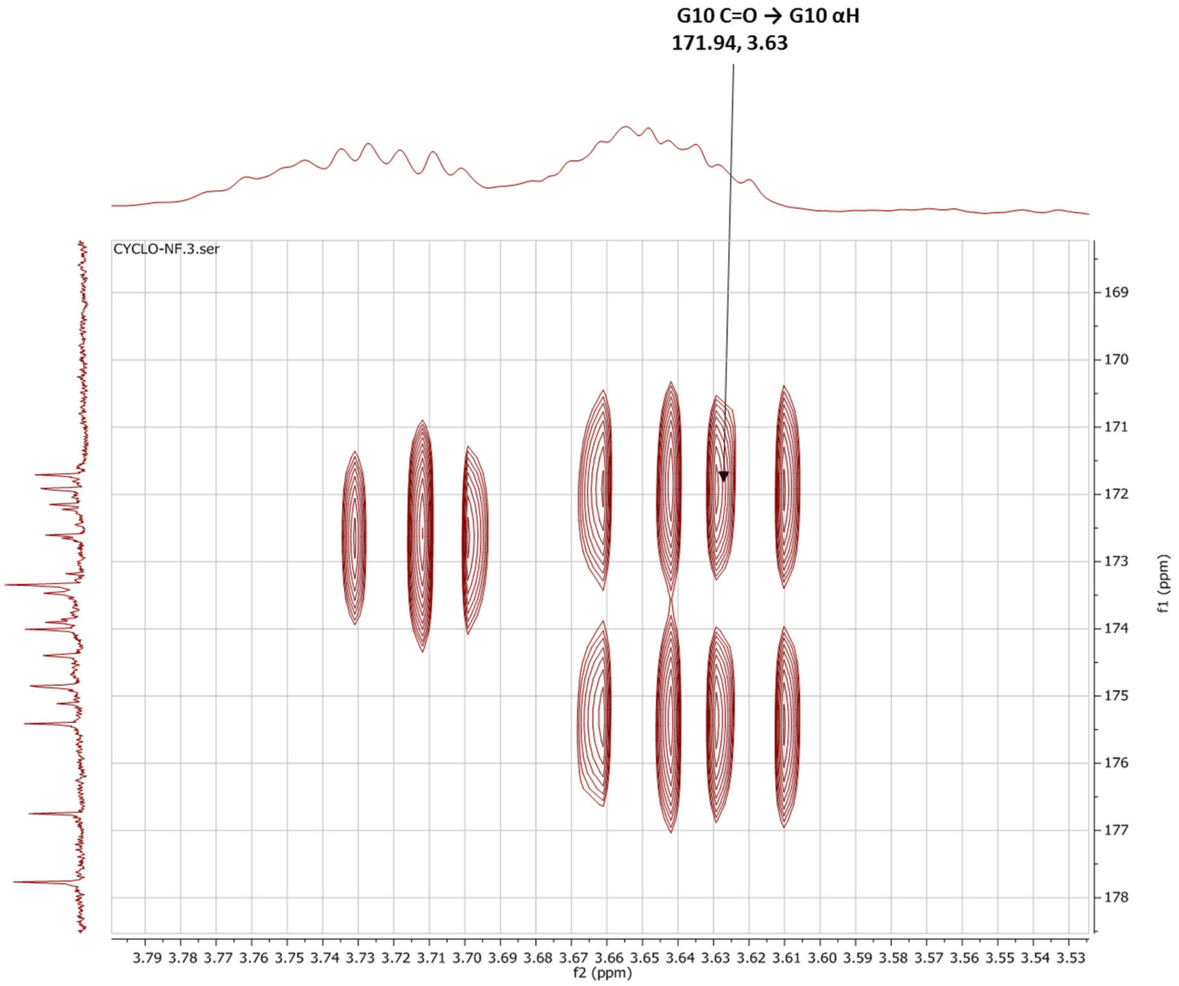

Figure S3. 19: HMBC correlation of [1] showing residue G10 (main chain αH to carbonyl group).

C=O to αH correlations

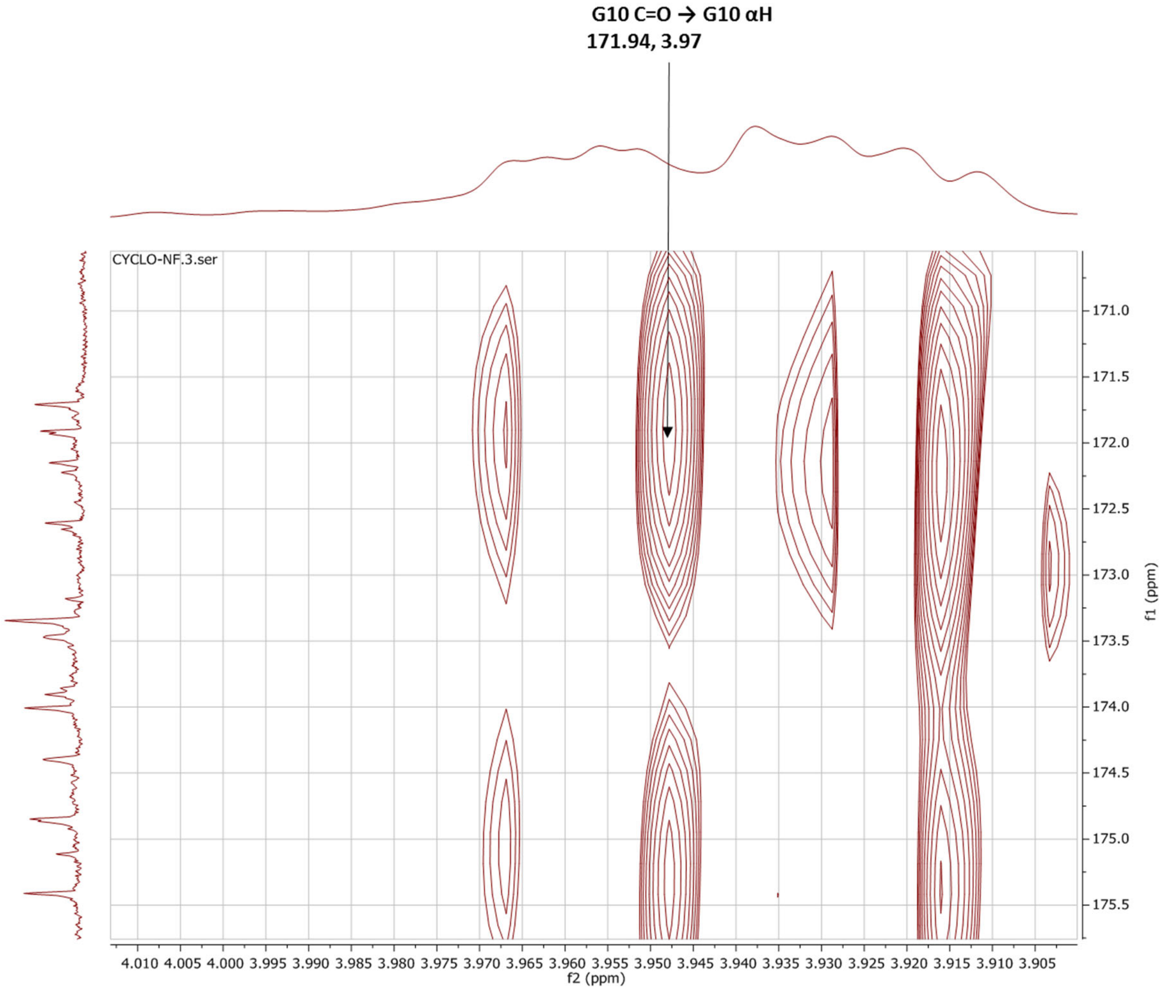

Figure S3. 20: HMBC correlation of [1] showing residue G10 (main chain αH to carbonyl group).

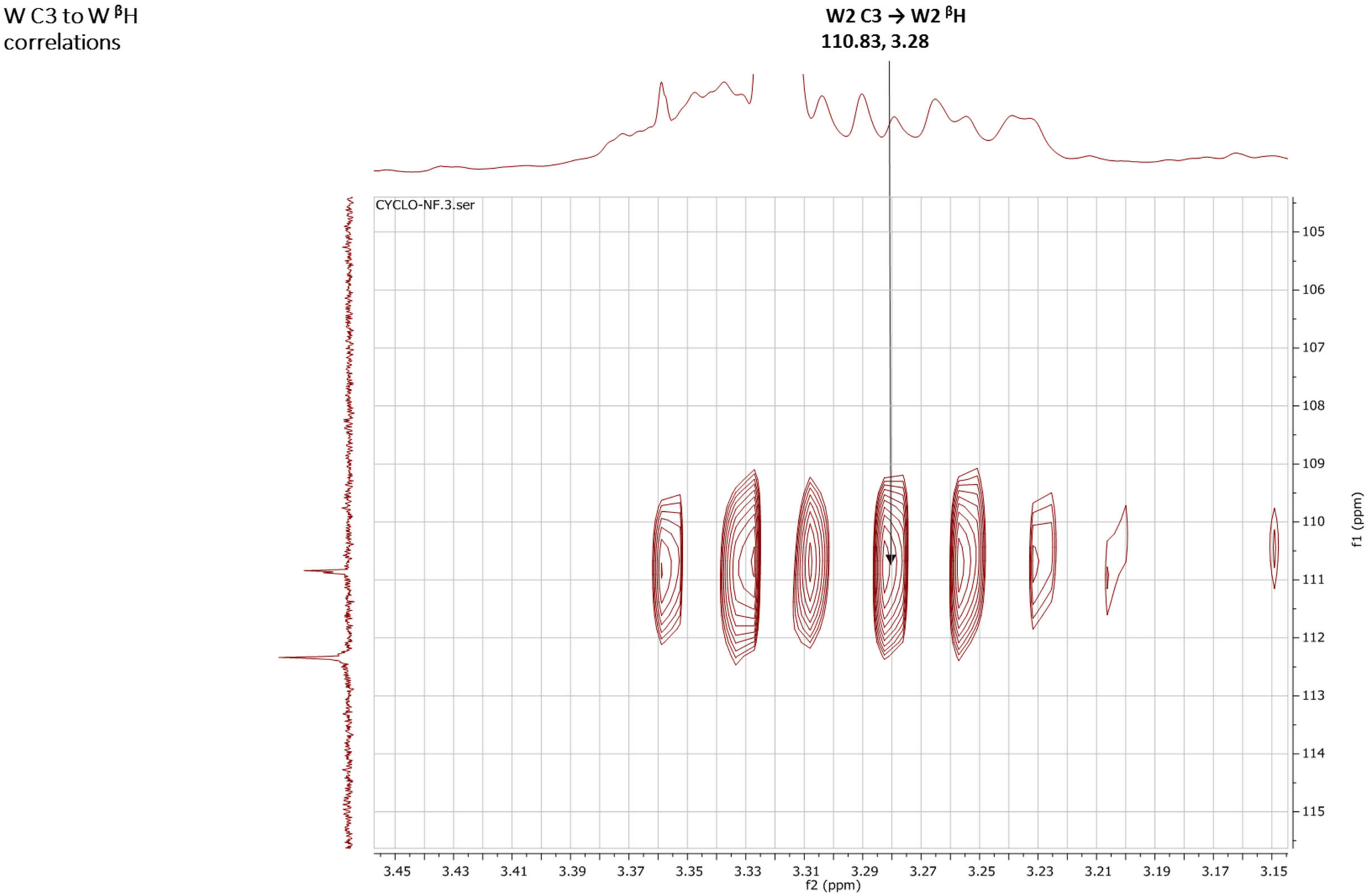

Figure S3. 21: HMBC correlation of [1] showing residue W2 side chain (1).

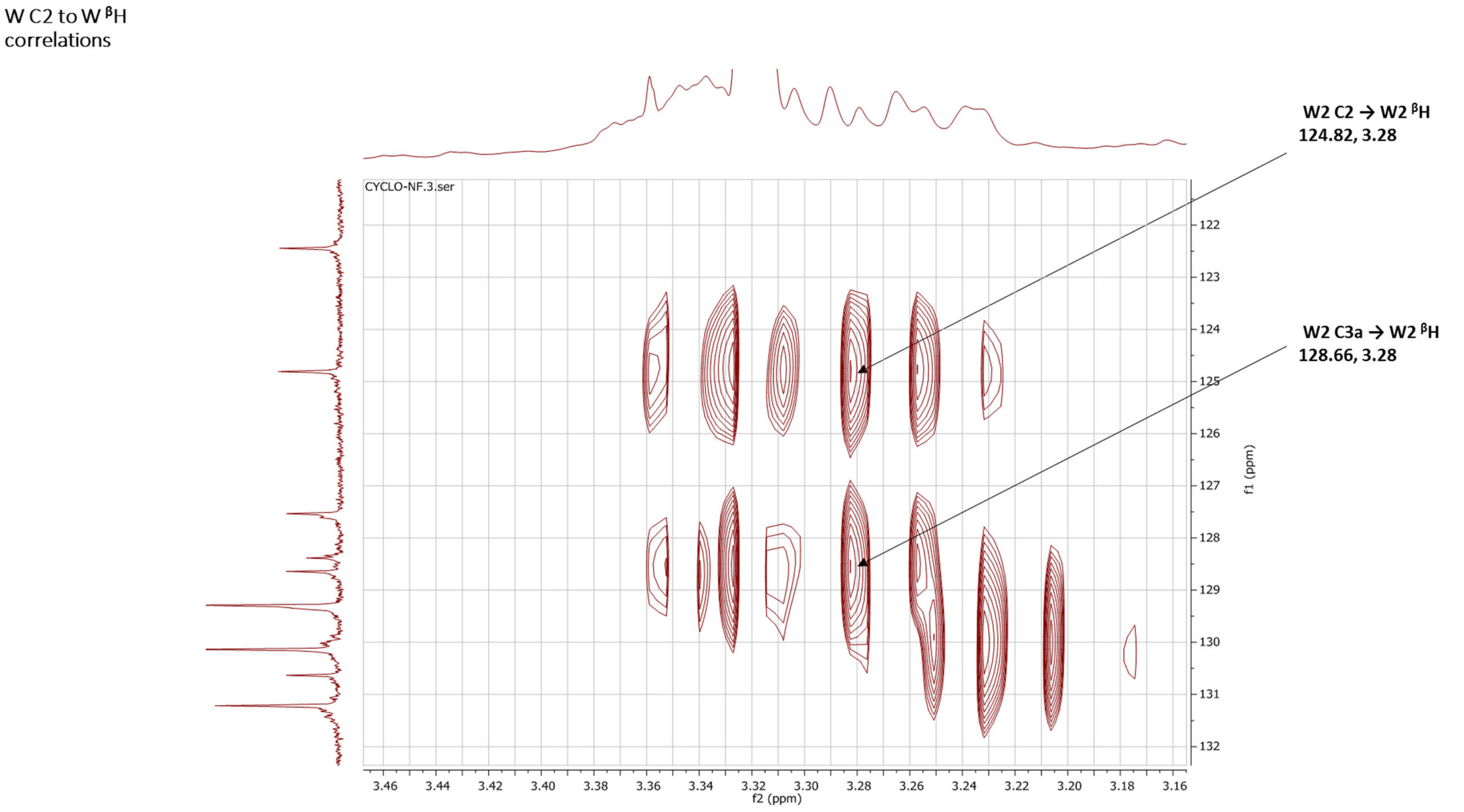

Figure S3. 22: HMBC correlations of [1] showing residue W2 side chain (2).

W C2 to Indole NH
correlations

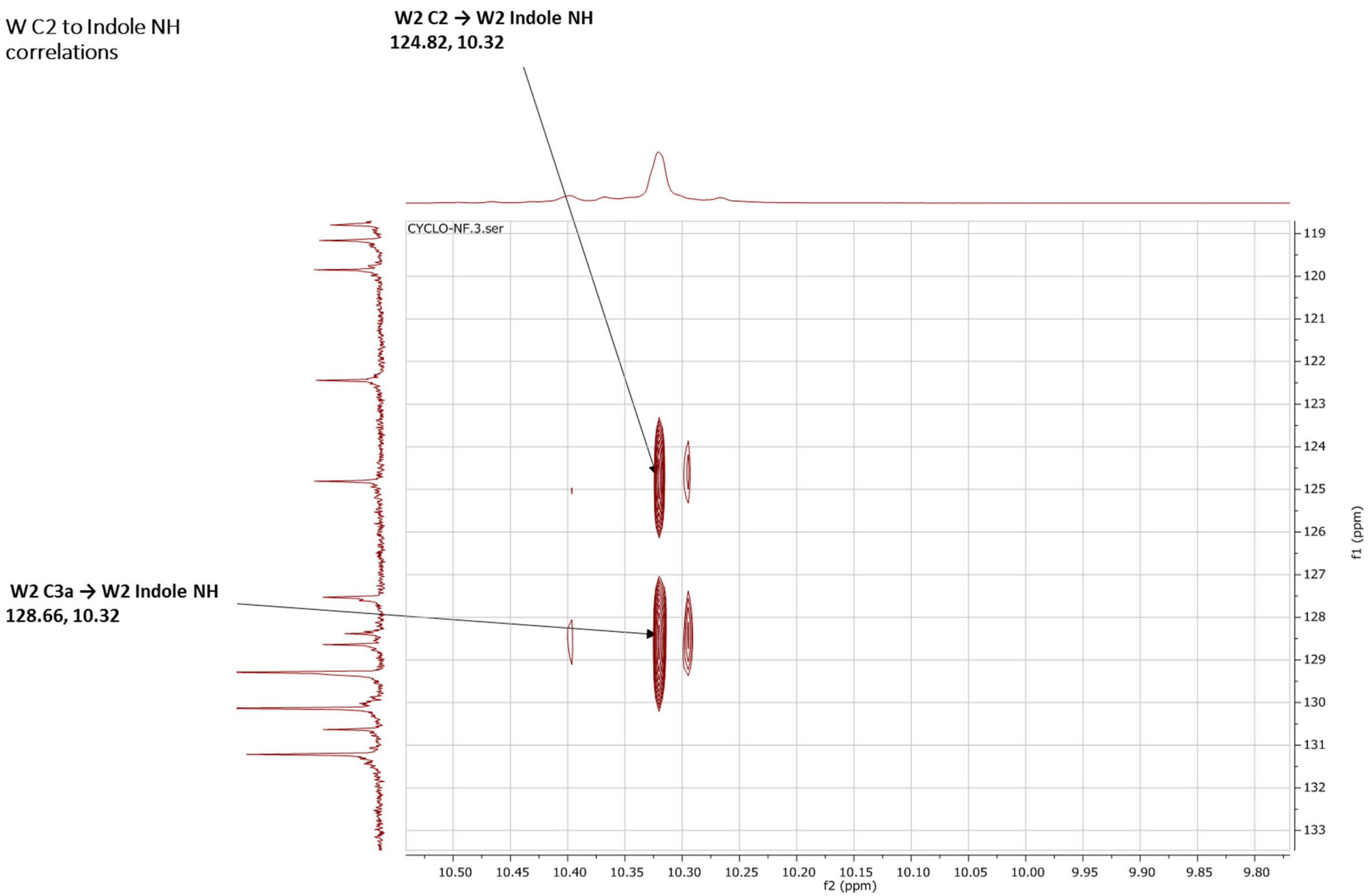

Figure S3. 23: HMBC correlations of [1] showing residue W2 side chain (3).

W C7a to Indole NH
correlations

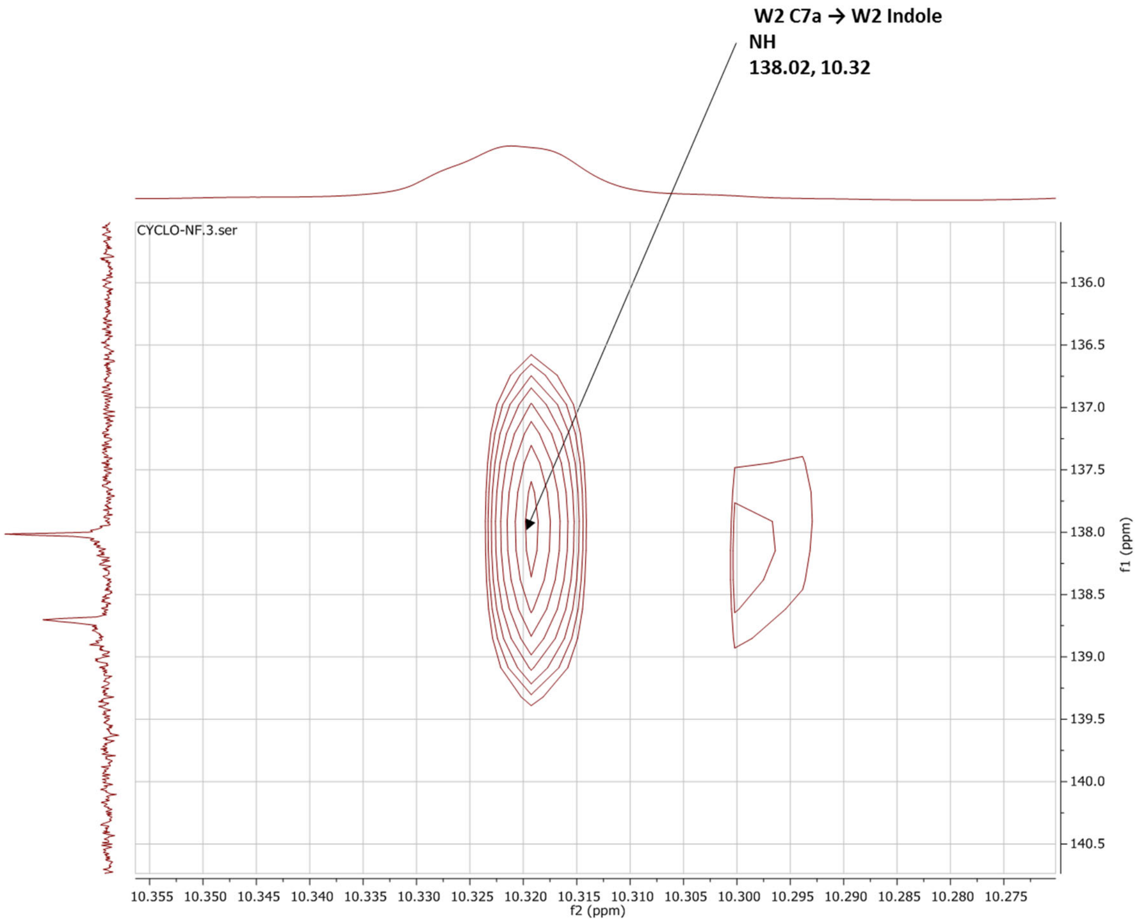

Figure S3. 24: HMBC correlation of [1] showing residue W2 side chain (4).

W C7a to C4 H
correlations

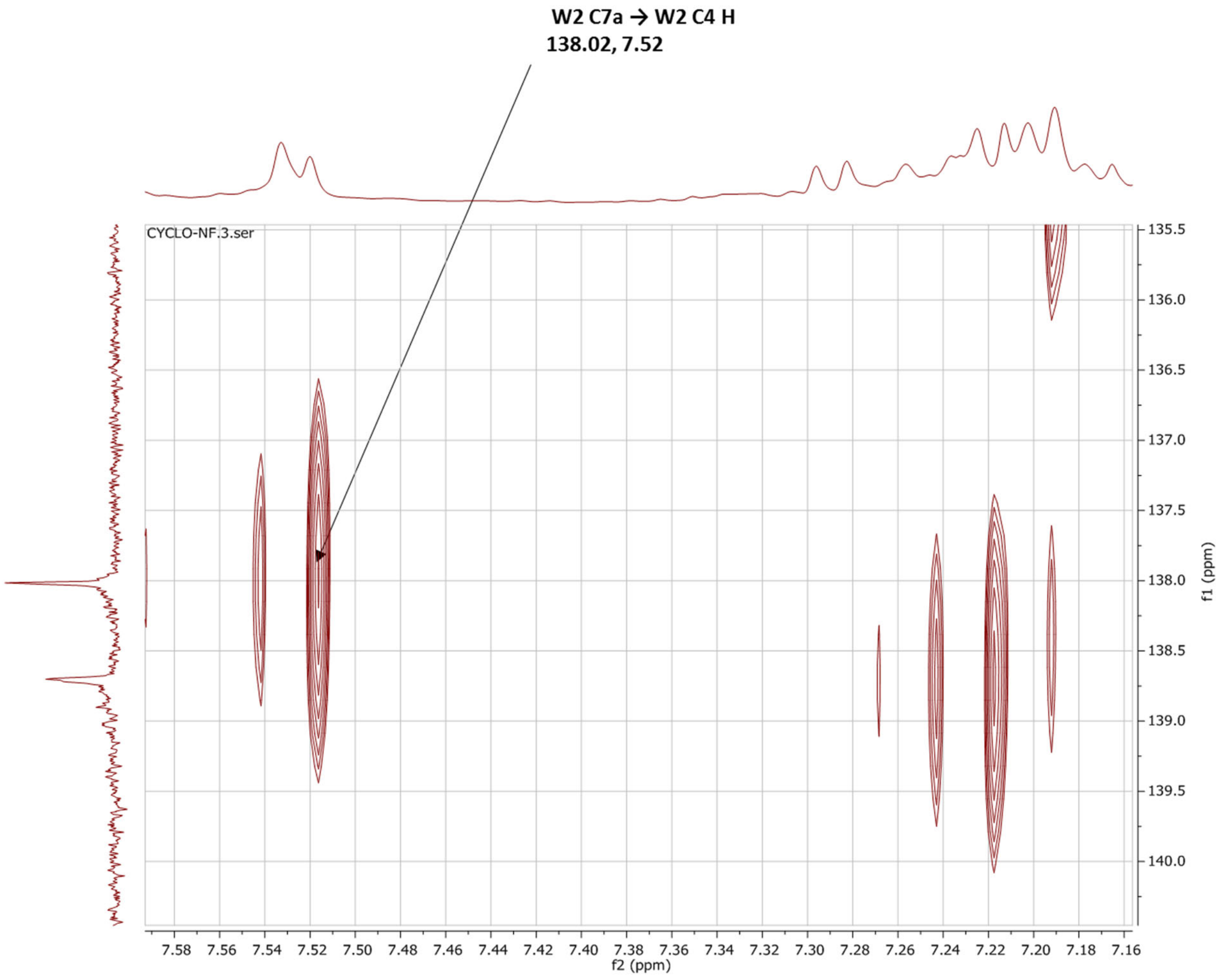

Figure S3. 25: HMBC correlation of [1] showing residue W2 side chain (5).

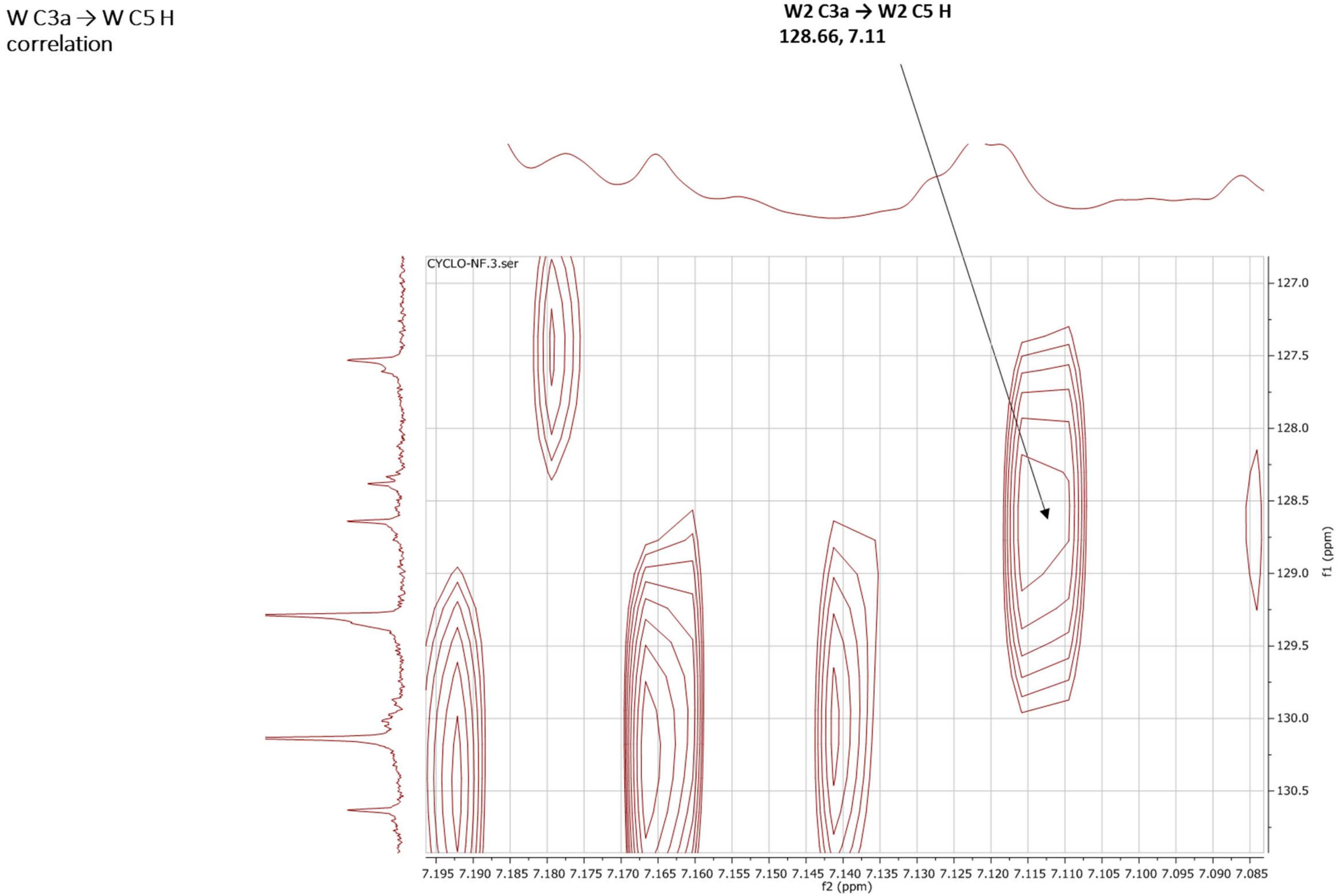

Figure S3. 26: HMBC correlation of [1] showing residue W2 side chain (6).

W C7a to C6 H
correlations

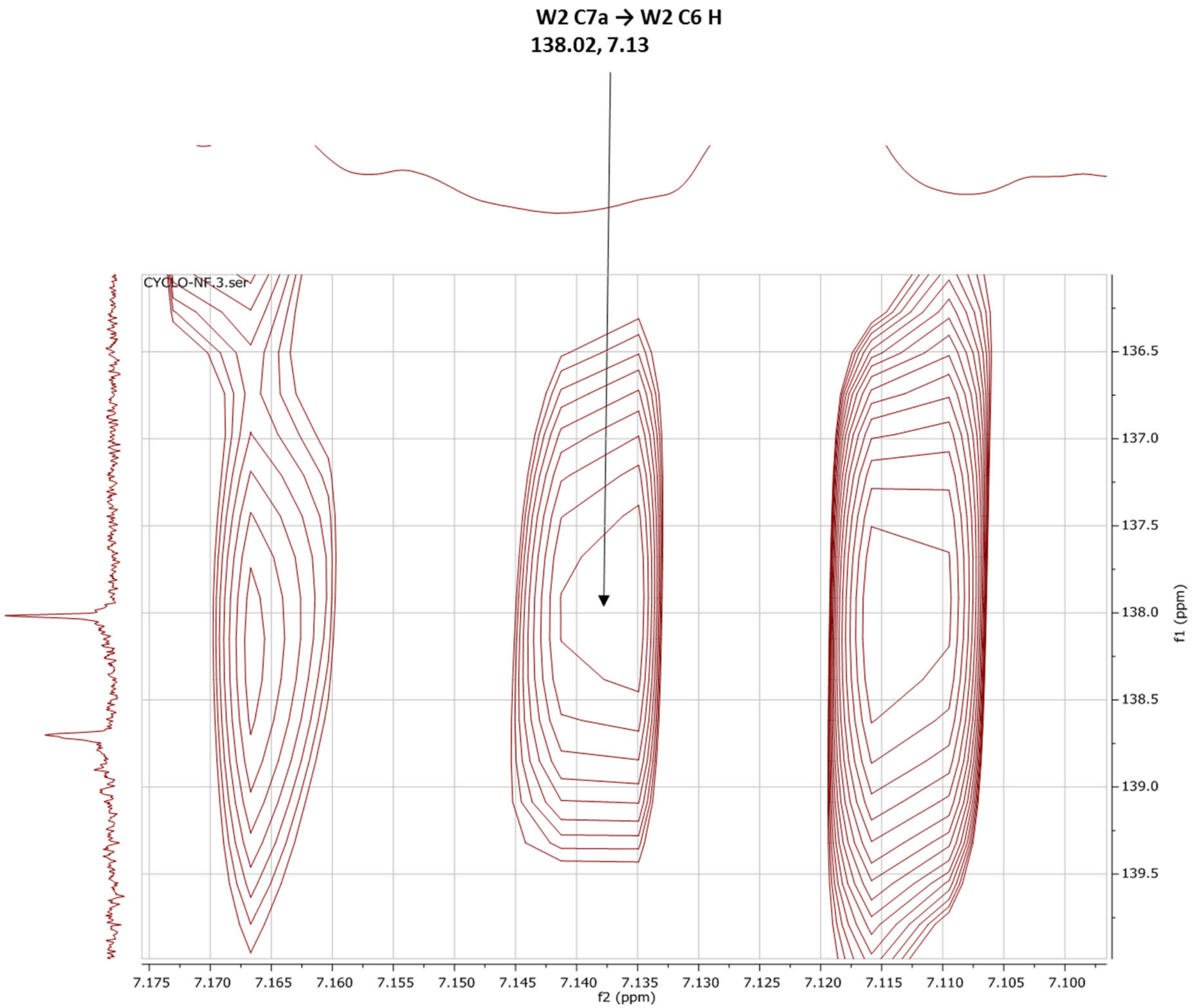

Figure S3. 27: HMBC correlation of [1] showing residue W2 side chain (7).

W C7 → W C5 H
correlation

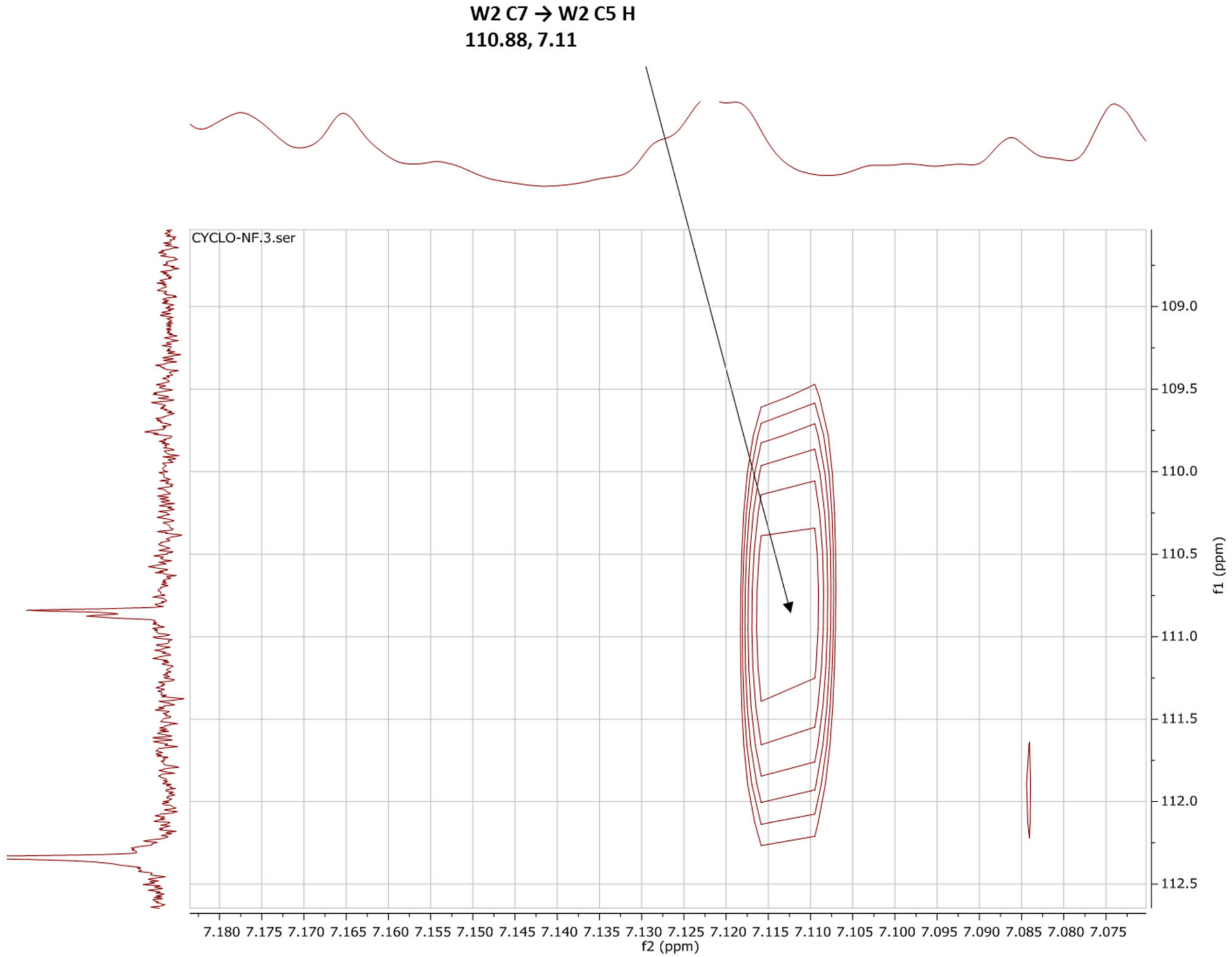

Figure S3. 28: HMBC correlation of [1] showing residue W2 side chain (7).

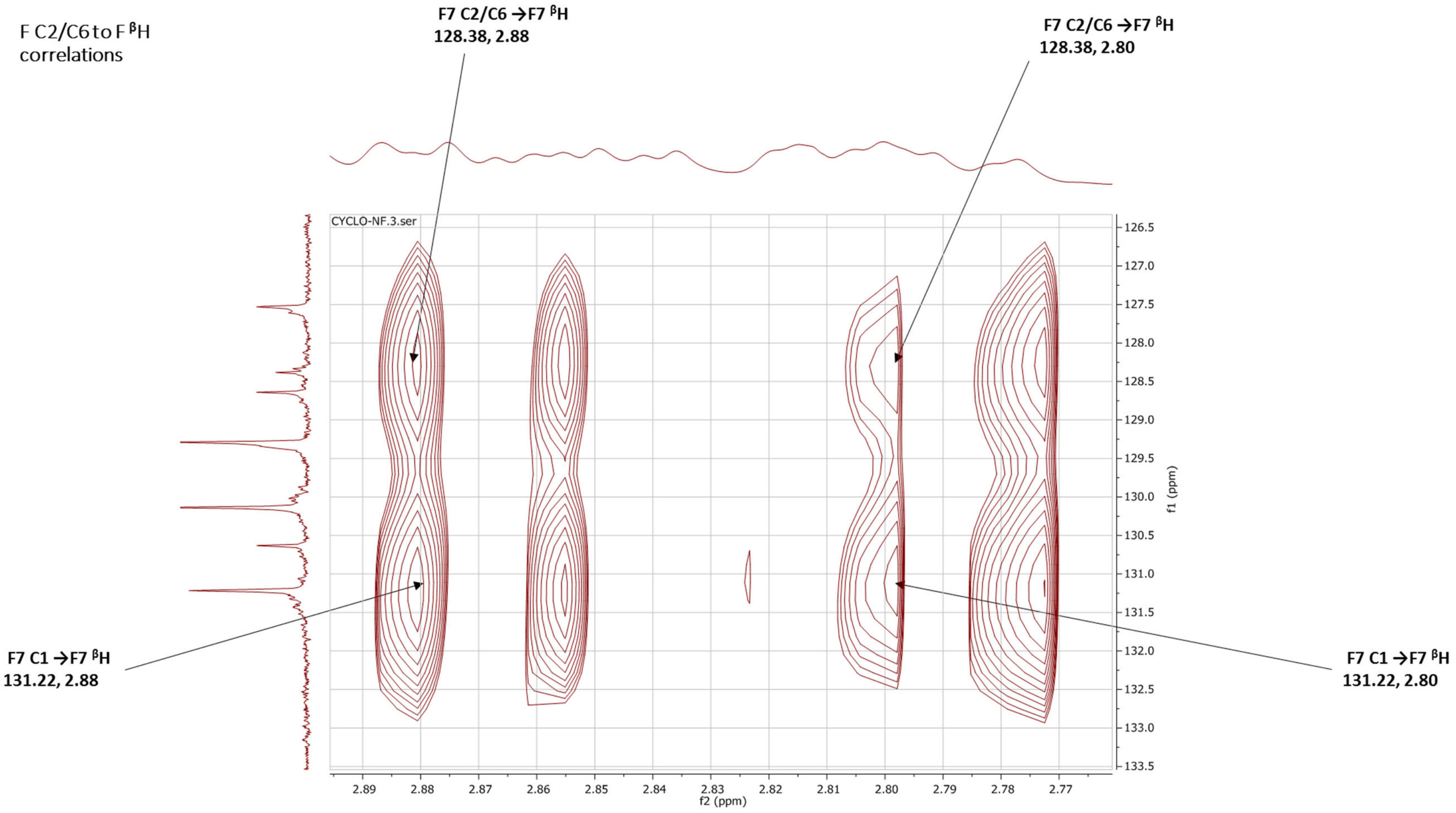

Figure S3. 29: HMBC correlations of [1] showing residue F7 side chain (1).

F C2/C6 to F C3/C5 H correlations

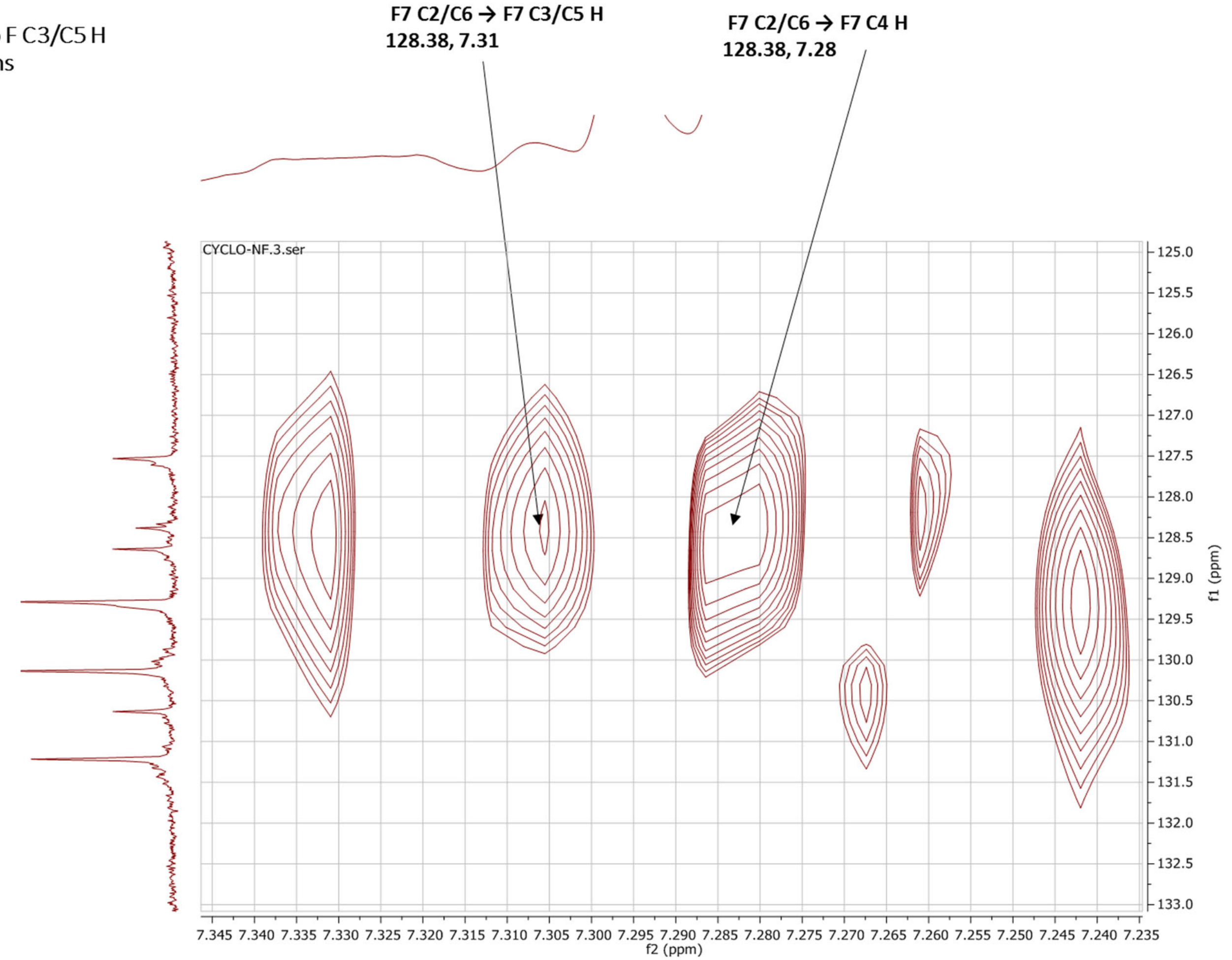

Figure S3. 30: HMBC correlations of [1] showing residue F7 side chain (2).

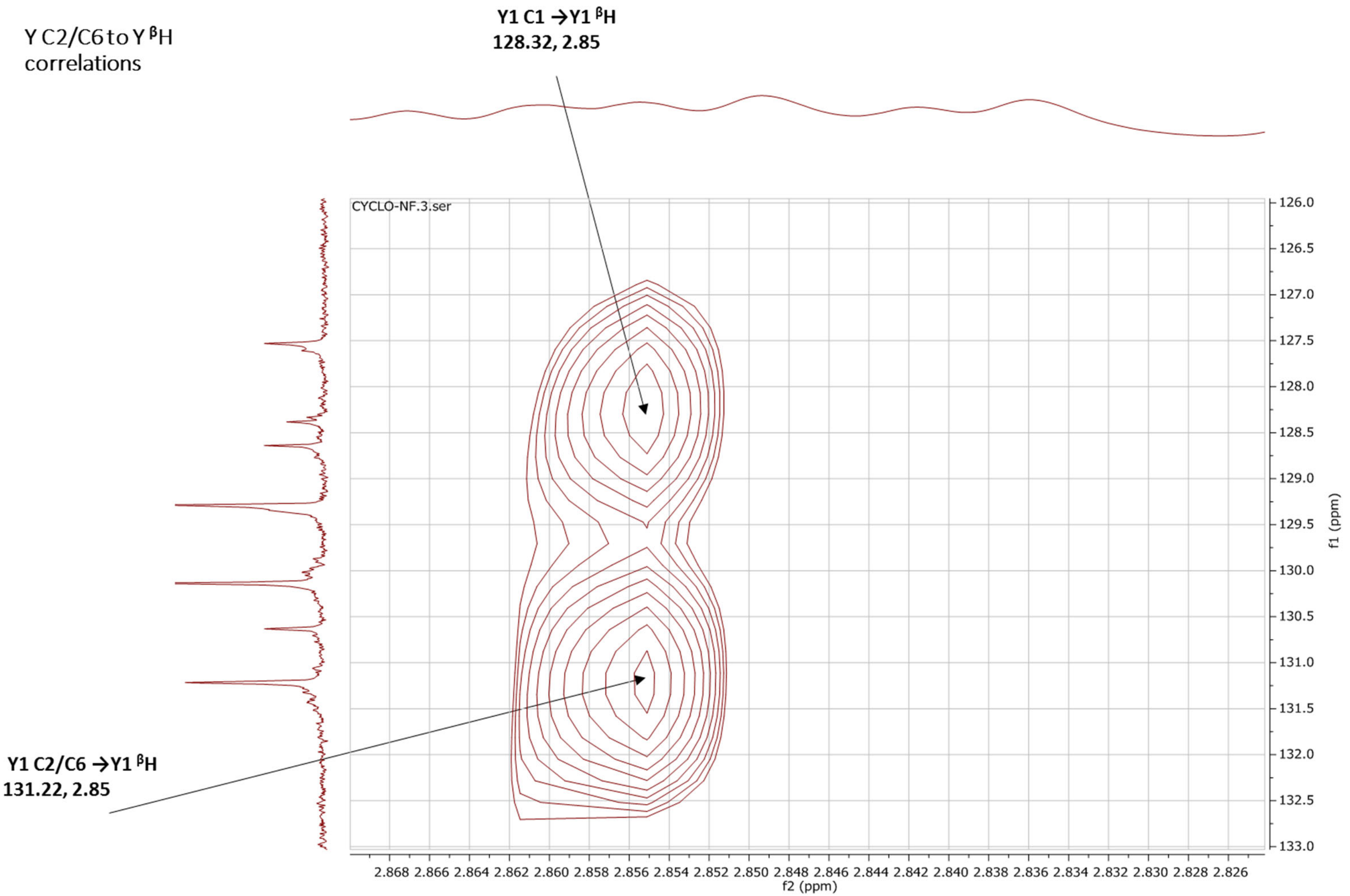

Figure S3. 31: HMBC correlations of [1] showing residue Y1 side chain (1).

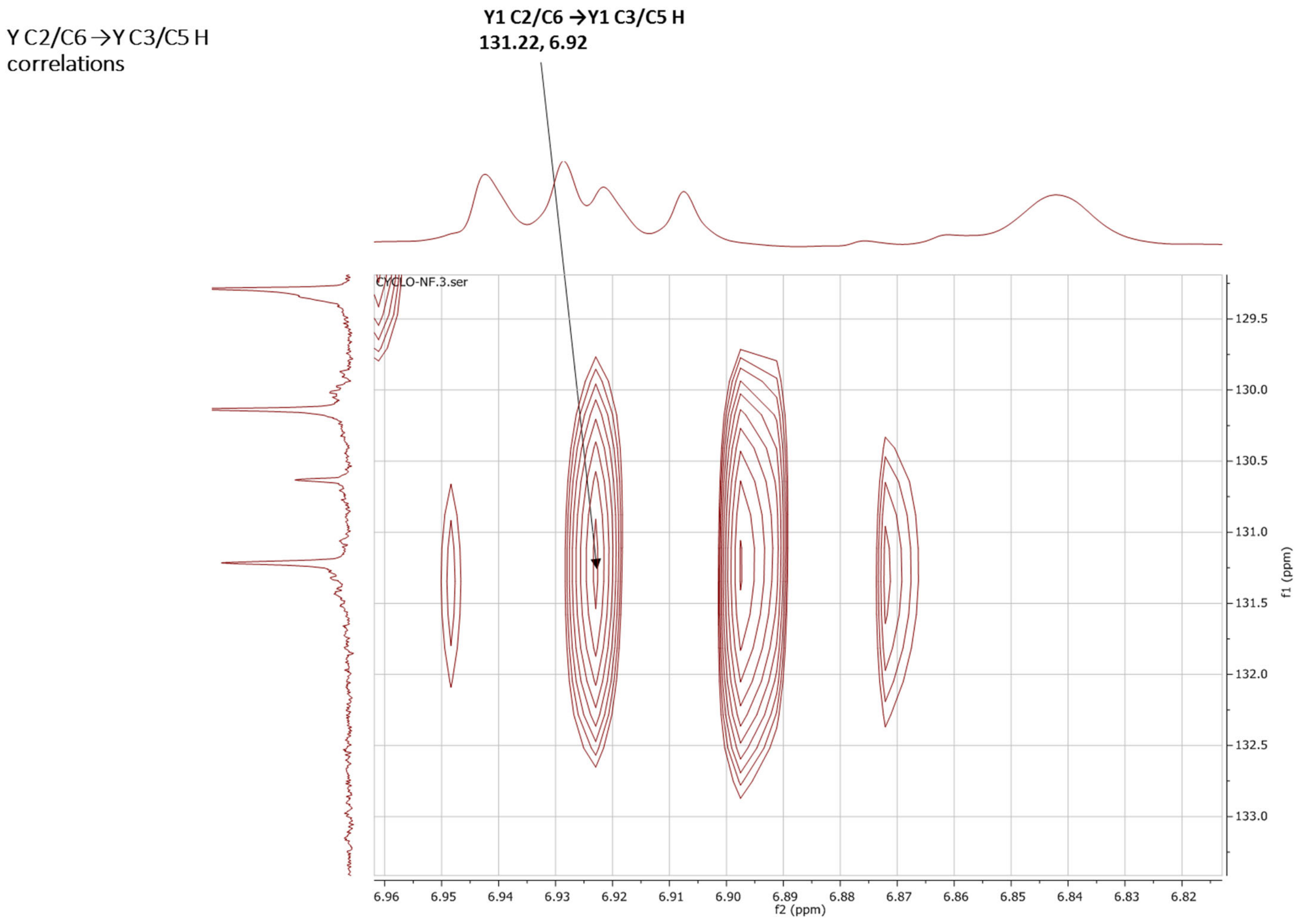

Figure S3. 32: HMBC correlation of [1] showing residue Y1 side chain (2).

Y C4 →Y C3/C5 H
correlations

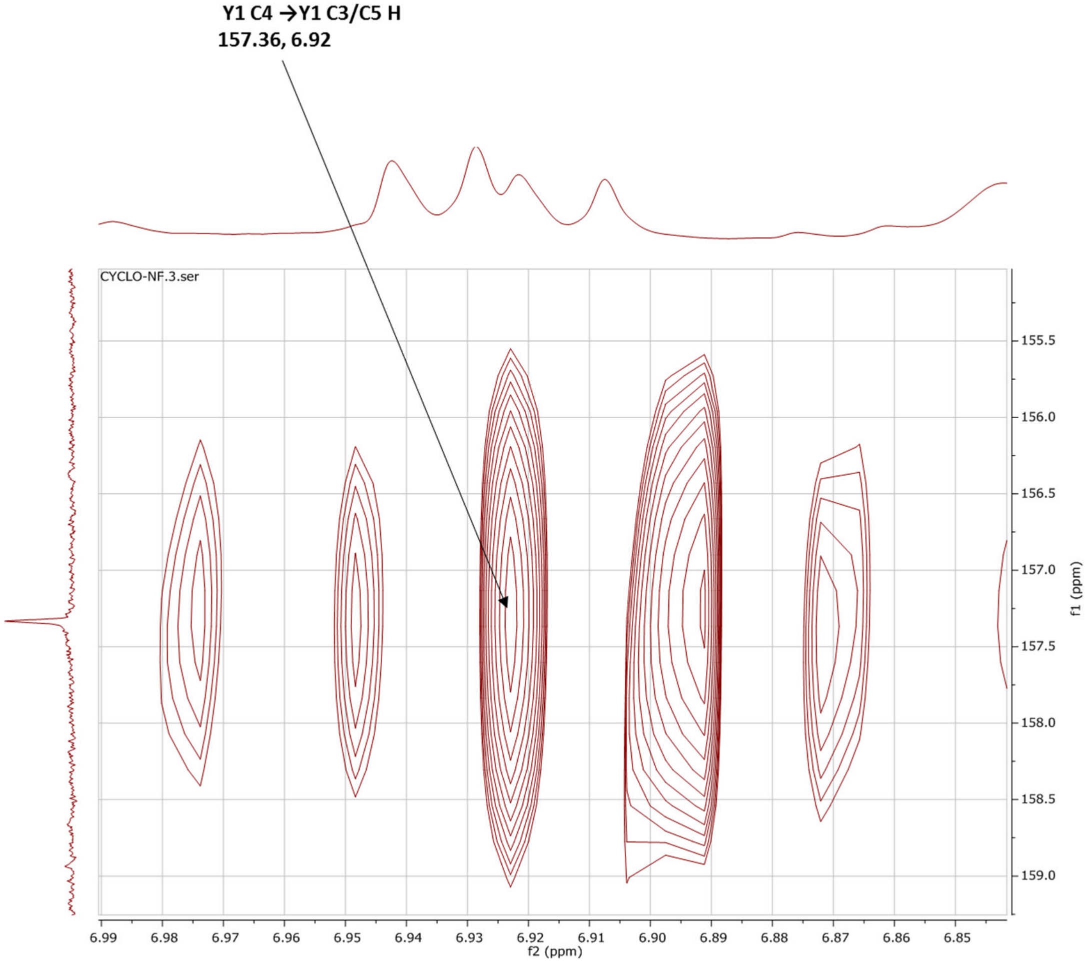

Figure S3. 33: HMBC correlation of [1] showing residue Y1 side chain (3).

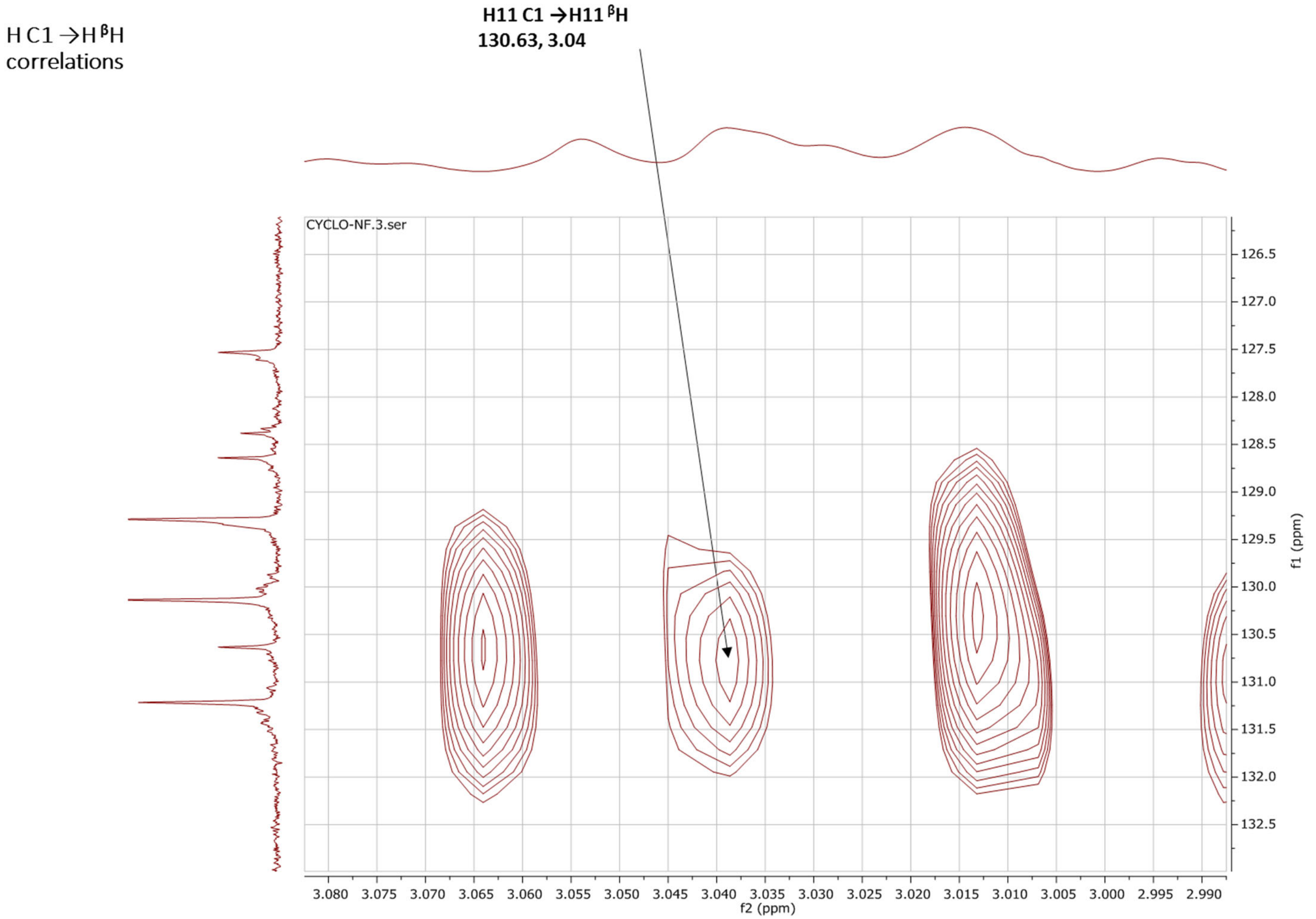

Figure S3. 34: HMBC correlation of [1] showing residue H11 side chain (1).

H C5 →H $^{\beta}$H
correlations

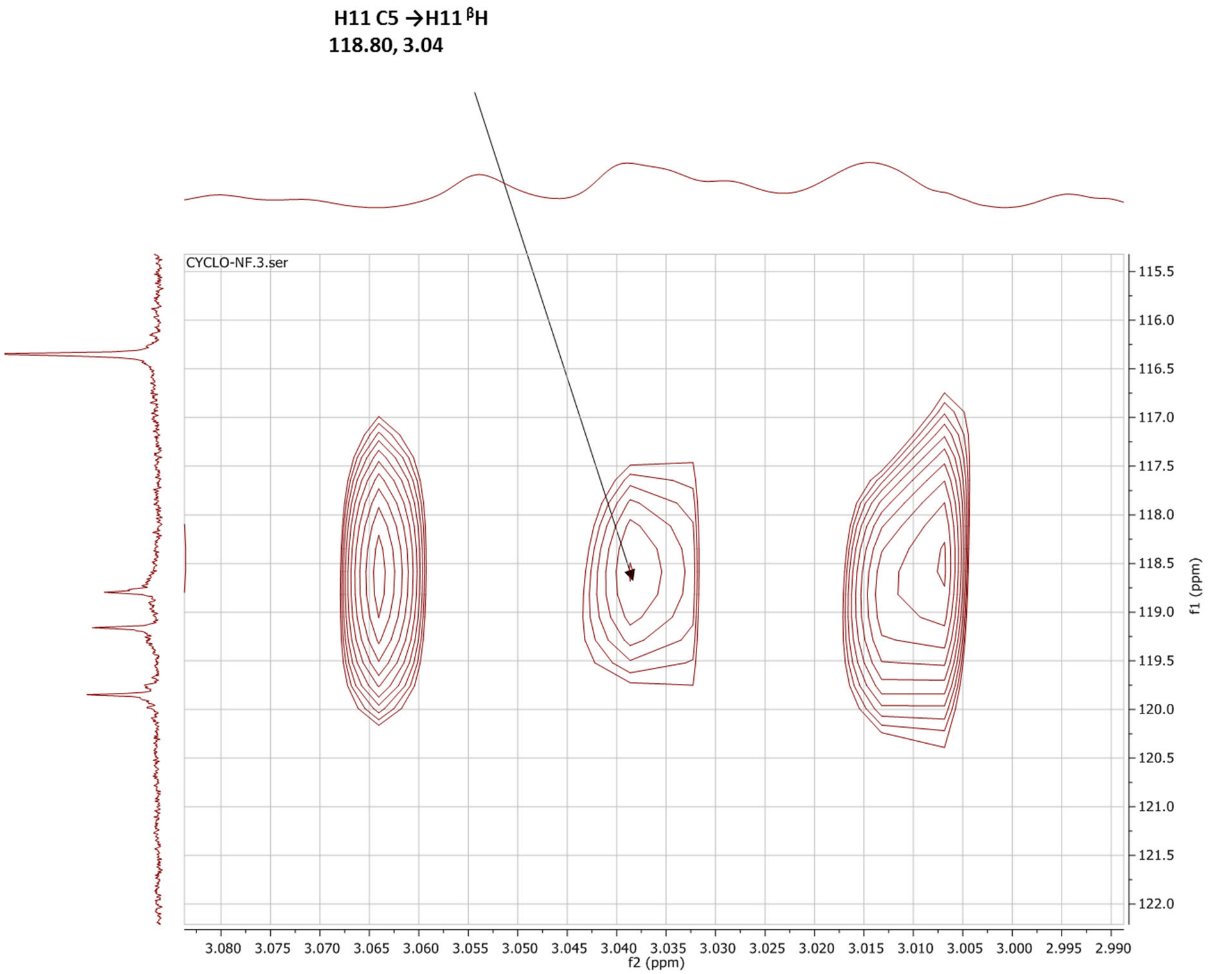

Figure S3. 35: HMBC correlation of [1] showing residue H11 side chain (2).

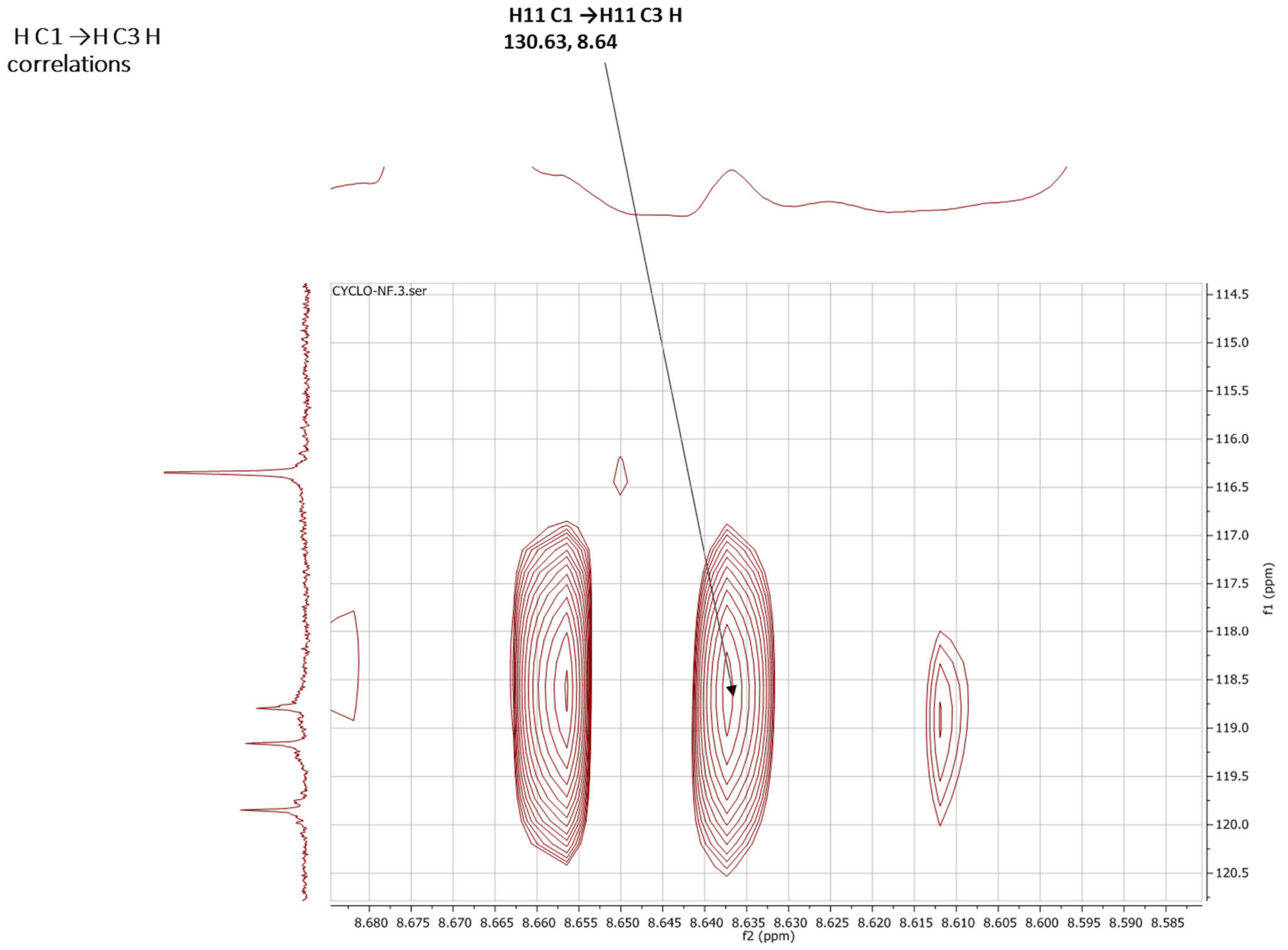

Figure S3. 36: HMBC correlation of [1] showing residue H11 side chain (3).

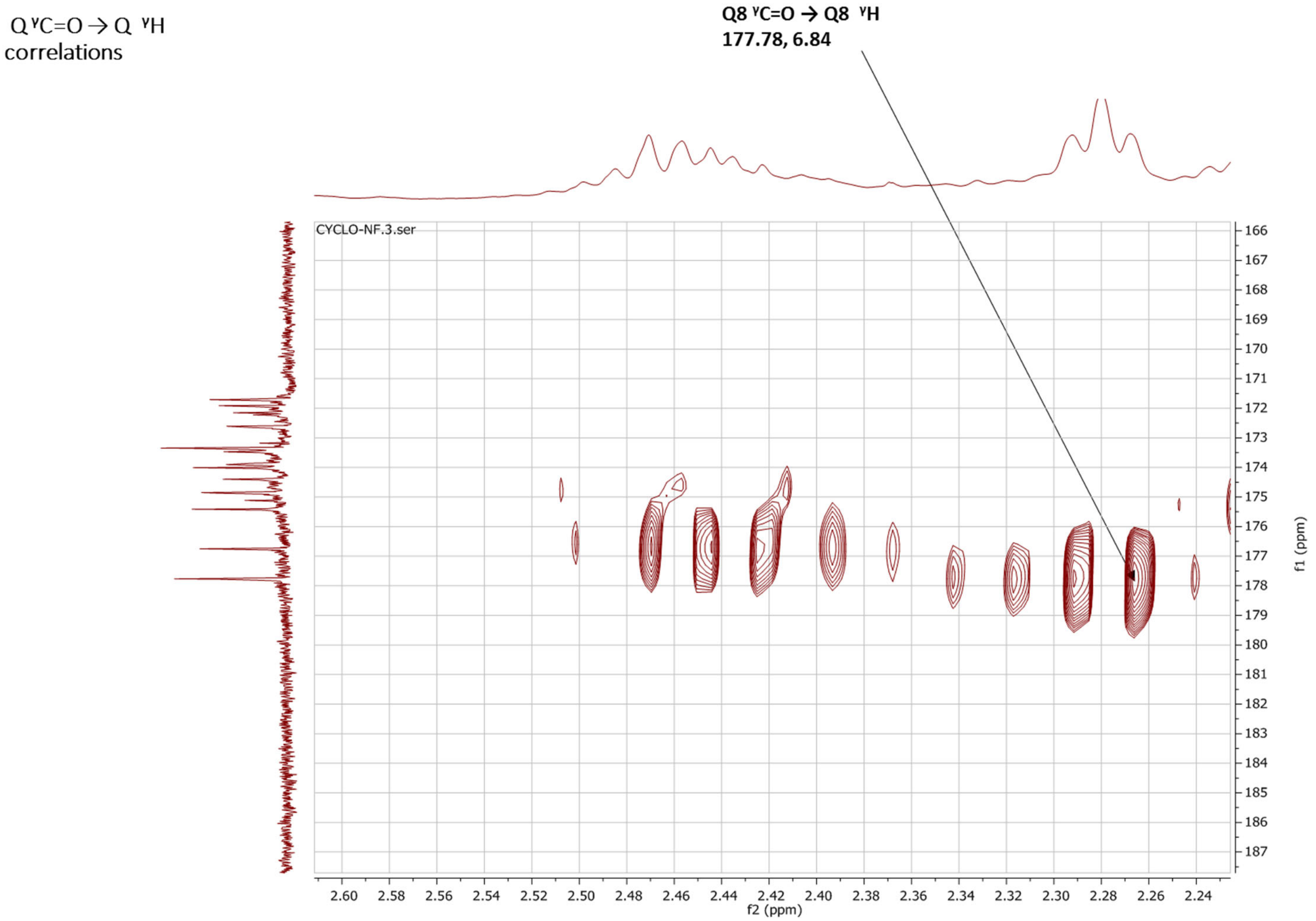

Figure S3. 37: HMBC correlation of [1] showing residue Q8 side chain (1).

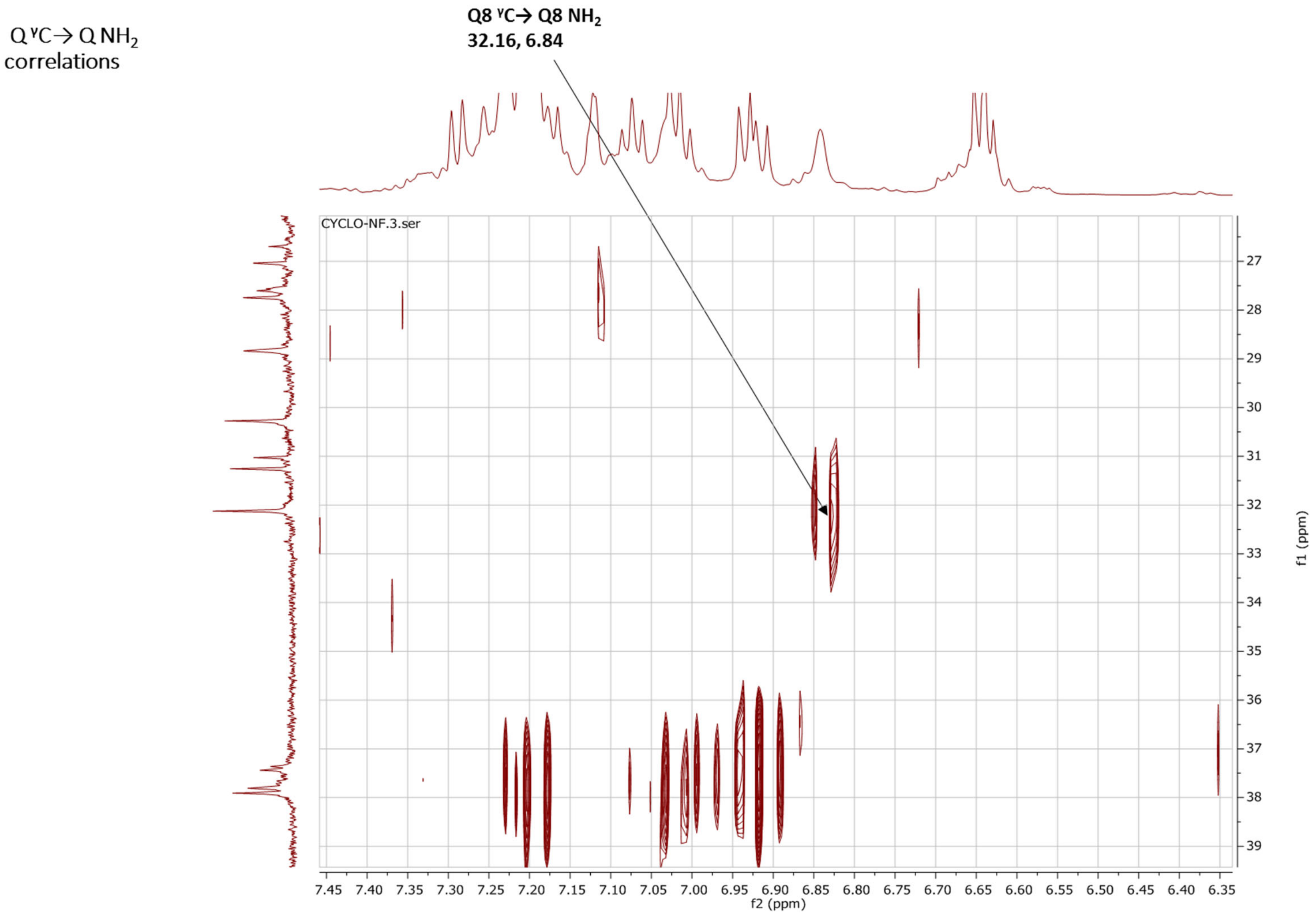

Figure S3. 38: HMBC correlation of [1] showing residue Q8 side chain (2).

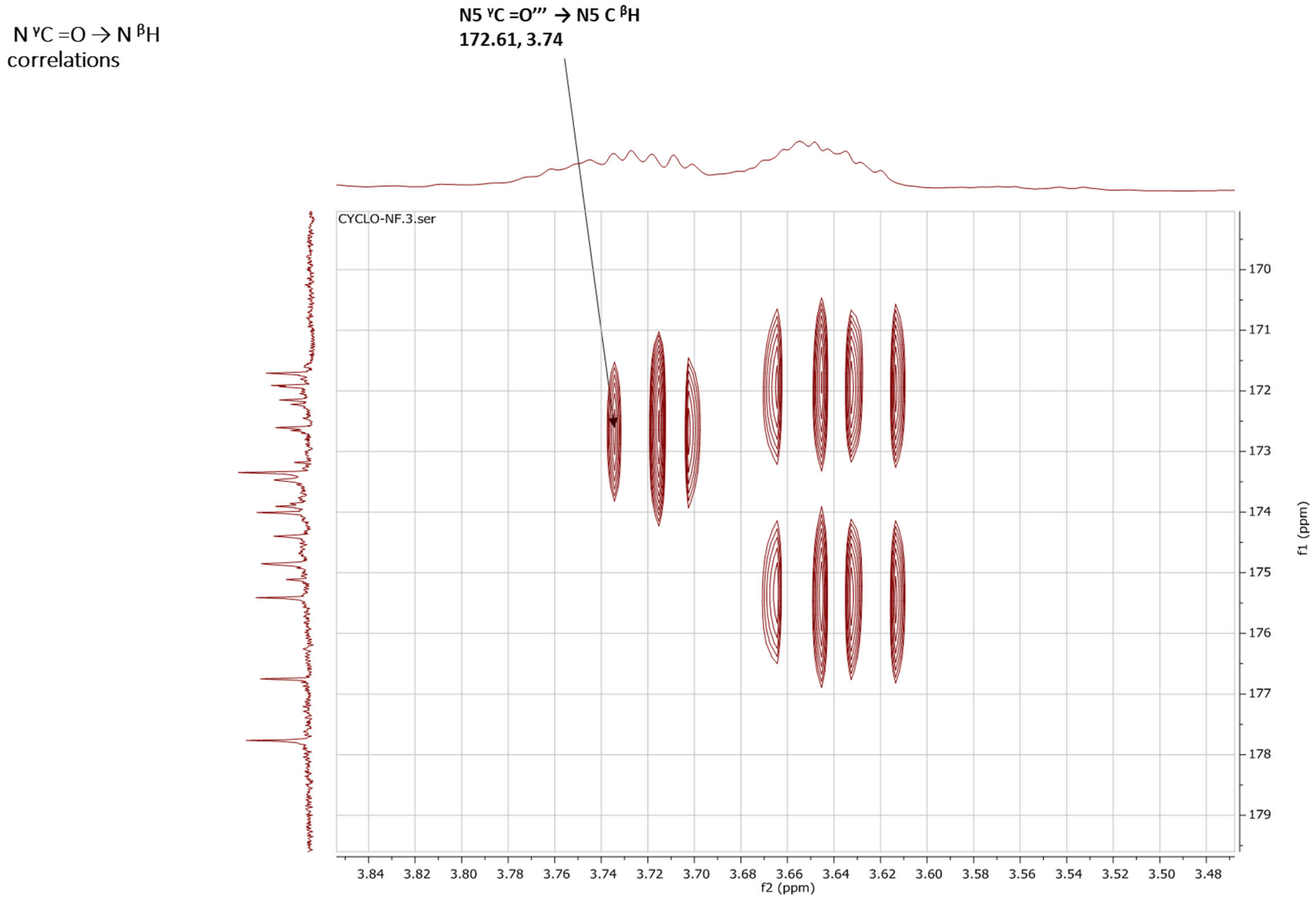

Figure S3. 39: HMBC correlation of [1] showing residue N5 side chain (1).

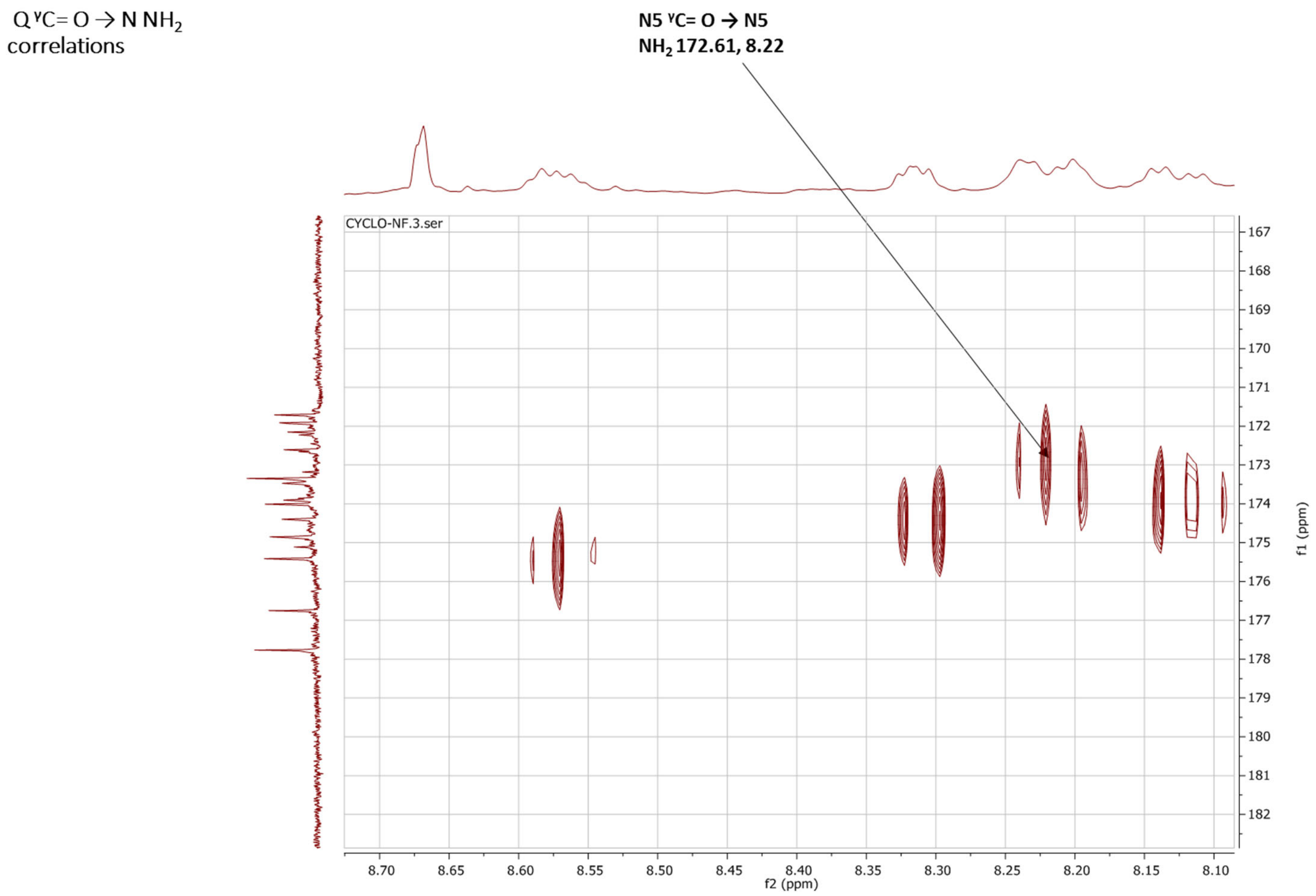

Figure S3. 40: HMBC correlation of [1] showing residue N5 side chain (2).

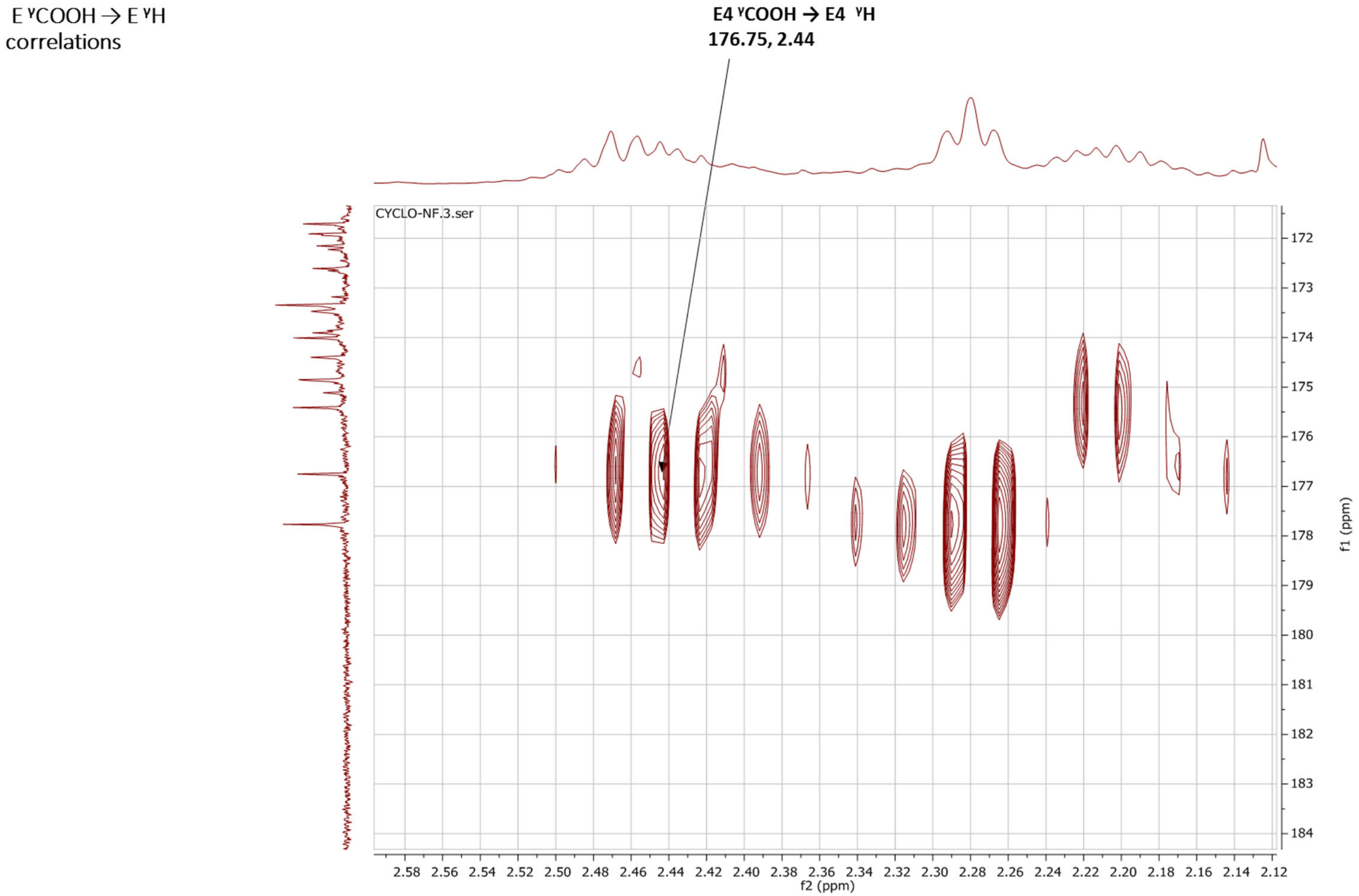

Figure S3. 41: HMBC correlation of [1] showing residue E4 side chain.

S $^{\beta}$C → S –OH
correlations

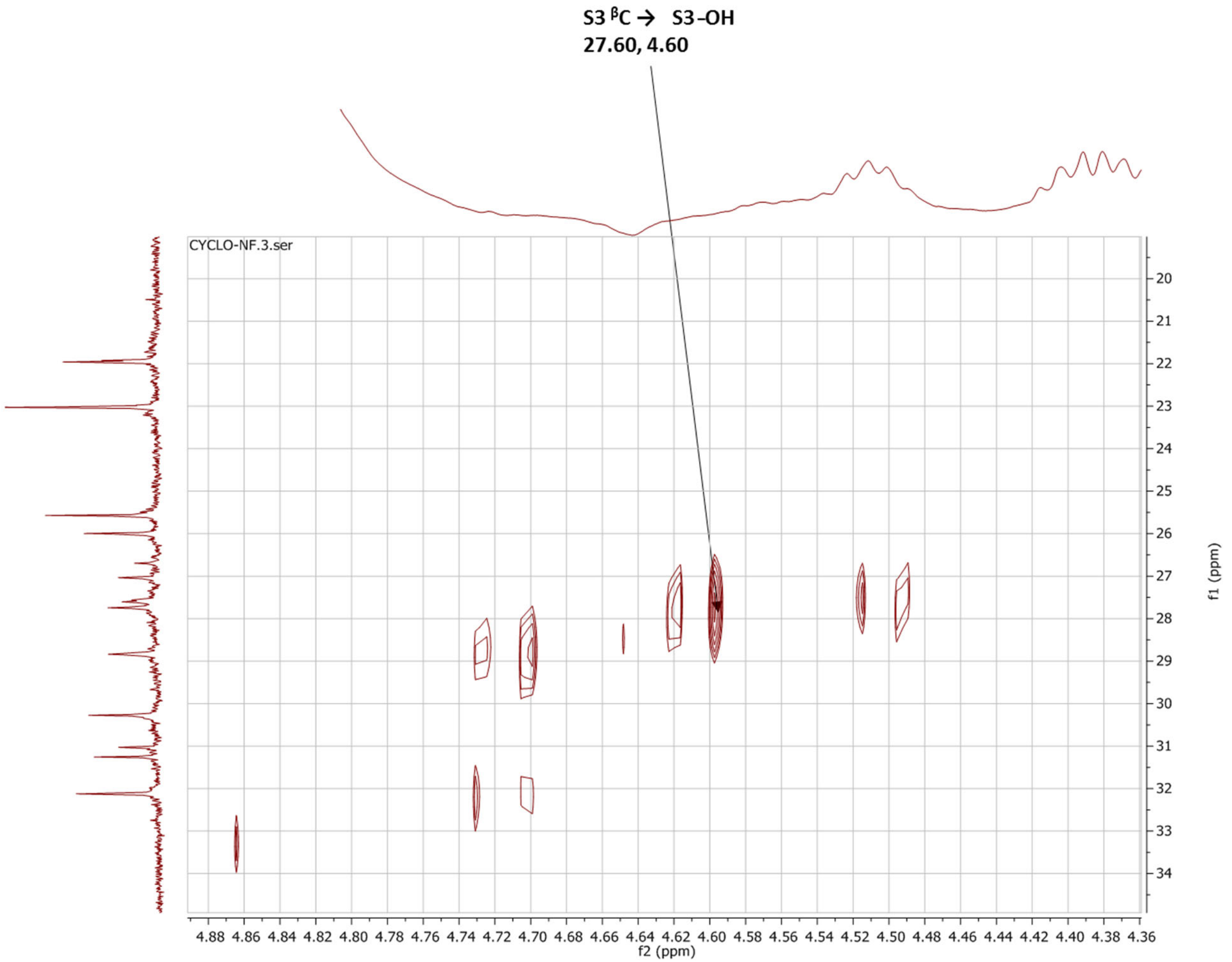

Figure S3. 42: HMBC correlation of [1] showing residue S3 side chain.

NMR 3D structure of [1]

The good quality structures of **[1]** resulting from simulated annealing were found to be clustered into 2 groups of conformations following main chain alignment (Figure S4.1 and S4.2), with one set of 7 conformations (A) and the other with 3 conformations (B). RMSD values of aligned structures (main chain) were less than 1 Å for each set. All 10 structures showed no significant violations of NOEs, bonds, angles, impropers, Van der Waal, and total energies.

Ramachandran plots (Figure S4.3 and S4.4) were constructed, to show the distribution of the torsional angles phi and psi of **[1]** clustered solution structures between energitically allowed and disallowed regions of the plot. Violations of dihedral angles in group (A) NMR structures were found at the residues Glu4 (5 conformations), Gly10 (5 conformations), Leu6 (5 conformations) and Trp2 (1 conformation), while violations of group (B) NMR structures were at residues Glu4 (3 conformations), Leu6 (3 conformations) and Gly10 (2 conformations).

The structural difference between the best NMR structures in each group was found to be 3.05 Å following backbone alignment of each structure. These were then selected based on the violation statistics in the calculations (Table S4.2). Detailed NOESY correlations from which the NOE restraints were obtained are shown in Figure SI 4.5-4.

Table S4. 1: NOE restraints used for [1] solution 3D structure calculations (# indicates any of the protons in a -CH_2).

Residue 1	Atom 1	Residue 2	Atom 2	Distance (Å)
G10	HN	P9	HD#	4.00
G10	HN	P9	HA	3.12
W2	HN	G10	HA#	4.20
W2	HN	W2	HB#	3.52
E4	HN	N5	HA	4.06
L6	HN	E4	HA	4.05
L6	HN	W2	HA	4.32
S3	HN	S3	HB#	4.08
F7	HN	H11	HA	3.65
F7	HN	F7	HB1	3.73
F7	HN	H11	HB2	3.74
H11	HN	H11	HB2	3.70
N5	HN	S3	HA	4.25
N5	HN	N5	HA	4.73

Y1	HN	Y1	HB#	4.44
Y1	HN	F7	HB2	4.45
Q8	HN	Q8	HB#	4.22
Q8	HN	P9	HG#	3.70
P9	HA	P9	HB#	4.15

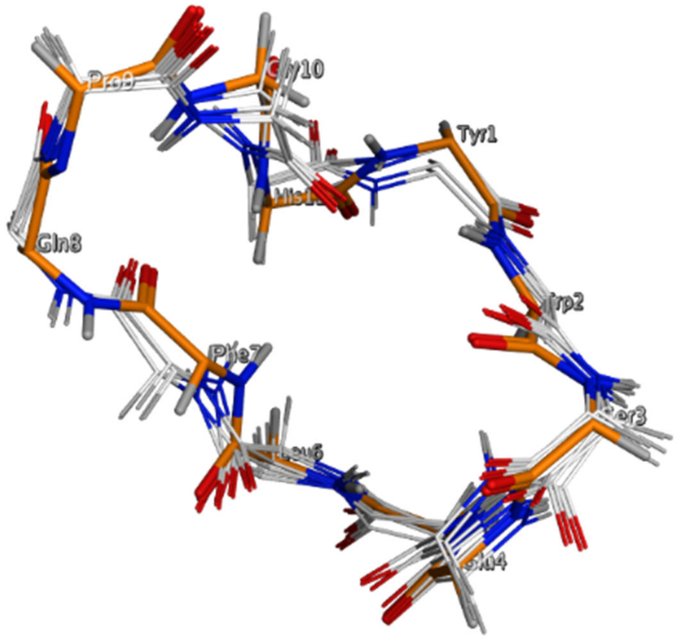

Figure S4. 1: Group (A) NMR conformers of [1] with the main chain aligned and the best structure shown in orange.

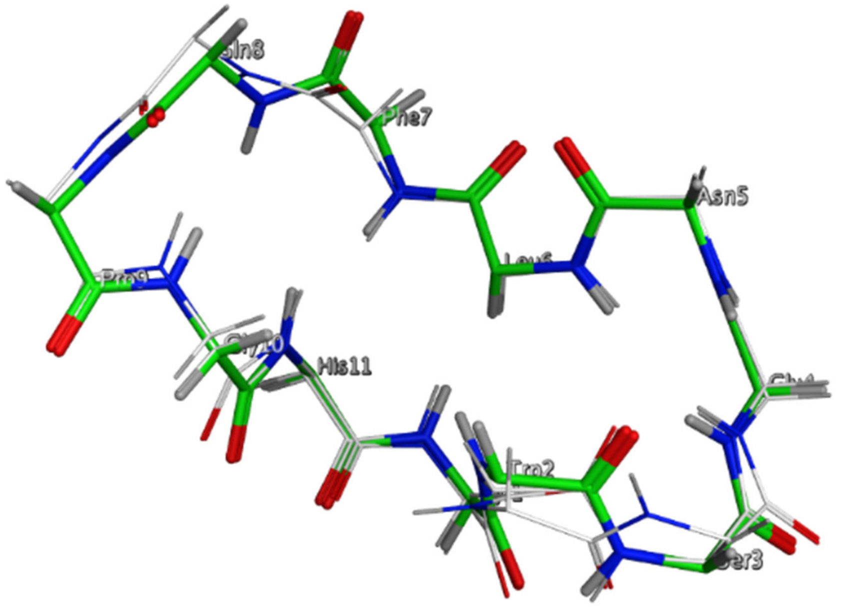

Figure S4. 2: Group (B) conformers of [1] with the main chain aligned and the best structure shown in green.

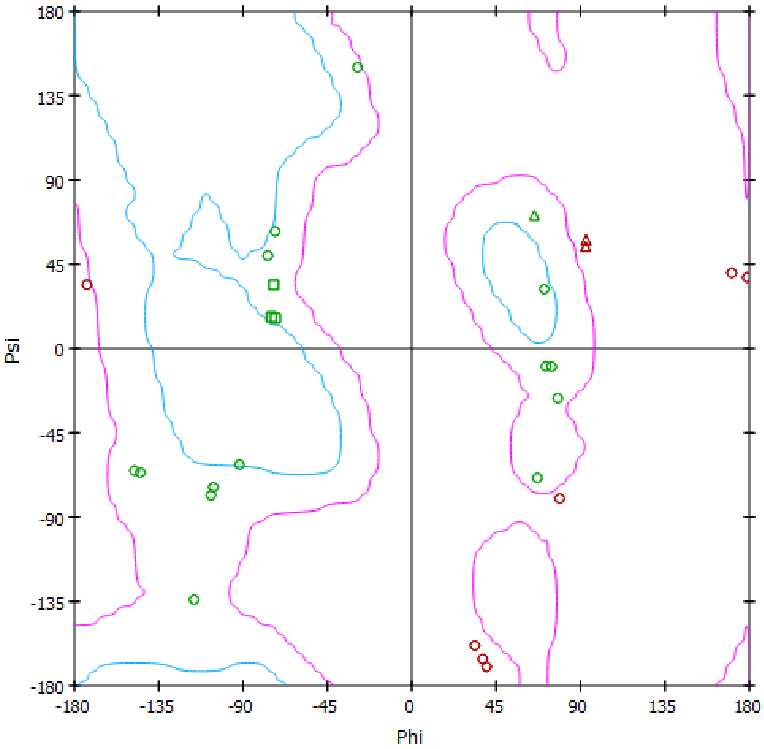

Figure S4. 4: Ramachandran plot of group (A) NMR structures of [1].
The plot shows that all residues lie in allowed/ favoured regions with 16 outliers.

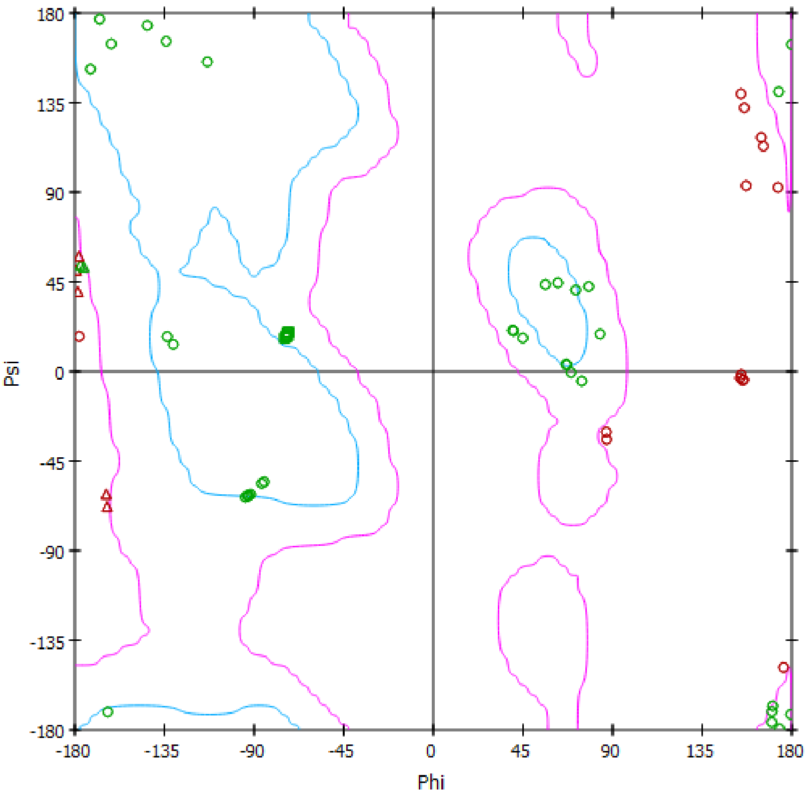

Figure S4. 3: Ramachandran plot of group (B) NMR structures of [1].
The plot shows that all residues lie in allowed/ favoured regions with 8 outliers.

Table S4. 2: Violation statistics for final NMR structures of [1] expressed in kcal/mol.

**Lowest energy Structure**	**Total energy**	**Bonds**	**Angles**	**Improper**	**van der Waal's**	**NOE**
Conformer-30 (group A)	8.14	0.19	7.18	0.77	0.0	0.0
Conformer-67 (group B)	8.31	0.18	7.23	0.79	0.02	0.07

[1]
Y W S E N L F Q P G H

Reference NOESY correlation

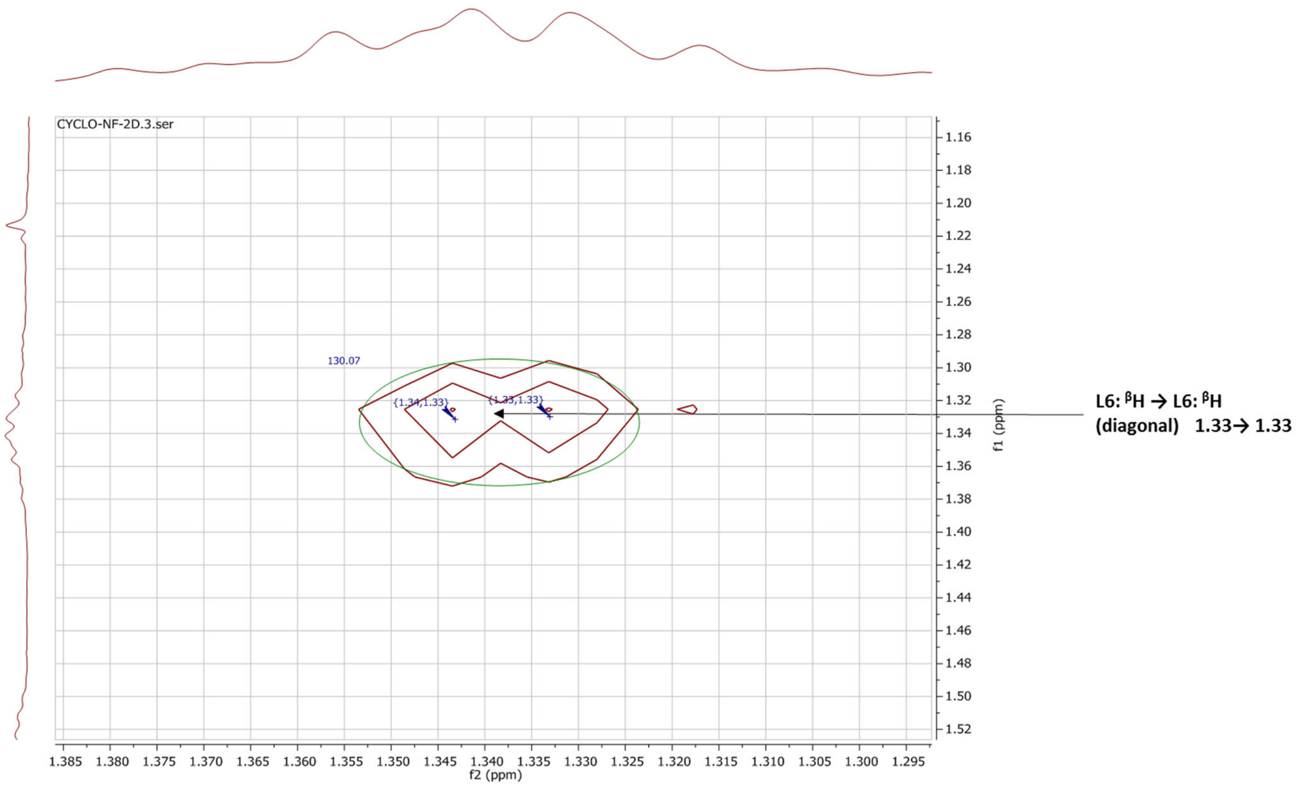

Figure S4. 5: NOESY correlation of [1] showing residue L6 β protons (reference for distance calculations). Mixing time of 300 ms was used.

[1]

Y W S E N L F Q P G H

NOESY

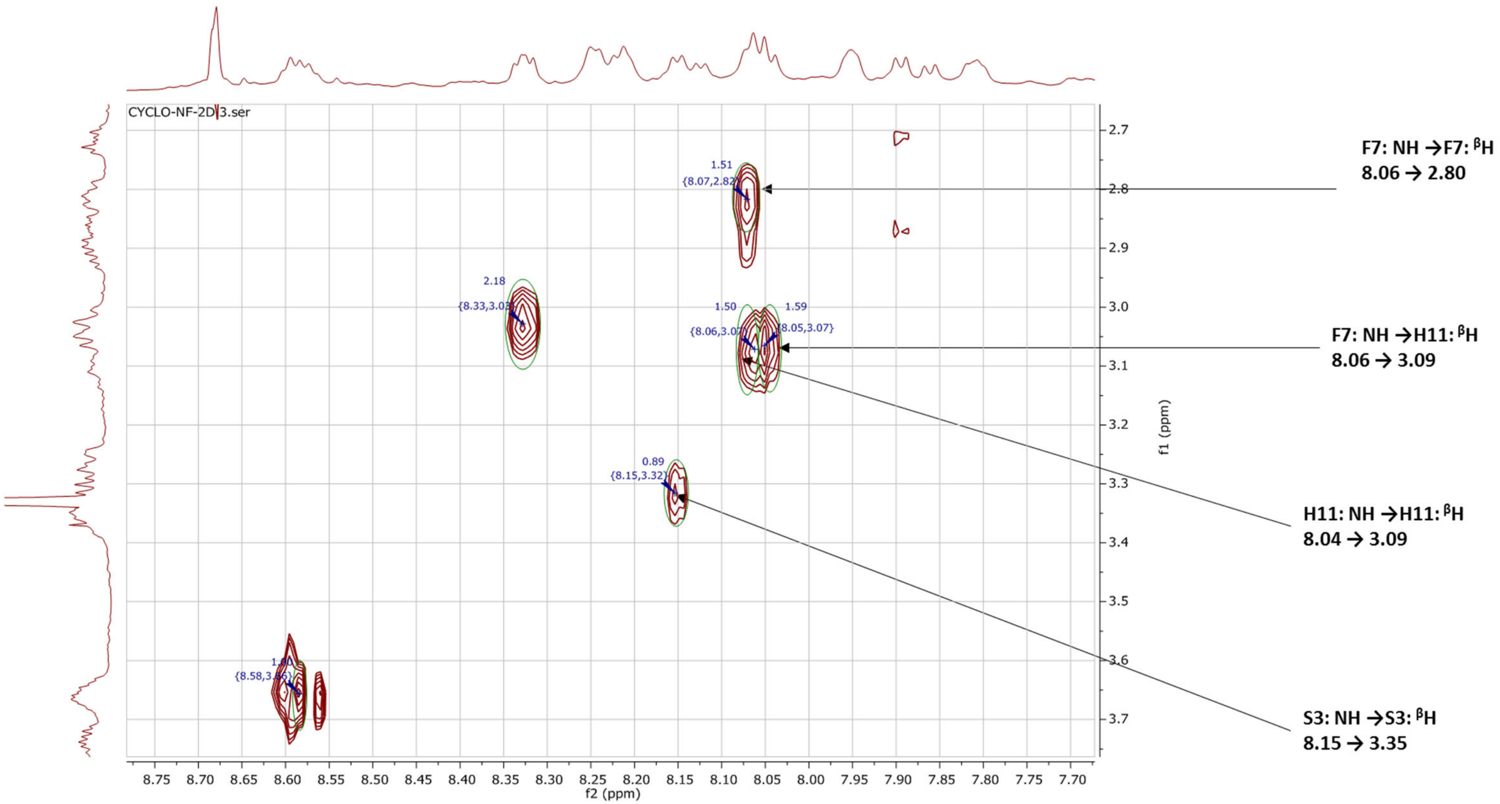

Figure S4. 6: NOESY correlations of [1] showing residue F7, S3 and H11 (main chain NH to βH). Mixing time of 300 ms was used.

[1]
Y W S E N L F Q P G H

NOESY

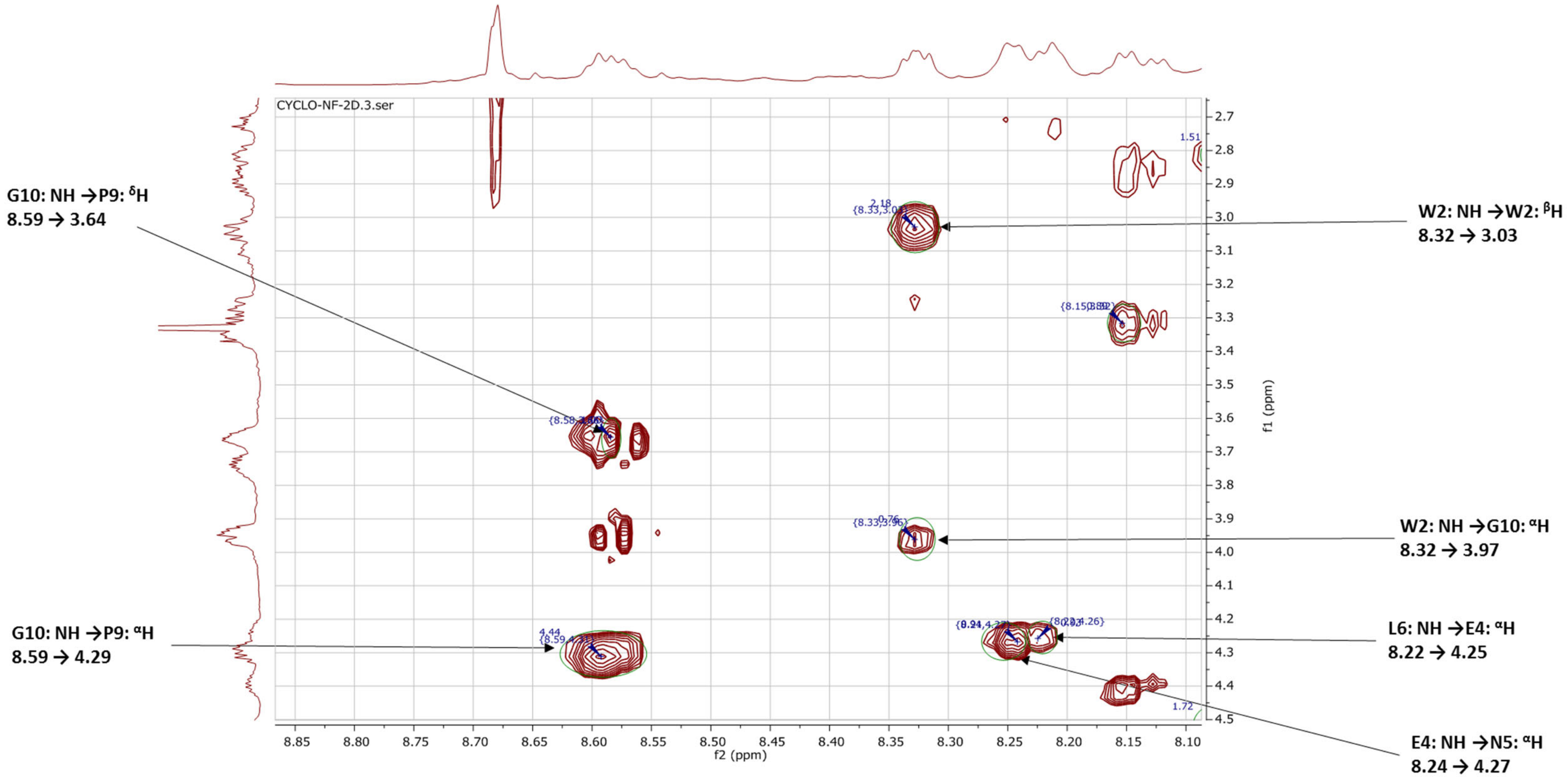

Figure S4. 7: NOESY correlations of [1] showing residue G10, W2, L6, and E4 (main chain NH to α/βH).
Mixing time of 300 ms was used.

[1]
Y W S E N L F Q P G H

NOESY

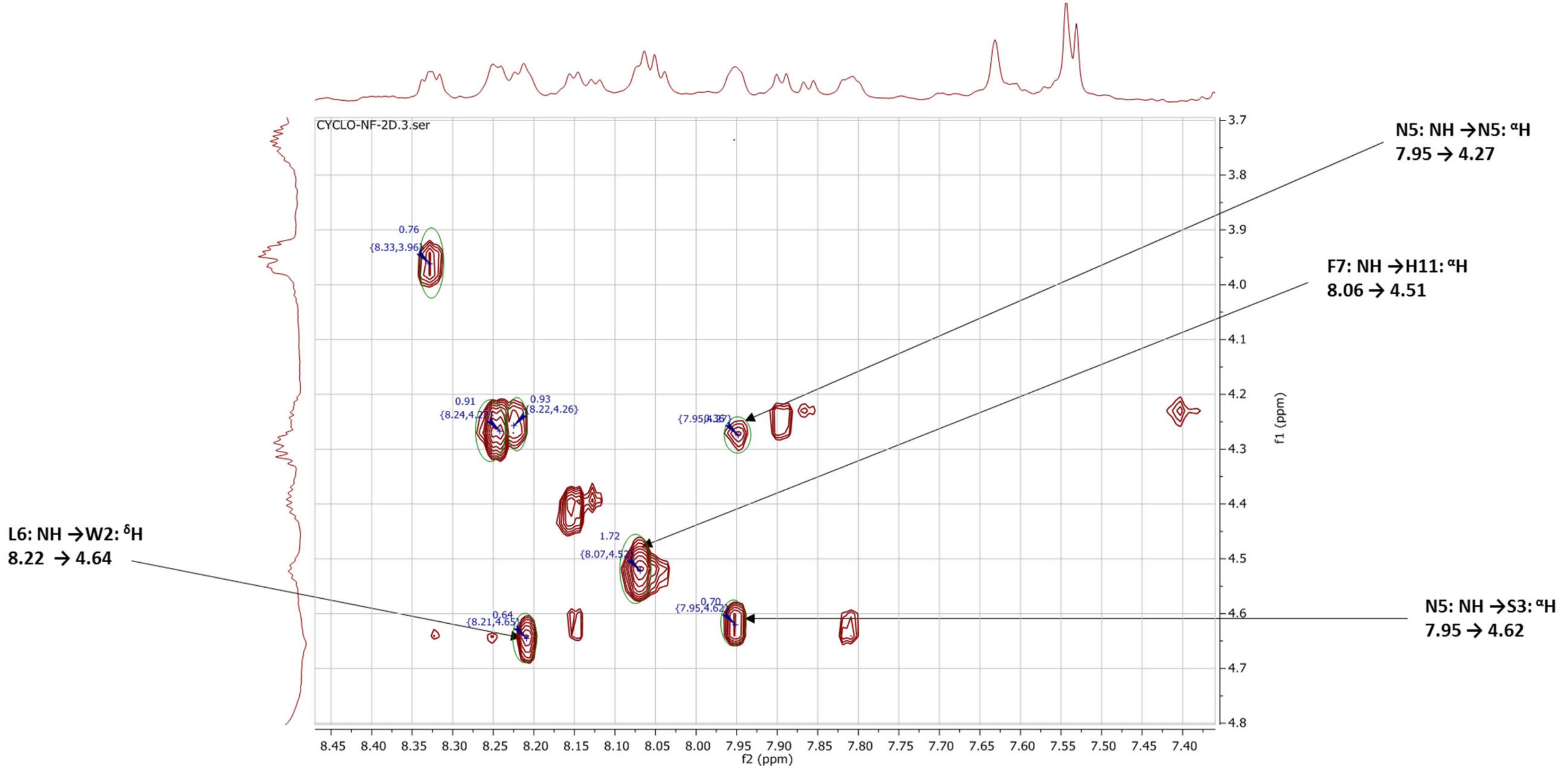

Figure S4. 8: NOESY correlations of [1] showing residue N5, F7and L6 (main chain NH to α/δH).
Mixing time of 300 ms was used.

[1]
Y W S E N L F Q P G H

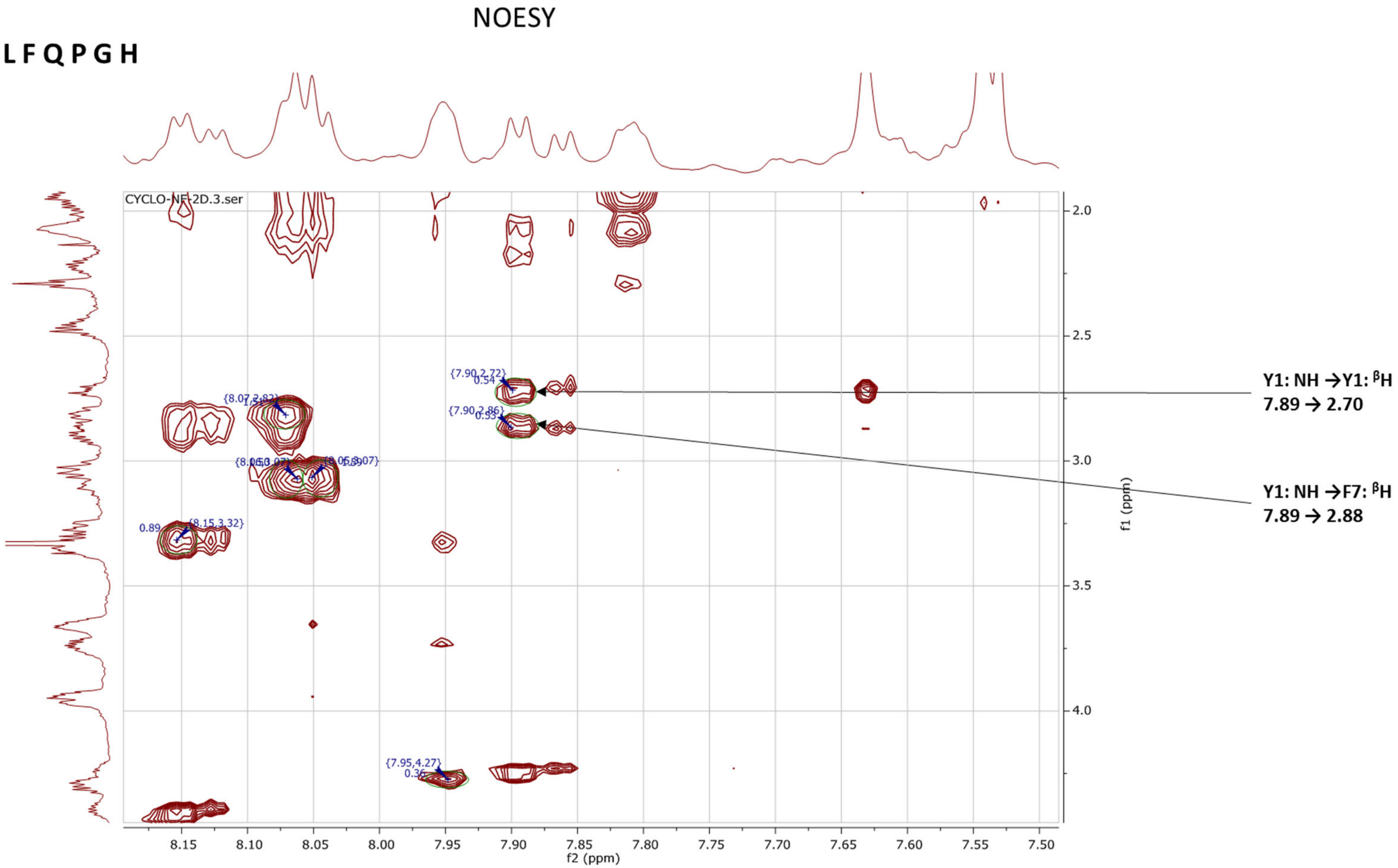

Figure S4. 9: NOESY correlations of [1] showing residue Y1 (main chain NH to βH).
Mixing time of 300 ms was used.

[1]
Y W S E N L F Q P G H

NOESY

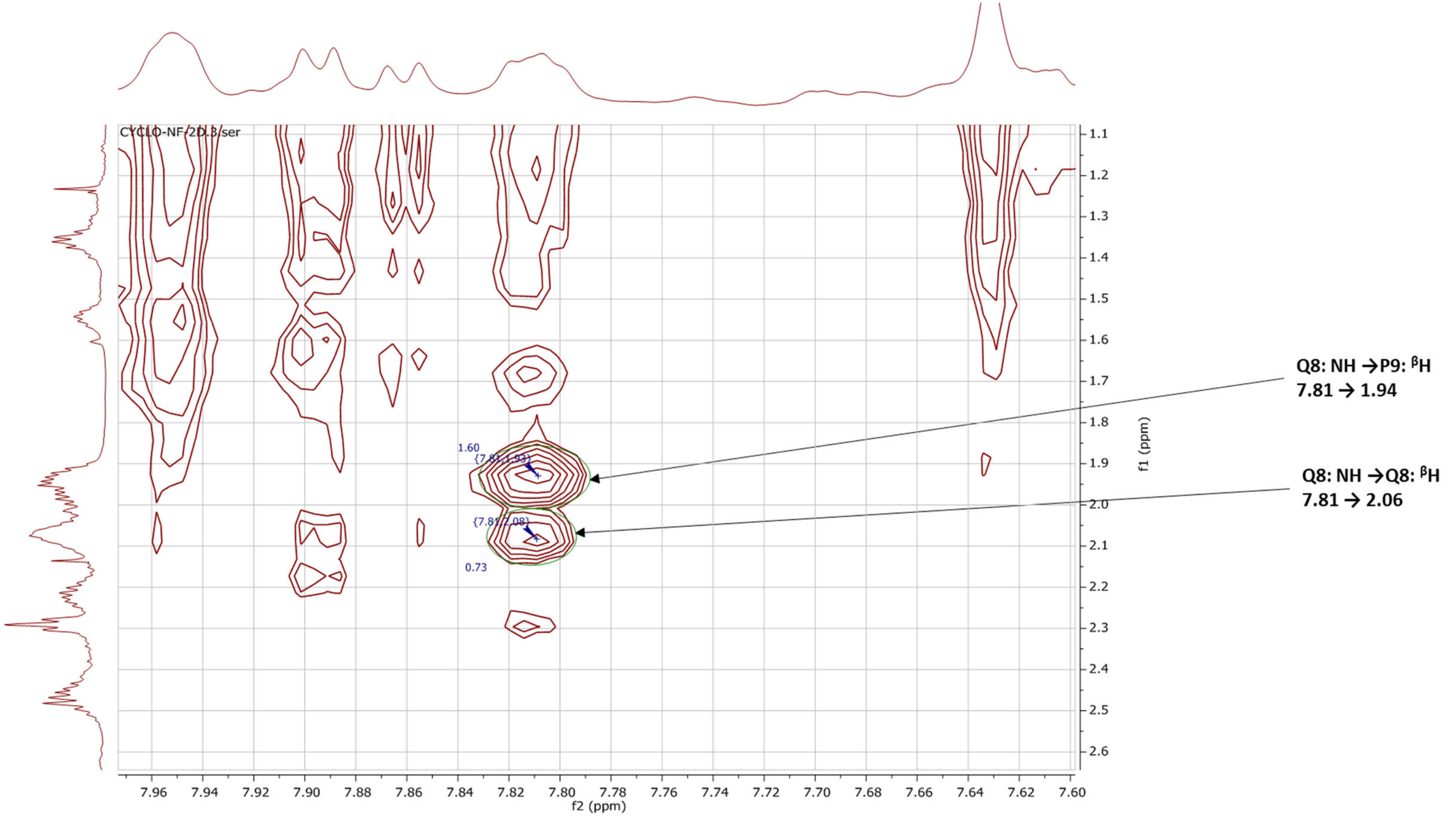

Figure S4. 10: NOESY correlations of [1] showing residue Q8 (main chain NH to βH).
Mixing time of 300 ms was used.

[1]
Y W S E N L F Q P G H

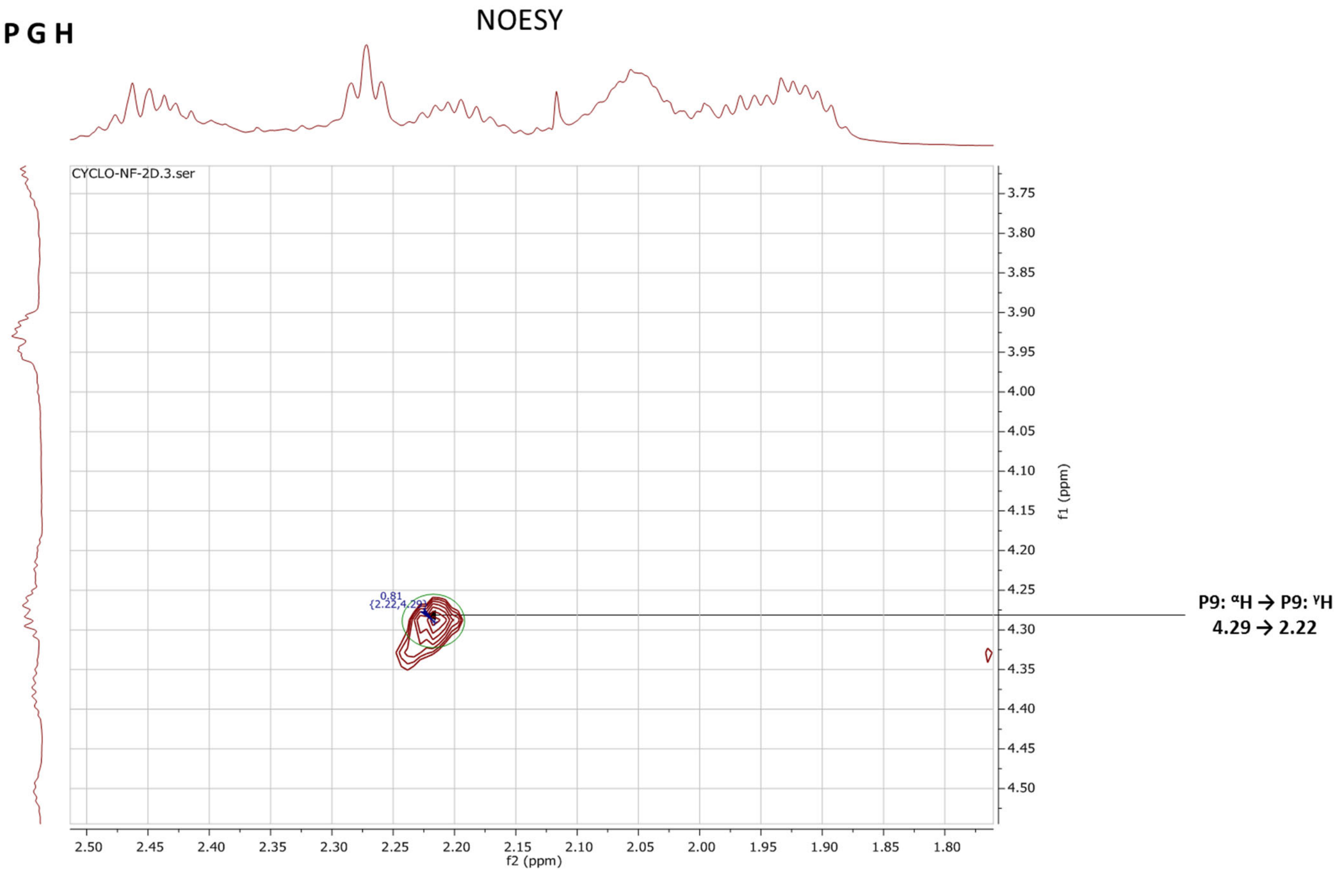

Figure S4. 11: NOESY correlations of [1] showing residue P9 (αH to γH).
Mixing time of 300 ms was used.

TNFα sensor cells

HEK-Blue TNFα cells used to test ant-TNFα activity were stable HEK293 cells transfected with a SEAP reporter gene (secretory alkaline phosphatase) downstream to IFN-β minimal promoter fused to that of NF-kB. Considering HEK293 cells are also expressing IL-1β receptor that triggers the activation of NF-kB and MAPK pathways, this cell line was rendered unresponsive to IL-1β by knocking-out MyD88 gene important for signal transduction (Figure 5.1).

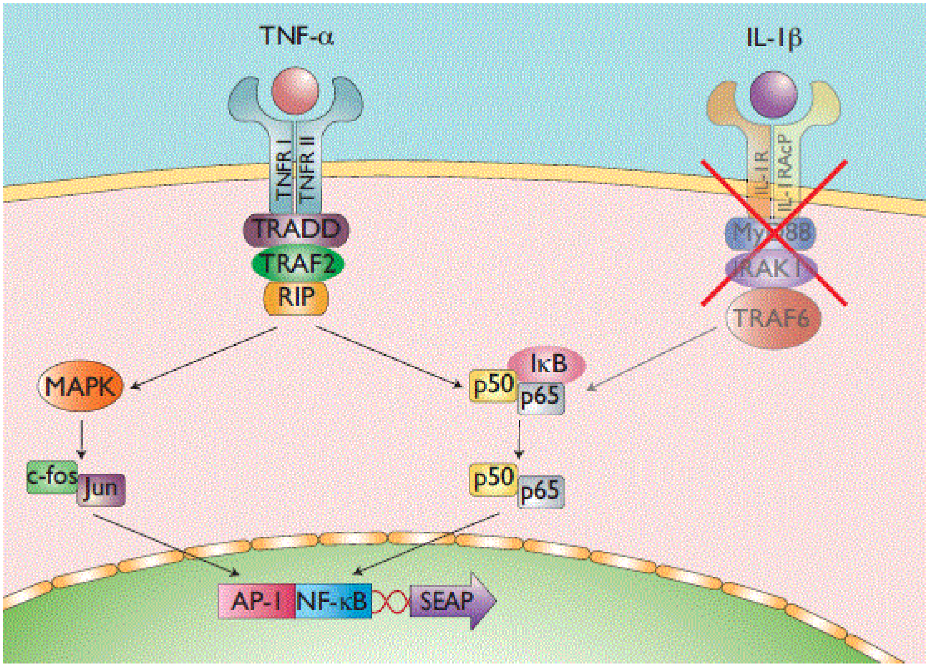

Figure S5. 1: Graphical representation of how HEK-Blue™ TNFα cells act as TNFα sensors. (Taken from InvivoGen product pamphlet).

TNFα stimulation triggers the expression of the NF-kB-induced reporter SEAP. Produced SEAP can then be quantified in cells supernatant (20 μL) by the reaction with 180 μL of QUANTI-Blue™, a reagent that turns from purple to blue in the presence of SEAP. Quantification of SEAP was done by measuring the optical density at 620 nm after 30 min incubation and the results were correlated to the level of TNFα activation.
